# Captivating bimolecular photoredox dynamics of a ligand-to-metal charge transfer complex

**DOI:** 10.1039/d5sc03839a

**Published:** 2025-10-20

**Authors:** Christina Wegeberg, Neus A. Calvet, Mila Krafft, Pavel Chábera, Arkady Yartsev, Petter Persson

**Affiliations:** a Division of Chemical Physics, Department of Chemistry, Lund University 22100 Lund Sweden wegeberg@sdu.dk arkady.yartsev@chemphys.lu.se; b Division of Computational Chemistry, Department of Chemistry, Lund University 22100 Lund Sweden petter.persson@compchem.lu.se

## Abstract

Transition metal complexes featuring ligand-to-metal charge transfer (LMCT) excited states have been identified as promising candidates for driving electron transfer processes. To obtain an efficient system based on photo-induced bimolecular electron transfer, it is required that (1) photo-induced charge separation (CS) is faster than charge recombination (CR) of the separated charges, and (2) CR is slower than their spatial separation *via* cage escape (CE). Here, we investigate this competitive sequence of processes by the photocycle of a rhenium(ii) complex featuring a strongly oxidizing ^2^LMCT excited state. Intrinsic CS and CR rates were measured up to multimolar concentrations for several electron donors to elucidate both the diffusion-controlled and close-contact regimes over a wide range of thermodynamic driving forces. Ultrafast dynamics (<200 fs) suggest that CS is dominated by a hot electron transfer component competing with ^2^LMCT vibrational relaxations in the high-concentration/close-contact regime. The intrinsic CS and CR rates related to the relaxed ^2^LMCT state dominate the dynamics at intermediate concentrations and show concentration-dependence at 3–50 vol% electron donor concentration and concentration-independence at higher concentrations with *τ*_CS_ = 0.5 ps and *τ*_CR_ = 2 ps, for the prototype donor anisole. The ratio between CS and CR rates was altered systematically by the thermodynamic driving force of electron transfer, utilizing the fact that the processes lie in the Marcus normal and inverted regions, respectively, and with deviations from classical Marcus behavior accounted for by using Marcus–Jortner–Levich theory. Our findings provide unique insight into the complex competition between kinetic factors controlling the fundamental dynamics of the charge-separated pairs inside the solvent cage for photocycles driven by ^2^LMCT excited states.

## Abstract 2

Charge recombination (CR) competes with cage escape (CE) dynamics on ultrafast timescales to determine the outcome of bimolecular photoredox processes following initial charge separation (CS) driven by a ligand-to-metal charge transfer complex.

## Graphical abstract

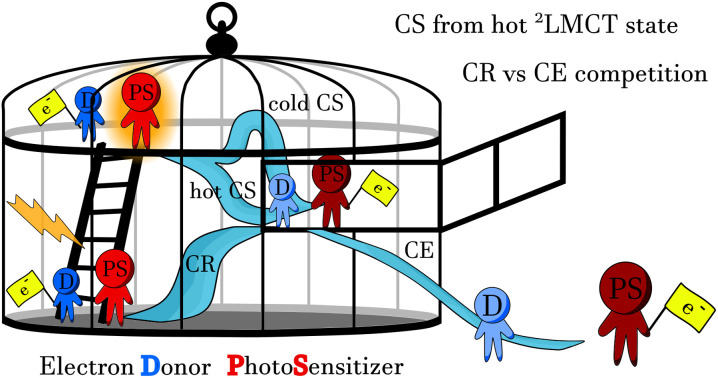

## Introduction

Photo-induced electron transfer reactions play a paramount role for reactivities in chemistry and biology. Understanding the factors controlling their rates is therefore important to ensure efficient conversion of light to chemical energy, in particular, the competition between productive forward and energy-wasting backward electron transfer rates. Bimolecular photo-induced electron transfer in the form of photoredox catalysis has, in this context, garnered renewed interest in recent years as a promising approach to utilize solar energy to generate solar fuels and other value-added products.^1–5^ Improving the overall product efficiency has spurred a revived focus on the mechanistic understanding of the initial electron transfer processes.^6–10^

Transition metal complexes of d^6^ metal ions such as ruthenium(ii) and iridium(iii) are widely used as photosensitizers in photoredox catalysis due to their attractive photophysical properties such as intense visible absorption, long metal-to-ligand charge transfer (MLCT) excited-state lifetime, and high photostability.^1–4^ Recent advances in ligand design strategies have, however, shown that promotion of ligand-to-metal charge transfer (LMCT) excited states in transition metal complexes of d^5^ metal ions can also afford photoredox-active states.^11–24^ Processes such as ground-state recovery and bimolecular electron transfer driven by the ^2^LMCT excited states in d^5^ metal complexes are spin-allowed in nature and are therefore fundamentally different from the analogous processes driven by ^3^MLCT excited states in d^6^ metal complexes. This limitation of the spin has been identified as a potential problem for the ^2^LMCT photosensitizers, as it seems to result in low cage escape yields in photocycles.^13,25–28^ Little is, however, still known about the individual steps in the ^2^LMCT-driven photocycles.

**Fig. 1:**
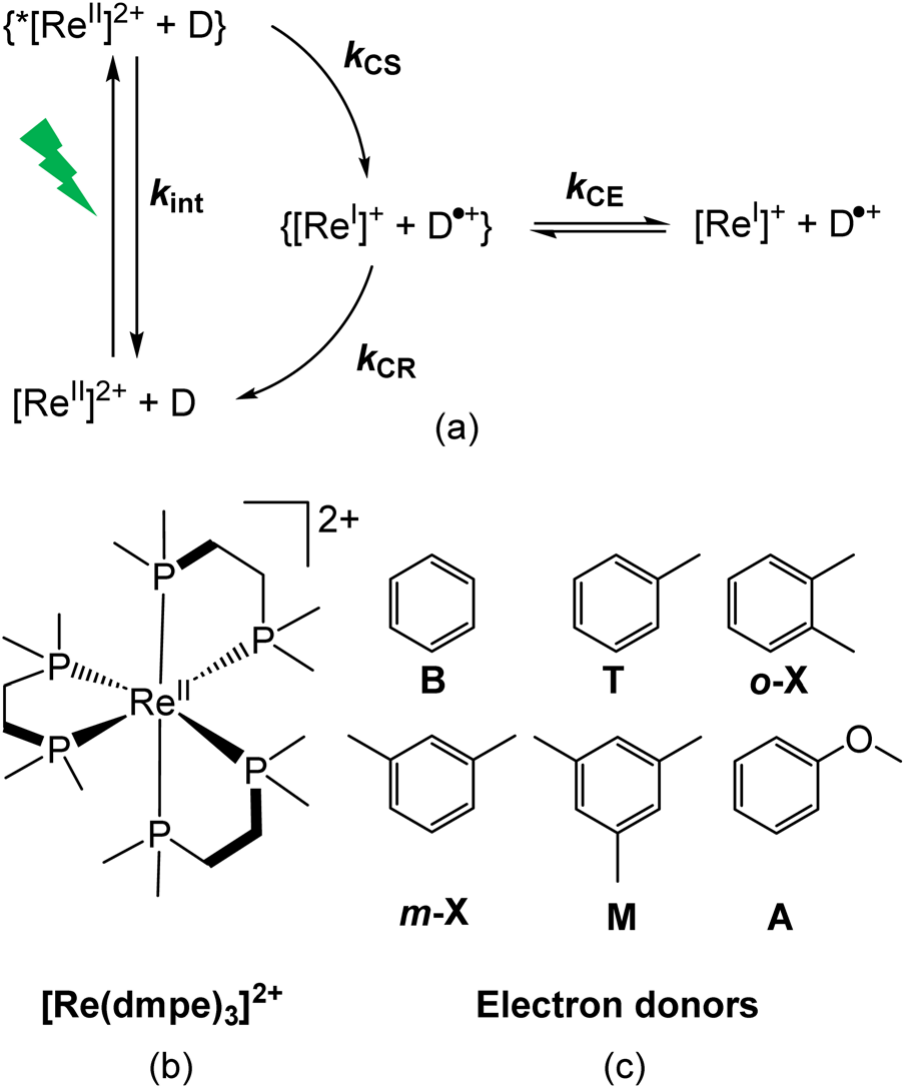
(a) Schematic reductive photocycle involving a rhenium(ii) complex, [Re]^2+^, as the photosensitizer and an electron donor (D). *K*_int_ = intrinsic decay rate of excited [Re]^2+^; *k*_CS_ = rate of CS; *k*_CR_ = rate of CR; *k*_CE_ = rate of CE. Chemical structure of (b) [Re(dmpe)_3_]^2+^ and (c) the investigated electron donors: benzene (B), toluene (T), *o*-xylene (*o*-X), *m*-xylene (*m*-X), mesitylene (M), and anisole (A).

A typical bimolecular photocycle involving a photosensitizer and a quencher molecule is initiated by excitation of the photosensitizer from its ground state to its excited state.^29^ Subsequent electron transfer between the excited photosensitizer and a nearby quencher yields the charge-separated pair inside a solvent cage. In a photocycle based on reductive quenching (Fig. 1a), the charge separation (CS) results in reduction of the photosensitizer concurrent with oxidation of the quencher. From generation of the charge-separated pair, two outcomes are possible: either the photocycle can be undesirably completed by charge recombination (CR) resulting in regeneration of the initial components, or preferably the components can spatially separate from the solvent cage in what is known as a cage escape (CE) process resulting in formation of photoproducts,^30^ useful for driving further chemical transformations. The rates of photoproduct formation have been directly linked to CE quantum yields,^31^ which also means that CE has to outcompete CR in order to obtain high photoproduct yields. CE has been estimated to take place on a timescale of hundreds of picoseconds,^28,32,33^ in accordance with the dissociative nature of the process. The competition between CE and CR requires that CR is sufficiently slow so that dissociation of the charge-separated pairs can occur.

Bimolecular photoinduced electron transfer reactions have classically been investigated through Stern–Volmer studies, where the quenching rate constant (*k*_q_) is determined from evaluation of the intensity quenching of the photosensitizer's photoluminescence in the presence of quencher.^32^ The Stern–Volmer analysis is done at low quencher concentrations where diffusion-controlled processes dominate. For this reason, Stern–Volmer studies typically do not yield the intrinsic electron transfer rate (*k*_et_), as *k*_q_ depends on both *k*_et_ and the diffusion rate constant (*k*_diff_), according to eqn (1), where *K*_A_ is the association equilibrium constant.^33,34^1
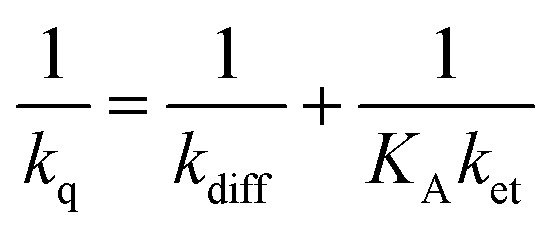


Stern–Volmer analysis thus provides indirect, but nevertheless valuable information about the generation of the charge-separated pair through CS. In contrast, it is much harder to experimentally monitor the depletion of the charge-separated pair to the ground state through CR.^35–38^ However, detailed insights into electron transfer dynamics involving the charge-separated pair are of vital importance to realize the full potential of a photocycle and to ultimately develop efficient catalytic systems.^39^ The Gibbs free energy change (Δ*G*°) has typically been a guiding principle for electron transfer reactivity,^35^ but recent spectroscopic insights highlight that the driving force (−Δ*G*°) between electron donor and electron acceptor is not alone sufficient to account for the photocatalytic behavior.^40–43^ The potential scope of bimolecular electron transfer for catalytic purposes, therefore, remains limited by a lack of fundamental understanding of the underlying mechanisms.

The intrinsic rate of electron transfer is often described by the semiclassical expression of Marcus theory (eqn (2)),^44,45^ where *k*_et_ is related to Δ*G*°, the electronic coupling between the donor and the acceptor (*H*_DA_), and the reorganization energy (*λ*). Marcus theory predicts that *k*_et_ increases with increasing driving force only to −Δ*G*° = *λ*, from where *k*_et_ decreases with increasing driving force in the so-called inverted region.2
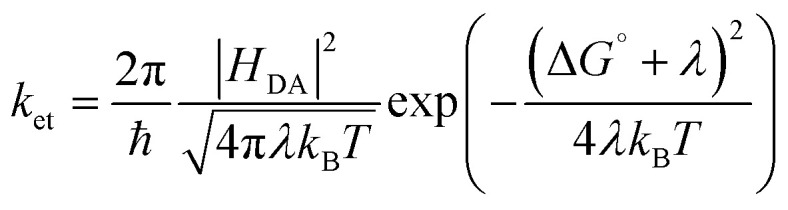
where *k*_B_ is the Boltzmann constant, *ħ* is Planck's constant, and *T* is the temperature.

As introduced by Weller and Rehm, *k*_q_ increases as the driving force rises above zero, and it reaches what appears as a plateau at *k*_q_ ∼ *k*_diff_, indicating a diffusion-controlled process.^46^ In the original study, Weller and Rehm showed that the plateau extends up to the highest achievable driving force without any indication of an inverted Marcus region. This discrepancy between Marcus theory and the Rehm–Weller behavior was, for many decades, a paradox, and the intrinsic CS dynamics were claimed to be masked by the diffusion.^47^ The original Rehm–Weller experiment was carried out using spectroscopy techniques with nanosecond time-resolution, which is partly the reason why the inverted Marcus region could not at first be observed in the data reported by Rehm and Weller. Reinvestigation of the Rehm–Weller experiments on the ultrafast timescales using differential encounter theory has since shown that a weak bell-shaped dependence of the quenching rate can indeed be observed.^33^

One strategy to circumvent any contribution from diffusion-controlled processes and directly study the intrinsic rate of electron transfer is to covalently attach the quencher to the photosensitizer. Although this intramolecular electron transfer approach was proven useful to investigate electron transfer processes in general, and to confirm the presence of the inverted Marcus region,^48–57^ it is less informative for conditions relevant to photoredox catalysis. Another strategy to study the intrinsic rates in bimolecular reactions would be to increase the concentration of the quencher sufficiently, *e.g.*, being the solvent; then, the photosensitizer and the quencher will always be in close contact. In this way, diffusion-controlled processes can be neglected and the intrinsic charge transfer rates between electron donors and electron acceptors, independent of diffusion, can be studied.^58^ Experimentally, all involved steps in the photocycle, including CR, can then be traced by ultrafast transient absorption (TA) spectroscopy at the femto- to picosecond timescale.^59–62^

In this study, we use the complex rhenium(ii) tris(1,2-bis(dimethylphosphino)ethane), [Re(dmpe)_3_]^2+^ (5d^5^, Fig. 1b), to obtain a more refined picture of the capabilities of LMCT excited states to drive reductive photocycles with a clear interest in exploiting the dynamics of the charge-separated pair. [Re(dmpe)_3_]^2+^ has a luminescent ^2^LMCT excited state with a 12 ns lifetime, which exhibits a remarkable excited-state reduction potential *E*_½_(*Re^II^/Re^I^) of +2.58 V *vs.* SCE in acetonitrile.^63–65^ In this work, we determine the intrinsic rates in the photocycle of [Re(dmpe)_3_]^2+^, *i.e.*, both electron transfers related to CS and CR processes. To do so, we work at high electron donor concentrations (up to 5.7 M) where the photosensitizer and quencher molecule are in close contact at all times, *i.e.*, far beyond the diffusion-controlled Stern–Volmer regime. The strongly oxidizing nature of the ^2^LMCT excited state of [Re(dmpe)_3_]^2+^ allows us to investigate electron donors with a span of 0.9 V in driving forces for the electron transfer. We focus on structurally similar aromatic quencher molecules (Fig. 1c), which expectedly have a weak and non-specific coupling element with the spherical and aliphatically decorated [Re(dmpe)_3_]^2+^. The transition from the ^2^LMCT excited state to the doublet ground state is spin-allowed, thus, it resembles the spin-allowed emission behavior observed for organic chromophores. However, the weaker electronic coupling between [Re(dmpe)_3_]^2+^ and the electron donors contrasts with the previous studies on organic photosensitizers, as they typically show strong coupling elements to the electron donors.^47^ We cover excited-state dynamics with TA spectroscopy from the nanosecond time regime down to ultrafast processes on the femtosecond timescale. Importantly, the ^2^LMCT excited state lifetime of 12 ns is long enough to allow quantification of slow electron transfer rates for electron donors with small driving forces and, at the same time, short enough to enable quantification of fast electron transfer rates for electron donors with large driving forces. Our studies on bimolecular photocycles with [Re(dmpe)_3_]^2+^ (Fig. 1a) were carried out in mixtures of acetonitrile. For this reason, we first investigated the excited-state dynamics of [Re(dmpe)_3_]^2+^ in acetonitrile without the presence of an electron donor. In the second part of the study, we investigate how the rates in the photocycle of [Re(dmpe)_3_]^2+^ change as a function of anisole concentration, going from diffusion-controlled dynamics in the presence of 1 vol% anisole to close-contact dynamics at 90 vol% anisole. By doing so, we identify three CS processes: (1) diffusion-controlled CS driven by the relaxed ^2^LMCT state, (2) intrinsic CS driven by the relaxed ^2^LMCT state, and (3) intrinsic CS driven by a hot ^2^LMCT state. Importantly, the high quencher concentration also allows us to directly monitor CR dynamics. In contrast with the observation of three CS processes, we only identify one CR process. In the third and last part of the study, we examine how the photoinduced electron transfer rates related to the relaxed ^2^LMCT state change as a function of driving force at a fixed electron donor concentration of 5.7 M. By covering electron transfer rates of six orders of magnitude in the photocycle of [Re(dmpe)_3_]^2+^, our study provides new insights into the dynamics of the charge-separated pair in bimolecular electron transfer reactions driven by an ^2^LMCT excited state and directly shows that the ultrafast nature of the CR counteracts the dominance of diffusion-controlled CE in this specific system.

## Results and discussion

### Excited-state dynamics of [Re(dmpe)_3_]^2+^ in the absence of electron donor

**Fig. 2:**
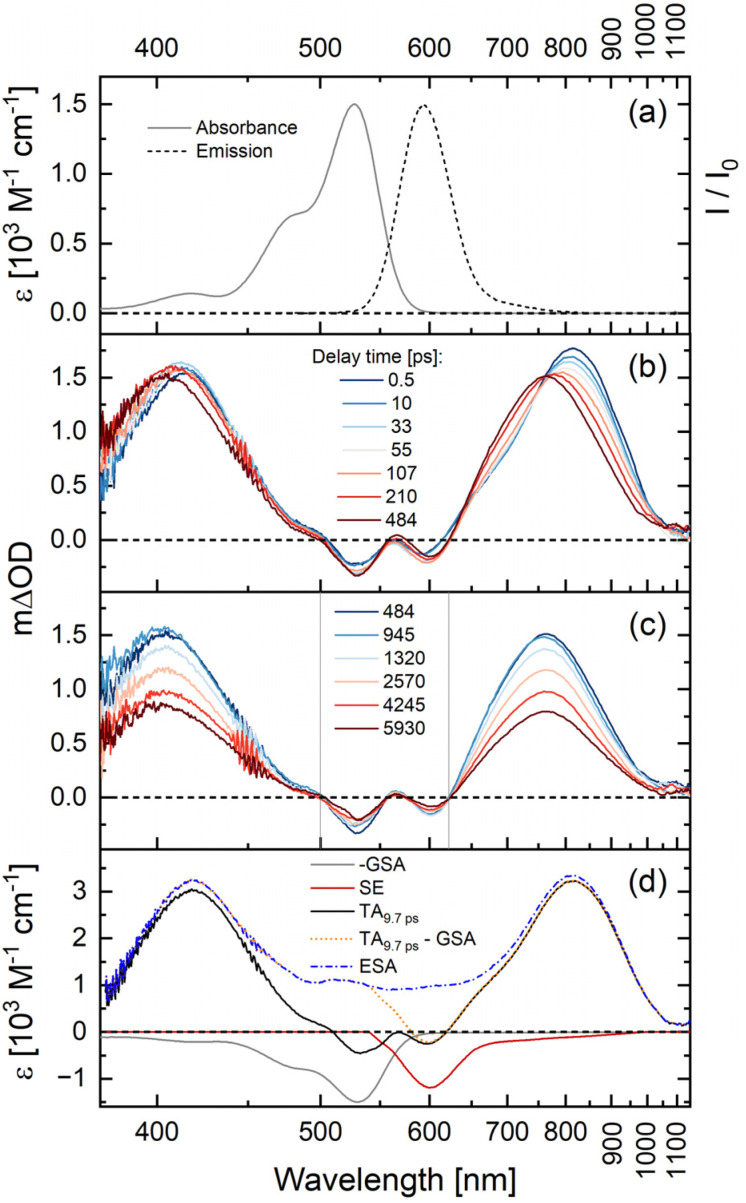
(a) Absorption and emission spectra of [Re(dmpe)_3_]^2+^ in deaerated acetonitrile. Excitation wavelength set to 450 nm. The ^2^LMCT absorption and emission band maxima of [Re(dmpe)_3_]^2+^ are found at 527 nm and 592 nm, respectively. TA spectra of [Re(dmpe)_3_]^2+^ in deaerated acetonitrile at (b) picosecond and (c) nanosecond timescales. Delay times are given in picoseconds, and their corresponding color codings are shown in the insets. The gray vertical lines indicate isosbestic points. Excitation occurred at 540 nm with 90° between the pump and probe polarization. (d) The ESA spectrum (blue) is estimated by subtracting contributions from scaled GSA (gray) and scaled SE (red) from the TA spectrum recorded at a delay time of 9.7 ps (black). The spectrum of the SE was calculated from the spectrum of the spontaneous emission seen in (a). The *x*-axis is shown on a reciprocal wavelength scale, so the spectra can be displayed linearly on an energy scale.

In this first part of our study, we perform a detailed analysis of the excited-state dynamics of [Re(dmpe)_3_]^2+^ in acetonitrile, as this serves as a reference point for the measurements of the bimolecular photocycles of [Re(dmpe)_3_]^2+^. Upon excitation of [Re(dmpe)_3_]^2+^, we identify the following spectral features in the TA spectra: a ground state bleach (GSB) at 530 nm, excited-state absorptions (ESAs) at 415 and 800 nm, as well as stimulated emission (SE) at 600 nm (Fig. 2b). There is a good correlation between the GSB and SE positions and the ^2^LMCT absorption and emission band maxima of [Re(dmpe)_3_]^2+^, respectively (Fig. 2a).^63–65^ The ESAs at 415 and 800 nm blueshift to 405 and 760 nm, respectively, within the first hundreds of picoseconds without any ground-state recovery occurring. No isosbestic points are identified during this process, and the blueshift is observed both with perpendicular (90°) and magic angle (54.7°) between pump and probe polarization (Fig. S15 and S16). This blueshift is too slow for vibrational relaxation, suggesting that it is instead related to a more significant change of excited state character, *e.g.*, a conformational change of [Re(dmpe)_3_]^2+^. Recovery of the ground state takes place on the nanosecond timescale and is associated with two isosbestic points at zero differential absorption (Fig. 2c). On the nanosecond timescale, the ESAs at 405 and 760 nm, as well as the SE at 600 nm, decay with the same time constant. The same slow dynamics in the entire recorded spectral region, together with the observation of isosbestic points at zero differential absorbance, support that the ESA and SE originate from the same excited state. Global fit analysis determined time constants of 200 ps and 9 ns (Fig. S17). The slower time constant from our TA measurements (9 ns) is in good agreement with the previously reported 12 ns luminescence lifetime of [Re(dmpe)_3_]^2+^ in acetonitrile,^64^ in particular taking the available instrumental time window of 8 ns into account.

In general, a TA spectrum represents the sum of contributions from GSBs, ESAs, and SE. By subtracting a scaled GSB and SE from the TA data, we generated the “pure” ESA spectrum of [Re(dmpe)_3_]^2+^ (see details in SI). The ESA spectrum can provide further insight into photoinduced electron transfer processes, and a comparison of the TA and calculated ESA spectra of [Re(dmpe)_3_]^2+^ clearly shows that ESA contributes strongly to the overall shape of the TA signal in the entire spectral region. The ESAs at 415 and 800 nm are solely dominated by ESA contributions for excited [Re(dmpe)_3_]^2+^. Changes to the ESA amplitude/shape at 415 and 800 nm in the presence of electron donor can therefore give valuable and direct information on electron transfer processes between excited [Re(dmpe)_3_]^2+^ and electron donors (*vide infra*).

### Electron donor concentration dependence

**Fig. 3:**
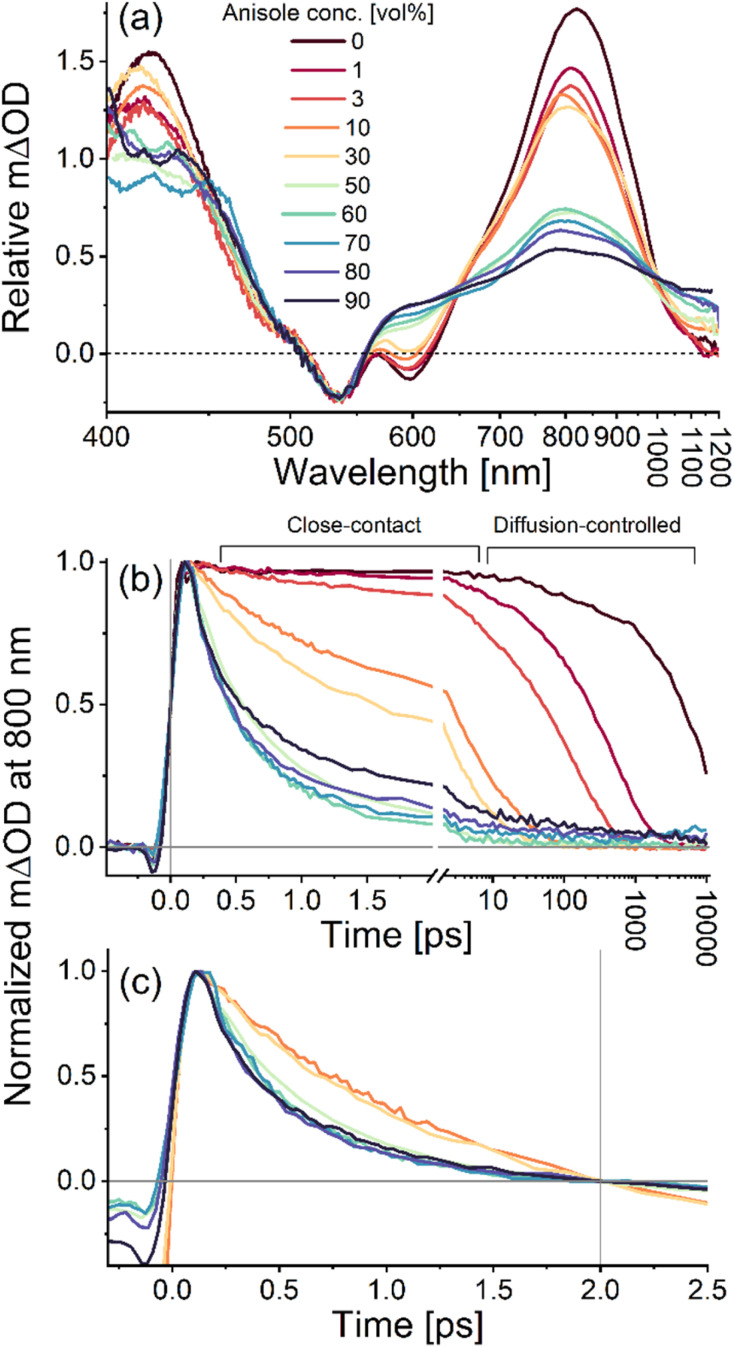
Excited-state dynamics of [Re(dmpe)_3_]^2+^ in deaerated mixtures of acetonitrile and anisole. (a) First time-resolved TA spectra at a delay time of 200 fs after the pump pulse. The intensity of the spectra in presence of anisole has been scaled relative to the amplitude of the GSB of the spectrum in absence of anisole, using the GSB amplitude and the shoulder at 480 nm as reference point. (b) Normalized kinetic trace for the differential absorption at 800 nm. (c) Normalized kinetic trace for the differential absorption at 800 nm referenced to the delay time of 2 ps at high anisole concentration. The concentration of anisole in vol% and the corresponding color coding is seen in the insert of (a). All datasets were obtained with excitation at 540 nm and 90° between the pump and probe polarization.

In the second part of our study, we examine how rates in the photocycle of [Re(dmpe)_3_]^2+^ change as a function of anisole concentration (Table 1, Fig. 3). We focus on anisole as the electron donor because of the large driving force for the photoinduced electron transfer between anisole and [Re(dmpe)_3_]^2+^ (Δ*G*_CS_ = −0.82 V). In addition, [Re(dmpe)_3_]^2+^ has a conveniently high solubility and photostability in anisole/acetonitrile mixtures. The concentration of [Re(dmpe)_3_]^2+^ was fixed to 3.2 mM in all our measurements, and we considered anisole concentrations between 1 vol% and 90 vol%. It was not possible to dissolve [Re(dmpe)_3_]^2+^ in solutions with anisole concentrations above 90 vol%. In photoredox-catalyzed reactions, a typical photosensitizer concentration is 1–5 mol% relative to the substrate.^66–68^ In our [Re(dmpe)_3_]^2+^ system, this typically used ratio between photosensitizer and anisole is represented by the mixtures containing anisole concentrations of 1 vol% and 3 vol%.

**Table 1:** Selected parameters and time constants in the photocycle of [Re(dmpe)_3_]^2+^ related to the relaxed ^2^LMCT state carried out in deaerated mixtures of acetonitrile and anisole at 20 °C^a^

Anisole [vol%]	Anisole [M]	*d*_ReED_ [Å]	*τ*_CS,diff_ [ps]	*τ*_CS,relax_ [ps]	*τ*_CR_ [ps]
1	0.094	13	560	N/A	30
3	0.28	9.0	170	7	30
10	0.94	6.0	30	0.8	4
30	2.8	4.2	N/A	0.6	4
50	4.7	3.5	N/A	0.5	2
60	5.7	3.3	N/A	0.5	2
70	6.6	3.2	N/A	0.5	2
80	7.5	3.0	N/A	0.5	2
90	8.5	2.9	N/A	0.5	2

a *d*_ReED_: average center-to-center distance between [Re(dmpe)_3_]^2+^ and the electron donor; *τ*_CS,diff_: time component associated with CS based on diffusion-controlled processes; *τ*_CS,relax_: time component associated with intrinsic CS based on close-contact pairs from the relaxed ^2^LMCT excited state; *τ*_CR_: time component associated with charge recombination.

Steady-state absorption spectra of [Re(dmpe)_3_]^2+^ showed that the ^2^LMCT absorption band is only weakly influenced by the addition of high concentrations of anisole (Fig. S10). Time-dependent density functional theory (TD-DFT) calculations have previously suggested that the dominant LMCT transition of [Re(dmpe)_3_]^2+^ is highly symmetric and delocalized, *i.e.*, involving all phosphine ligand donors.^69^ These theoretical insights on the symmetric electronic structure of [Re(dmpe)_3_]^2+^ in the ground state help to explain why a solvatochromic effect, normally expected for absorption bands of charge transfer character, is not pronounced in our experimental data. New absorption bands are also not appearing at high anisole concentrations, which indicates that the electronic coupling between [Re(dmpe)_3_]^2+^ and anisole is weak. This behavior contrasts with photoredox studies based on organic photosensitizers, where interactions between the photosensitizer and aromatic quenchers have resulted in new absorption and/or emission bands from intraligand charge transfer bands.^47,70,71^

Photo-induced electron transfer is the only relevant quenching mechanism in our work, as quenching *via* Förster-type energy transfer is unlikely due to an energy mismatch between [Re(dmpe)_3_]^2+^ and the investigated electron donors (Table S2). Furthermore, Dexter-type doublet-to-triplet energy transfer has recently appeared in the photochemical community as a viable energy transfer mechanism,^72–74^ but the triplet energies of the investigated quenchers are higher than the energy of the ^2^LMCT state in [Re(dmpe)_3_]^2+^ hence, also Dexter-type energy transfer is improbable.

By using TA spectroscopy, we trace the entire photocycle of [Re(dmpe)_3_]^2+^ with all involved steps, *i.e.*, CS, CR, and potential CE. This approach thus provides significantly more mechanistic details compared to the investigation of excited-state quenching by emission spectroscopy, where only the CS process is reflected. In particular, this also means that we directly monitor the dynamics of the charge-separated pair, {[Re^I^]^+^ + A˙^+^} (Fig. 1a). [Re(dmpe)_3_]^+^ only absorbs at wavelengths below 300 nm (Fig. S8), thus we cannot observe any spectral features related to the generation of [Re(dmpe)_3_]^+^ within our investigated spectral window. Using spectroelectrochemical methods, we, however, generated the absorption spectrum of the anisole radical cation, A˙^+^ (Fig. S78), showing an absorption band between 300 and 450 nm with tailing absorbance to ∼700 nm. Structurally similar aromatic radical cations have also been shown to absorb between 400 nm and 450 nm.^75,76^

The lowest quencher concentration we investigate is 1 vol% anisole (Fig. 3), which is equivalent to an anisole concentration of ∼100 mM. In a classic Stern–Volmer study, this would be suitable as an upper quencher concentration, because the excited-state dynamics at this anisole concentration remain dominated by diffusion as the quenching only occurs on the nanosecond timescale (red trace in Fig. 3b does not decay on sub-10 ps timescale). In the presence of 1 vol% anisole, the excited-state lifetime of [Re(dmpe)_3_]^2+^ is reduced to 560 ps due to diffusion-controlled photoinduced CS (*τ*_CS,diff_) (Fig. S21). The SE at 600 nm is furthermore observed at all delay times (Fig. S19), which indicates that the photogenerated excited state of [Re(dmpe)_3_]^2+^ is the longest-lived species in the photocycle at 1 vol% anisole, which is only possible if CS is followed by rapid CR (*k*_CR_ ≫ *k*_CS_). This behavior also means that there will be no build-up of the charge-separated pair, {[Re^I^]^+^ + A˙^+^} (Fig. 1a). Comparison of the ESA kinetic traces at 415 nm and 800 nm, however, reveals that they do not have the same dynamics on the tens-of-picoseconds timescale (Fig. S20). As the anisole radical cation only has absorption at 415 and not at 800 nm (Fig. S78), the observed deviation in the kinetic traces at 415 nm and at 800 nm is likely to reflect the relationship between CS and CR. Under this assumption, the CR lifetime (*τ*_CR_) can be attributed to a time constant of 30 ps (Fig. S22), which is 20 times faster than CS at a quencher concentration of 1 vol% anisole.

Increasing the anisole concentration to 3 vol% reduces the excited-state lifetime of [Re(dmpe)_3_]^2+^ to 170 ps due to diffusion-controlled photoinduced CS (*τ*_CS,diff_) (Fig. S25). An additional faster photoinduced CS component on the few-picoseconds timescale is moreover observed at 3 vol% (orange trace in Fig. 3b decays on the sub-10 ps timescale). Based on a Poisson distribution (described in the SI in more detail), the probability of at least one anisole molecule being in close contact with [Re(dmpe)_3_]^2+^ at 3 vol% anisole is 45% (Table S1), which explains why CS independent of diffusion is observed. The time component connected to this close-contact quenching (*τ*_CS,relax_) was determined to be 7 ps (Fig. S25). Based on the pre-exponential factors of the fitting function, the close-contact quenching is estimated to account for ∼40% of the electron transfer events, quenching the excited state of [Re(dmpe)]^2+^. The remaining ∼60% of the excited state is quenched by diffusion-controlled dynamics with a CS time component (*τ*_CS,diff_) of 170 ps in the presence of 3 vol% anisole. The time component related to CR was estimated to be 30 ps (Fig. S26), which means that CR is one order of magnitude faster than diffusion-controlled CS. The fact that the time component of CR is 30 ps both in the presence of 1 vol% and 3 vol% anisole indicates that the electronic coupling element for CR is the same at these relatively low electron donor concentrations.

**Fig. 4:**
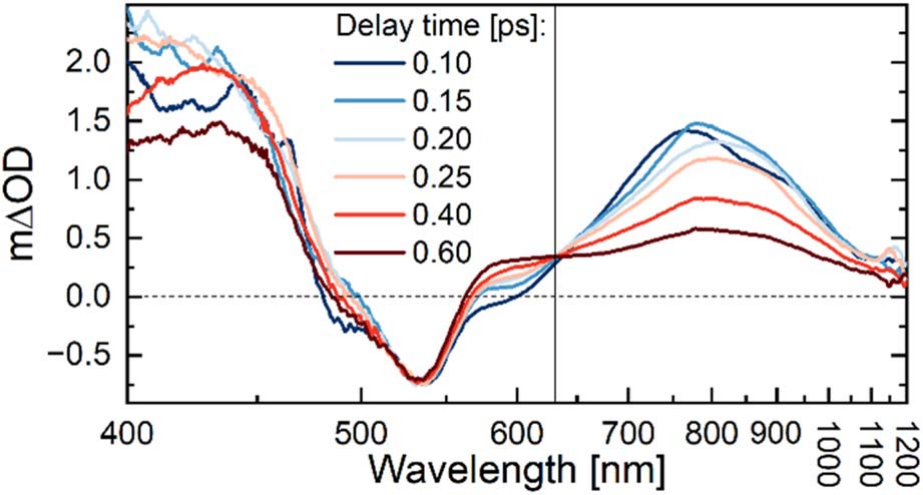
TA spectra of [Re(dmpe)_3_]^2+^ in an acetronitrile : anisole mixture (40 : 60 vol%). The isosbestic point at 630 nm (black vertical line) is associated with the formation of the charge-separated pair {[Re^I^]^+^ + A˙^+^}. Delay times in picoseconds and the corresponding color coding are displayed in the inset. The *x*-axis is shown on a reciprocal wavelength scale in order to display the spectra linearly on an energy scale. Excitation occurred at 540 nm with 90° between the pump and probe polarization.

The spectral features of the TA spectra of [Re(dmpe)]^2+^ change significantly as the quencher concentration is increased to 10 vol% or higher (Fig. 3a). Importantly, an isosbestic point at 630 nm is identified within the first few picoseconds (Fig. 4, S27, S30, S33, S39, S42, and S45). Observation of an isosbestic point equals identification of consecutive processes, which suggests that the rate of CS is not the rate-determining step in the photocycle of [Re(dmpe)_3_]^2+^ at high anisole concentrations. This, therefore, also means that the rate of CS must be faster than the rate of CR (*k*_CS_ > *k*_CR_). The absorption spectrum of the anisole radical cation, A˙^+^, has significant absorption between 600–650 nm (Fig. S78), thus the isosbestic point at 630 nm can be taken as a marker for the build-up of the charge-separated pair, {[Re^I^]^+^ + A˙^+^}, during which the amplitude of the GSB signal is unchanged (Fig. 4).

**Fig. 5:**
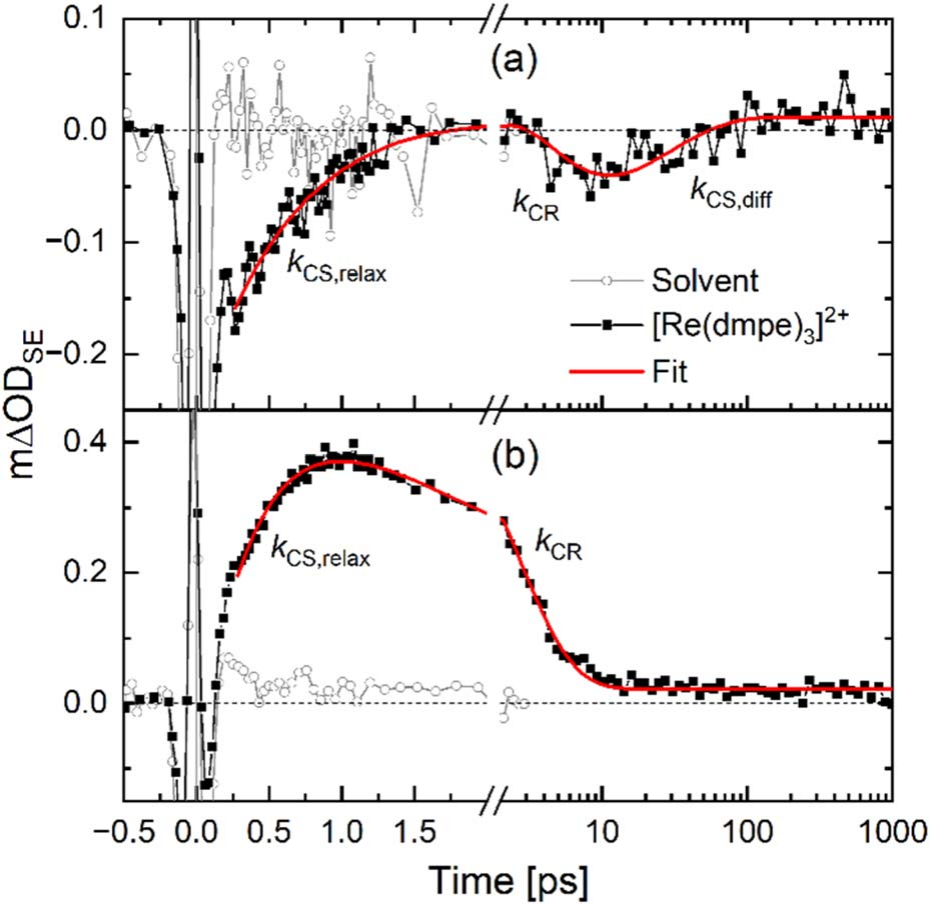
Kinetic traces (black, squares) monitoring the SE at ∼600 nm for [Re(dmpe)_3_]^2+^ in acetronitrile:anisole mixtures with either (a) 10 vol% anisole or (b) 60 vol% anisole. The solvent response (gray, circles) is included to judge at what time point the excited-state dynamics are free of coherent artifacts and from where reliable fitting (red trace) can be done at the ultrafast timescales. Rate constants for various identified electron transfer processes are noted (*k*_CS,relax_, *k*_CS,relax_, *k*_CR_); further information on the fitting procedure of these rates can be found in the SI. The data was obtained with excitation at 540 nm and 90° between the pump and probe polarization. The datasets of [Re(dmpe)_3_]^2+^ and of the solvent response were measured back-to-back under identical instrument settings, enabling direct intensity comparison.

The SE at 600 nm is a unique fingerprint of the excited state of [Re(dmpe)_3_]^2+^ as it can easily be identified and calculated from the steady-state emission spectrum (*i.e.*, from spontaneous emission, Fig. 2). This, in addition to the fact that the anisole radical cation also has absorption in this spectral region (Fig. S78), means that the formation of the charge-separated pair can be monitored by the dynamics at 600 nm, because CS translates to a signal rise at 600 nm which subsequently decays during the CR process (Fig. 5). The signal intensity at 600 nm will increase as long as the CS process generates charge-separated pairs faster than their depletion due to CR or CE. In this way, the kinetic trace at 600 nm gives unique insights into the dynamics of the charge-separated pairs inside the solvent cage. From this perspective, the quencher concentration of 10 vol% anisole is interesting, because both CS based on close-contact and diffusion-controlled quenching, as well as CR, are directly observed in the kinetic data at 600 nm (Fig. 5a). Initially, the kinetic trace at 600 nm increases due to close-contact CS (*τ*_CS,relax_ = 0.8 ps) generating charge-separated pairs, which subsequently recombine (*τ*_CR_ = 4 ps). At this point, the part of the excited-state population that was quenched by the close-contact CS has returned to the ground state. Another part of the excited-state population of [Re(dmpe)_3_]^2+^ has not yet undergone CS because no anisole molecules were in close contact with the photosensitizer. For this reason, the kinetic trace at 600 nm returns to a negative amplitude related to SE from the unquenched [Re(dmpe)_3_]^2+^ population, from where it decays to zero differential absorption as diffusion-controlled CS quenches the remaining part of the excited-state population (*τ*_CS,diff_ = 30 ps). The close-contact CS accounts for ∼50% of the excited-state quenching at 10 vol% anisole. Compared to the 40% of the quenching events at 3 vol% anisole, the close-contact quenching therefore becomes more dominant with quencher concentration and at anisole concentrations higher than 10 vol%, diffusion-controlled CS is no longer observed in the excited-state dynamics and close-contact CS on the picosecond timescale is the dominating quenching process (Fig. 5b). This observation agrees well with the Poisson distribution which estimates that the probability for at least one anisole molecule to be within the solvent shell of [Re(dmpe)_3_]^2+^ is 100% at 30 vol% anisole or higher (Table S1).

The rate of the close-contact CS increases as a function of anisole concentration from 3 to 50 vol% (Table 1). Within this range of anisole concentrations, the rates of CR also increase as a function of anisole concentration. The average center-to-center distance (*d*_ReED_ in Table 1) decreases from 9.0 Å at 3 vol% to 3.5 Å at 50 vol% anisole, and the probability of at least one anisole molecule being within the solvent shell of [Re(dmpe)_3_]^2+^ increases from 45% at 3 vol% anisole to 100% at 50 vol% (Table S1). At anisole concentrations higher than 50 vol%, both the rate of the time-resolved close-contact CS (Fig. 3c) and the CR rate (Fig. S49) become independent of the electron donor concentration from which their intrinsic lifetimes can be determined to 0.5 ps and 2 ps, respectively (Table 1). Therefore, it seems that the rates in the photocycle speed up, not only because there is a more direct contact between [Re(dmpe)_3_]^2+^ and anisole at increasing anisole concentration (minimizing contribution from diffusion-controlled CS at high anisole concentrations), but also because the effective electronic coupling element increases to reach a saturation plateau. For this reason, the excited-state dynamics at intermediate concentrations are governed by a distribution of electronic coupling elements, where the increasing rates upon increasing anisole concentration reflect a gradual evolution from the diffusion-controlled regime at one extreme to the close-contact regime at the other extreme.

Interestingly, the ratio between *τ*_CS,relax_ and *τ*_CR_ remains ∼4 throughout the entire investigated concentration span. This could be correlated to the picosecond nature of the two processes, because even though there is a distribution of coupling elements between [Re(dmpe)_3_]^2+^ and nearby distributed anisole molecules for CS, the CR that we observe must originate from geminate charge pairs with the same mutual distribution, as there is no time for the molecules to rearrange on this fast timescale to be involved in non-geminate CR. Consequently, the distribution of the electronic coupling element for CR will be governed by the preceding CS, leading to a fixed relationship between the two rates. This picture also explains why we observe concentration dependence on the CR rates, because whereas geminate CR does not depend on diffusion, it depends on the electronic coupling element between the two reactants. In other words, our experiments suggest that the rates of CS and CR are coupled on sufficiently short timescales, where the CS biases the structural configuration of CR. This connection between CS and CR would naturally be lost on slower timescales, as diffusion and thereby non-geminate CR become a viable option.

A comparison of the first time-resolved TA spectra free of solvent artifact recorded 200 fs after the excitation pulse at different anisole concentrations (Fig. 3a) shows that the contribution of the negative SE feature at 600 nm gradually decreases with increasing anisole concentration and effectively disappears at high concentrations. A related trend is observed at 800 nm, where the earliest resolved TA amplitude also decreases with increasing anisole concentration. This observation indicates that a part of the excited-state population of [Re(dmpe)_3_]^2+^ undergoes CS before 200 fs, which is the limit of time resolution of these broadband TA measurements. The TA amplitude at 800 nm is dominated by ESA related to excited [Re(dmpe)_3_]^2+^ (Fig. 2d), hence the relative TA amplitude in the absence and presence of electron donor can be used to evaluate the quenching of the excited [Re(dmpe)_3_]^2+^ and hereby the significance of the unresolved CS component. Interestingly, the ratio between the CS process that we do (Table 1, *τ*_CS,relax_) and do not time-resolve depends on anisole concentration, and the unresolved CS process becomes increasingly more pronounced with increasing anisole concentration (Fig. S48). Comparison of the TA signal intensities at 800 nm suggests that the unresolved CS process accounts for at least half of the excited-state quenching at anisole concentrations of 50 vol% and higher (Fig. S50).

**Fig. 6:**
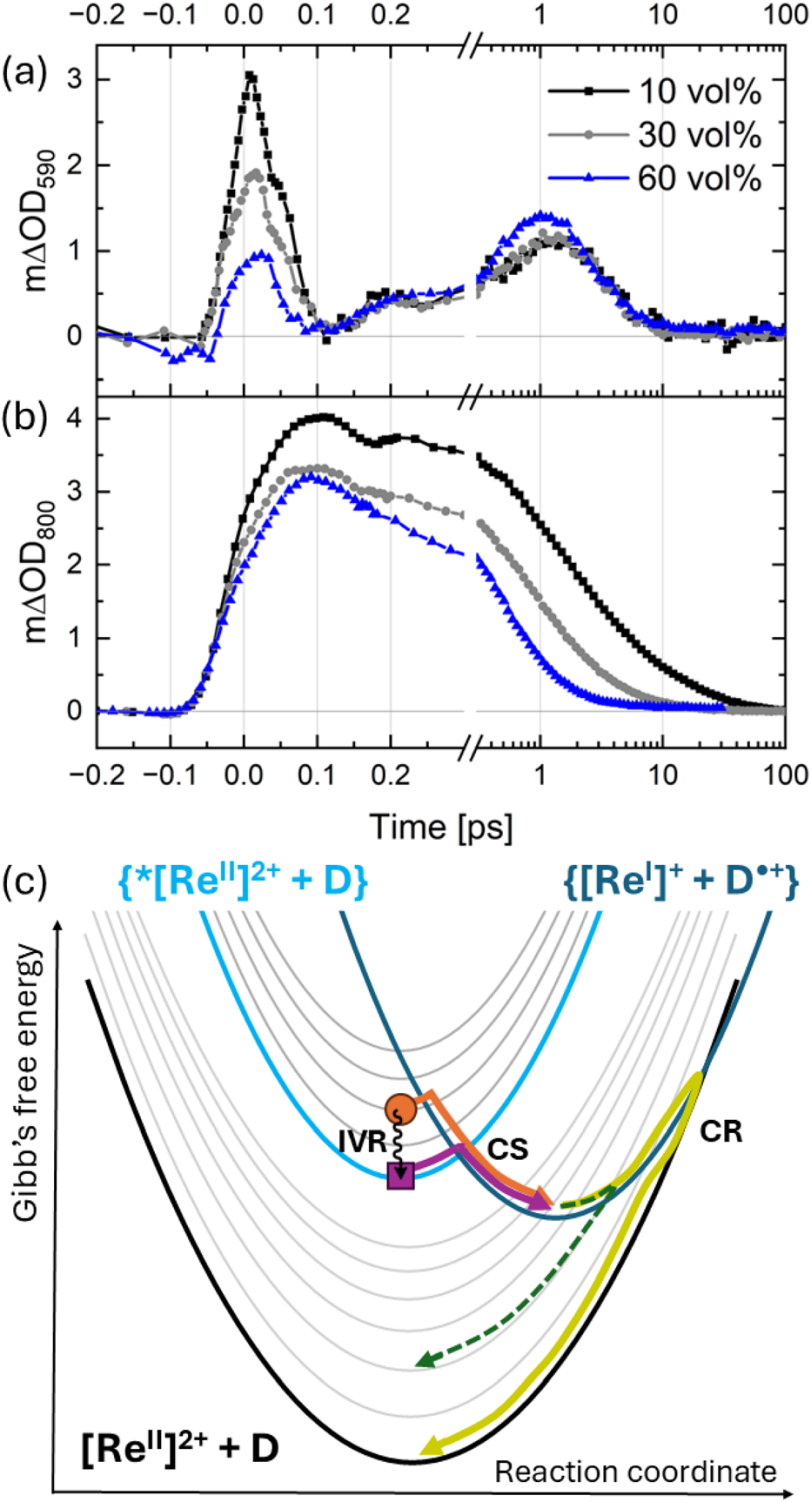
Kinetic traces for the differential absorption at (a) 590 nm and at (b) 800 nm for [Re(dmpe)_3_]^2+^ in acetonitrile/anisole mixtures, where the concentration of anisole is 10 vol% (black), 30 vol% (gray), and 60 vol% (blue). The excitation wavelength was set to 530 nm. (c) Schematic diagram showing the crossing points between Gibbs free energy surfaces for the ground state [Re^II^]^2+^ + D (black), excited photosensitizer state {*[Re^II^]^2+^ + D} (turquoise), and the separated pair {[Re^I^]^+^ + D˙^+^} (dark blue). Molecular vibrational levels of [Re^II^]^2+^ + D and {*[Re^II^]^2+^ + D} are included as shifted gray parabolas. The processes of IVR (wavy black arrow) from hot ^2^LMCT state (orange circle) to relaxed ^2^LMCT state (purple square), hot CS (CS, orange arrow), relaxed CS (CS, purple arrow), and CR (CR, yellow) are indicated. The green dashed line indicates Marcus–Jortner–Levich-type CR, where higher vibrational levels of the ground state facilitate faster CR than expected from classical Marcus theory.

To get more insights into the ultrafast CS, we performed single-wavelength femtosecond TA spectroscopy studies with a temporal resolution of ∼40 fs. In this analysis, we focus on the dynamics probed at 590 nm (Fig. 6a) and at 800 nm (Fig. 6b) as these spectral regions were identified above as key spectral features associated with the relaxed ^2^LMCT state. When interpreting TA spectroscopy results of photocycles on the ultrafast timescale (<200 fs), it is important to consider the spectral changes related to excited-state relaxation processes in addition to those induced by fast CS processes. Excited-state relaxation dynamics are usually observed as a redshift of the SE signal and may additionally lead to a spectral shift of ESA, resulting in a delayed build-up or a partial fast decay of SE and ESA at specific wavelengths. If we assume that the generation of the relaxed state of [Re(dmpe)_3_]^2+^ is much faster than CS, then CS processes will solely occur from the relaxed ^2^LMCT state. Furthermore, if the relaxation would be much faster than the time resolution of the experiment we would observe an instrument response-limited build-up of the signals at 590 nm (a combination of the SE and ESA contributions, see Fig. 2d) and at 800 nm (ESA) to their maximum amplitudes, followed by a fast decay of both signals due to depopulation of the ^2^LMCT excited state induced by CS. Such CS behavior solely from the relaxed ^2^LMCT state is, however, not supported by our data. Instead, we observe a build-up of the ESA at 800 nm that is clearly delayed compared to the pulse-limited rise (Fig. 6b) whereas the signal at 590 nm first decays within 100 fs and subsequently increases in amplitude (Fig. 6a). As we could not obtain the ultrafast dynamics of [Re(dmpe)_3_]^2+^ in pure acetonitrile with single wavelength femtosecond TA spectroscopy due to significant photodegradation under these conditions (Fig. S76), we instead evaluated the expected behavior in pure acetonitrile from the TA spectra recorded with broadband TA spectroscopy. The TA spectra of [Re(dmpe)_3_]^2+^ in acetonitrile shows that the ESA contributes strongly to the spectral region around 590 nm (Fig. 2d). The decay of the 590 nm signal within the first 100 fs in the presence of anisole (Fig. 6a) is therefore induced by a build-up of the negative signal related to SE from the relaxed ^2^LMCT state on top of the positive ESA signal. In other words, the positive ESA dominates the TA amplitude at 590 nm before the SE signal has completed its redshift due to intramolecular vibrational energy redistribution (IVR) within the ^2^LMCT manifold. Together, the observations at 800 nm and 590 nm on the ultrafast timescales therefore suggest that we temporally resolve relaxation from a hot ^2^LMCT state to a relaxed ^2^LMCT state. On the very fast timescale (∼100 fs),^77–79^ IVR within the ^2^LMCT manifold is not expected to be significantly influenced by the environment, nor in particular, by electron donor concentrations. Therefore, if the early decay at 590 nm and the rise at 800 nm are caused by IVR, they should look the same for different electron donor concentrations. However, in our measurements, the kinetic traces measured at the same concentration of [Re(dmpe)_3_]^2+^ and excitation density but at different anisole concentrations (10 vol%, 30 vol%, and 60 vol%) clearly show that the early ESA at 590 nm and 800 nm decrease with the quencher concentration (Fig. 6a and b). This implies that the corresponding concentration of the excited [Re(dmpe)_3_]^2+^ is lowered by anisole through reduction to [Re(dmpe)_3_]^+^. We do not resolve this ultrafast electron transfer process as it apparently occurs faster than the experimental temporal resolution and on the same timescale or faster than IVR. Such a fast CS therefore competes with IVR in depopulating the hot excited state, which also means that a part of the initially excited complexes is involved in this ultrafast CS and never develops into a relaxed ^2^LMCT state. The increase of this hot CS under higher quencher concentration can be linked to an increased likelihood of an anisole molecule being in close proximity to an excited [Re(dmpe)_3_]^2+^ and thus enabling hot CS. From our data, it is not possible to determine the individual rates of IVR and the hot CS process. The two rates must, however, be of similar magnitude, because no preferential deactivation channel of the hot ^2^LMCT excited state seems to dominate the observed excited-state dynamics. Taking into account the instrument response function (IRF) of our measurements, we conclude that the sum of the rates of IVR and hot CS is equivalent to a time component faster than 30 fs.

Within the paradigm of electron transfer theory developed by Marcus, the driving force for the relaxed photoinduced electron transfer between [Re(dmpe)_3_]^2+^ and anisole is exergonic and lies in the normal Marcus region (*vide infra*), and the rate of CS is controlled by the energy barrier between the Gibbs free energy surfaces of {*[Re^II^]^2+^ + A} and {[Re^I^]^+^ and A˙^+^} (Fig. 6c). Thus, during hot photoinduced electron transfer prior to IVR, [Re(dmpe)_3_]^2+^ is in a vibrationally excited state (*i.e.*, a hot state, orange circle in Fig. 6c) and the energy barrier is smaller than during CS from the vibrationally relaxed ^2^LMCT state (compare orange and purple arrow in Fig. 6c). This simplified picture helps to rationalize how a lower initial energy barrier allows a hot electron transfer process to compete with ultrafast IVR processes prior to the slower CS from the relaxed excited state. In comparison to the identification of a hot electron transfer process, our experiment is unable to quantify a hot CR process. Moreover, whereas it seems likely that hot CS is not relevant in situations where [Re(dmpe)_3_]^2+^ and anisole must first diffuse to react, our observation at high electron donor concentration highlights the very strong electronic coupling, fundamentally enabling non-adiabatic bimolecular electron transfer for close-contact donor–acceptor pairs despite the lack of chemical bonding between the donor and acceptor moieties. The spin-allowed nature of CS and CR transitions in the photocycle of [Re(dmpe)_3_]^2+^ might play a part in the ultrafast electron transfer rates observed in our study. In this context, it is worth noting that this spin argument contrasts that for typically used transition metal complexes in photoredox catalysis, because they feature photoredox-active ^3^MLCT excited states generated *via* ultrafast intersystem crossing from the initially photoexcited ^1^MLCT state. In some ways, the [Re(dmpe)_3_]^2+^/anisole system therefore resembles that of organic photosensitizers more closely due to the more significant contributions from spin-allowed processes to intrinsic deactivation of the photosensitizer and CR.^80^

For organic photosensitizers, two different CS rates have also previously been identified at high quencher concentrations in bimolecular photoinduced electron transfer reactions.^39,81^ In that work, the different rates were, however, found to be related to specific orientations between electron donor and acceptor at close contact, giving rise to different electronic couplings in so-called highly coupled and weakly coupled pairs.^81,82^ It was found that the highly-coupled pairs were undergoing fast CS but also fast CR, thus outcompeting CE. Consequently, the source for CE was the weakly coupled charge-separated pairs. In the photocycle of [Re(dmpe)_3_]^2+^, we only see an indication of one CR rate, and, as explained in the previous sections, our data suggests that there are two types of close-contact CS in our [Re(dmpe)_3_]^2+^/anisole system, originating from both a relaxed and a hot ^2^LMCT state. The photosensitizer [Re(dmpe)_3_]^2+^ contrasts the previously investigated aromatic photosensitizers in the sense that the aliphatic surface of [Re(dmpe)_3_]^2+^ is expected to lead to non-directional and weaker electron couplings between electron donor and acceptor compared to the previously investigated aromatic photosensitizers capable of directional π–π interactions. The lack of new absorption bands of [Re(dmpe)_3_]^2+^ at high anisole concentrations supports this expectation (Fig. S10).

The very fast CR in the [Re(dmpe)_3_]^2+^/anisole system (Table 1) seems to largely inhibit efficient CE. The GSB recovery does, however, not return to zero differential absorbance on the nanosecond timescale at high anisole concentrations (Fig. S37, S40, S43, and S46), indicating that some CE still takes place. The residual intensity of the GSB for photocycles containing 60–90 vol% anisole suggests that approximately 10% of the excited-state population has not undergone ground-state recovery. In other words, the CE yield is estimated to account for ∼10%. At anisole concentrations lower than 60 vol%, there is no residual absorption left at the nanosecond timescale, indicating that CE is negligible at these lower anisole concentrations. In previous work on ^2^LMCT-driven photocycles using iron(iii) photosensitizers, solvent-dependent moderate to high CE yields have in contrast been demonstrated.^26,28^ In particular, the use of halogenated solvents compared to non-halogenated solvents leads to significantly improved and high CE yields (*e.g.* 60% in dichloromethane). The origin of this result was found to be a combination of increased state-mixing due to the heavy atom effect and electrostatic repulsion between the reduced iron photosensitizer and the oxidized electron donor in the halogenated solvent.^86^ Moreover, the incorporation of a subsequent irreversible fragmentation step of the electron donor upon CS has also proved a successful strategy to outcompete CR.^40,83^ From the perspective of ultimately developing more efficient photocatalytic systems based on unconventional ^2^LMCT photosensitizers of rhenium(ii), iron(iii), or similar, it is remarkable that we observe diffusion-controlled CE at all, given the ultrafast CS and CR rates governing the photocycle of [Re(dmpe)_3_]^2+^ at anisole concentrations between 60–90 vol% (Table 1).

### Driving force dependence

As the third part of our study on the photocycle of [Re(dmpe)_3_]^2+^, we investigate how the close-contact electron transfer rates originating from the relaxed ^2^LMCT state are related to the driving force for the electron transfer by using different electron donors (Fig. 1c, Table 2). For solubility reasons, and to enable a direct comparison between the electron donor molecules, we restrict ourselves to an electron donor concentration of 5.7 M. In the second part of our study regarding anisole concentration dependence, we found that when the solvent shell consists of 50 vol% electron donor molecules or more, both the rate of close-contact CS associated with the relaxed ^2^LMCT state and the rate of CR become independent of electron donor concentration (Table 1). Given the similar size of the various investigated electron donors (Fig. 1c), a concentration of 5.7 M should therefore allow us to gain information on the intrinsic rates of close-contact CS without diffusion-controlled contributions because enough electron donors will be in the first solvent shell of [Re(dmpe)_3_]^2+^. The concentration of [Re(dmpe)_3_]^2+^ was fixed to 3.2 mM, which is three orders of magnitude lower than that of the electron donor. By using six different aromatic electron donors (Fig. 1c), we cover a range of 0.9 V in driving force for the photoinduced CS processes (Table 2). Appealingly, *N*,*N*-dimethylaniline (DMA, Δ*G*_CS_ = −1.62 V) has previously been used as an electron donor in quenching studies of [Re(dmpe)_3_]^2+^,^65^ but we unfortunately find that [Re(dmpe)_3_]^2+^ is not stable in mixtures of DMA at the high electron donor concentration of interest in the present study.

**Table 2:** Selected parameters of the investigated electron donors and corresponding time components related to photocycles driven by [Re(dmpe)_3_]^2+^ at a fixed electron donor concentration of 5.7 M in deaerated acetonitrile

Electron donor	*E* ^0^ (D˙^+^/D) [V *vs.* SCE]	Δ*G*_CS_^a^ [V]	Δ*G*_CR_^a^ [V]	[D] [vol%]	*τ*_CS,relax_ [ps]	*k*_CS,relax_ [s^−1^]	*k*_q_^65^ [M^−1^ s^−1^]	*τ*_CR_ [ps]	*τ*_CR_/*τ*_CS,relax_
Benzene (B)	+2.61 (ref. 84)	+0.03	−2.21	50	1350	7.4 × 10^8^	7.0 × 10^7^	20	0.02
Toluene (T)	+2.24 (ref. 84)	−0.34	−1.84	60	30	3.3 × 10^10^	8.0 × 10^8^	N/A^c^	N/A^c^
*o*-Xylene (*o*-X)	+2.15 (ref. 84)	−0.43	−1.75	70	1.8	5.6 × 10^11^	N/A^b^	4.5	2.5
*m*-Xylene (*m*-X)	+2.10 (ref. 84)	−0.48	−1.70	70	2.0	5.0 × 10^11^	N/A^b^	3.7	1.9
Mesitylene (M)	+2.01 (ref. 84)	−0.57	−1.61	80	1.1	9.1 × 10^11^	8.1 × 10^9^	3.9	3.5
Anisole (A)	+1.76 (ref. 85)	−0.82	−1.36	60	0.5	2.0 × 10^12^	1.3 × 10^10^	2.0	4.0

a Δ*G*_CS_ = *E*^0^(D˙^+^/D) − *E*_½_(*Re^II^/Re^I^), where *E*_½_(*Re^II^/Re^I^) = +2.58 V *vs.* SCE.^65^ Δ*G*_CR_ = *E*_½_(Re^II^/Re^I^) − *E*^0^(D˙^+^/D), where *E*_½_(Re^II^/Re^I^) = + 0.4 V *vs.* SCE.^65^ For simplicity, coulombic contributions are neglected in determination of the driving forces.

b Quencher not investigated in the previous Stern–Volmer study in ref. 65.

c It was not possible to determine this parameter.

Benzene is the hardest to oxidize of the investigated quenchers (Δ*G*_CS_ = +0.04 V *vs.* SCE), and not unexpectedly, the spectral TA features of [Re(dmpe)_3_]^2+^ do not change significantly in the presence of high benzene concentration (5.7 M in acetonitrile) relative to pure acetonitrile (Fig. S52). The decay component related to the repopulation of the ground state is, however, still shortened by one order of magnitude to 1350 ps in the presence of benzene relative to 12 ns in the absence of an electron donor (Fig. S55). The relatively slow nature of the photoinduced CS connects well with the isoenergetic nature of the electron transfer from benzene to photoexcited [Re(dmpe)_3_]^2+^. Notably, CR also quickly follows CS. Similar to the mixtures with low anisole concentrations, fitting the deviation of the kinetic traces at 800 and 435 nm enabled an estimation of the CR time component to 20 ps (Fig. S56). This means that the CR is two orders of magnitude faster than CS in the [Re(dmpe)_3_]^2+^/benzene photocycle.

**Fig. 7:**
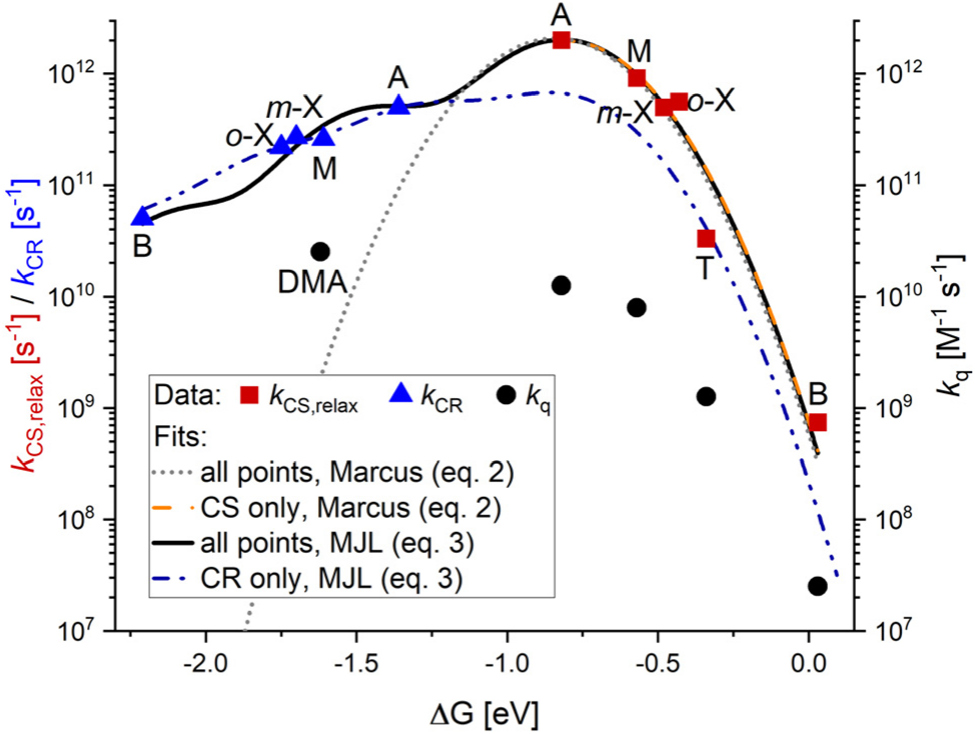
Driving force dependence of the intrinsic close-contact electron transfer rates from aromatic electron donors to [Re(dmpe)_3_]^2+^ at an electron donor concentration of 5.7 M in acetonitrile. Electron transfer rates determined in this study (Table 2) are given by red squares for *k*_CS,relax_, and blue triangles for *k*_CR_. Black circles are *k*_q_ values taken from ref. 65. B = benzene, T = toluene, *o*-X = *o*-xylene, *m*-X = *m*-xylene, M = mesitylene, A = anisole, DMA = *N*,*N*-dimethylaniline. The dotted gray curve describes the optimized fit of both *k*_CS,relax_, and *k*_CR_ rates to the semiclassical Marcus equation (eqn (2)), where *λ* = 0.814 eV and *H*_DA_ = 0.00345 eV = 28 cm^−1^. The orange dashed curve is the optimized fit of only *k*_CS,relax_ to the classical Marcus equation (eqn (2)), where *λ* = 0.814 eV and *H*_DA_ = 0.00334 eV = 27 cm^−1^. The solid black curve describes the optimized fit to Marcus–Jortner–Levich (MJL) theory (eqn (3)) for both *k*_CS,relax_, and *k*_CR_ rates, where *λ*_i_ = 0.15 eV, *λ*_o_ = 0.67 eV, *H*_DA_ = 0.00375 eV = 30 cm^−1^, and *ω*_eff_ = 0.62 eV. The blue dashed curve describes the optimized fit to Marcus–Jortner–Levich theory (eqn (3)) for *k*_CR_ rates, where *λ* = 0.82 eV (fixed), *λ*_o_ = 0.47 eV, *H*_DA_ = 0.00276 eV = 22 cm^−1^, *ω*_eff_ = 0.456 eV, and *λ*_i_ = *λ* − *λ*_o_ = 0.35 eV. Further details on optimization of the Marcus–Jortner–Levich fits can be found in the SI.

The forward electron transfer between toluene and the excited state of [Re(dmpe)_3_]^2+^ is exergonic with a free energy of Δ*G*_CS_ = −0.34 V, and the time constant of CS is 30 ps (Fig. S59). Accordingly, the photoinduced CS is 50 times faster using toluene rather than benzene as an electron donor. Despite much faster CS in toluene compared to benzene in this high concentration regime, there is no spectral indication of the charge-separated pair, and CR is thus still faster than CS. It was not possible to determine the CR rate, because the deviation of the kinetic traces at 800 and 435 nm lies within the error of the experiment; thus, fitting did not give a meaningful time component. Based on the correlation between driving force and time component for the entire investigated series of electron donors (*vide infra*, Fig. 7), the lack of CR determination for toluene could be due to the fact that the CR rate is expected to be on the same order of magnitude as the CS rate.

Employing electron donors with slightly larger driving forces for the photoinduced electron transfer than toluene, such as *o*-xylene, *m*-xylene, mesitylene, and anisole, significantly changes the excited-state dynamics at our constant high electron donor concentration of 5.7 M (Table 2). Notably, we observed an isosbestic point at 630 nm in the TA spectra on the picosecond timescale (Fig. 4, S60, S64, and S68), which is related to the build-up of the charge-separated pair, {[Re^I^]^+^ + D˙^+^}. This means that the rate of CR is slower than the rate of CS for these four electron donors. The photoinduced CS occurring from the relaxed ^2^LMCT state at close-contact has time components in the range of 0.5–2 ps for anisole, mesitylene, *m*-xylene, and *o*-xylene, and in each case, the CR is ∼3 times slower. Even though CR has slowed down relative to CS for these four quenchers, the picosecond nature of both the forward and the backward electron transfer processes prevents efficient CE, at least for the high electron donor concentration conditions investigated here. An upper limit for the CE efficiency is estimated to be ∼10% (Fig. S37, S61, S65, and S69) based on the lack of ground-state recovery on the nanosecond timescale. Unfortunately, the uncertainty connected to the CE yield determination does not allow for the establishment of a reliable trend between CE efficiency and the thermodynamic driving force for electron transfer. In addition to the lack of ground-state recovery, there are also indications of CE found in the near-IR region of our TA data, because ESA in the near-infrared region is apparent simultaneously with the observation of the isosbestic point at 630 nm associated with the build-up of the charge-separated pair {[Re^I^]^+^ + D˙^+^}. The intensity of the near-IR absorption increases with increasing anisole concentrations (Fig. 3a) and is also clearly seen for *o*-xylene (Fig. S60), *m*-xylene (Fig. S64), and mesitylene (Fig. S68). At high concentrations, aromatic radical cation monomers, D˙^+^, are known to form radical cation dimers, {D_2_}˙^+^, through association with adjacent aromatic molecules.^75,76^ Aromatic radical cation dimers typically have broad absorption bands in the near-IR region. Against this background, it is therefore expected to observe spectral features related to the radical cation dimer, {D_2_}˙^+^, as a result of CE at high electron donor concentrations. The interplay between radical cation monomers and radical cation dimers and their impact on the CE yield^86^ in the photocycle of [Re(dmpe)_3_]^2+^ is currently under further investigation.

Stern–Volmer quenching constants *k*_q_ have previously been determined in a quenching study of [Re(dmpe)_3_]^2+^ in the presence of benzene, toluene, mesitylene, and anisole through classical Stern–Volmer analysis at low quencher concentrations (black circles in Fig. 7).^65^ In the current study, we deliberately focus on a high quencher concentration to neglect diffusion-controlled processes, and by using ultrafast TA spectroscopy, we determine close-contact CS rate constants associated with photoinduced CS from the relaxed ^2^LMCT state. These relaxed intrinsic CS processes span a total of four orders of magnitude between benzene and anisole (Fig. 7 and S71, Table 2). Whereas the rate of CS is greatly influenced by the choice of electron donor (red squares in Fig. 7), the rate of CR only changes one order of magnitude between benzene and anisole (blue triangles in Fig. 7). As a result, the ratio between the time components of CR and CS (*τ*_CR_/*τ*_CS,relax_ in Table 2) changes dramatically as a function of electron donor, because the two time components are affected differently by the change in driving force. For electron donors with low thermodynamic driving force for CS, such as benzene, the rate of CR is much faster than the rate of CS. For electron donors with high thermodynamic driving forces for CS, such as mesitylene and anisole, the ratio is reversed. This ratio between the CS and CR rates determines if there is a build-up of the charge-separated pair, which is essential to promote CE.^40^ However, even in the case of anisole, where the rate of CR is four times slower than CS, CE efficiency accounts for an estimated maximum of 10% (Fig. S37). This indicates that even though there is a proper relationship between the relative rates of CS and CR in the photocycle of [Re(dmpe)_3_]^2+^, the absolute rate of CR is simply too fast and outcompetes CE dynamics, typically estimated to take place on a timescale of hundreds of picoseconds.^87–90^

Finally, we consider our CS and CR results in the framework of fundamental electron transfer theories. Because we work at a fixed electron donor concentration and the investigated electron donors have similar dielectric constants, we assume that the reorganization energy for electron transfer is the same throughout the series of electron donors. Moreover, according to Oster's rule,^91^ the polarities of the electron donor-acetonitrile mixtures will be more strongly influenced by the much more polar acetonitrile (*ε* = 36) than that of the apolar electron donors (*ε* = 2–4).^92^ The rates of CS lie in the normal Marcus regime, where the rate increases with increasing driving force. Fitting the CS rates to the classical Marcus equation (eqn (2)) gives a reorganization energy of 0.81 eV and an electronic coupling of 27 cm^−1^ (orange dashed curve in Fig. 7). Reorganization energies of ∼1 eV have also previously been determined for photocycles based on ruthenium(ii) polypyridine photosensitizers.^93–95^ In contrast to the CS rates, we find that the rate of CR in the photocycle of [Re(dmpe)_3_]^2+^ lies in the inverted Marcus regime, where the electron transfer slows down with increasing driving force (Table 2). This also means that the CR rates appear much faster than expected from semiclassical Marcus theory. In other words, the symmetric parabola describing both the CS and the CR rates predicted by classical Marcus theory does not describe our data well (gray dotted parabola in Fig. 7). This is a known feature of the inverted Marcus region, as the increased rates in the inverted region are correlated to a reduced energy barrier for the electron transfer when high vibrational levels of the product are introduced in the process description.^34,96,97^ The Marcus–Jortner–Levich theory, described by eqn (3), overcomes some of the limitations of semiclassical Marcus theory (eqn (2)) by separating the reorganization energy into two contributions, the outer-sphere reorganization energy (*λ*_o_) and the inner-sphere reorganization energy (*λ*_i_), as well as explicitly including the contribution of the higher vibrational states in the product that are coupled to the reactant (indicated by green dashed arrow in Fig. 6c). As a result, the framework of Marcus–Jortner–Levich theory predicts higher rates in the inverted region resulting in parabolas with an asymmetric shape.3
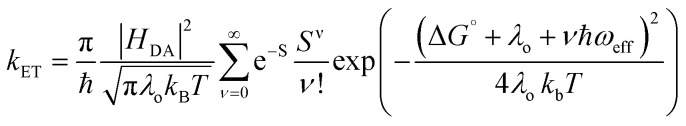
where *S* is the Huang–Rhys factor given by:4
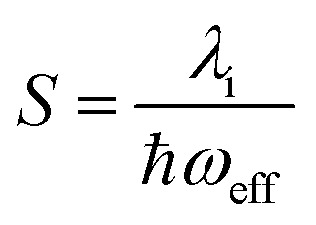


In a set of pioneering studies, Closs, Miller, and co-workers investigated intramolecular electron transfer in biphenyl-based dyads decorated with various quinone-derivatives or extended π-systems,^49,98^ where the parameters *λ*_o_, *λ*_i_, and *H*_DA_ were fitted to the experimental rates assuming a single effective vibrational mode (*ω*_eff_) with a frequency of 1500 cm^−1^. Since then, many studies have adopted this single effective mode model and employed a typical frequency of 1500 cm^−1^,^96,99–101^ corresponding to an average stretching frequency of the C–C bond in an aromatic system. Using Marcus–Jortner–Levich theory to describe our intrinsic electron transfer rates does indeed result in an improved fit compared to semiclassical Marcus theory (solid black and dotted gray curves in Fig. 7, respectively). However, a single effective mode frequency of 1500 cm^−1^ (0.187 eV) cannot describe our data, and instead, a much larger frequency is needed. An optimization of the single effective mode frequency (Fig. S73) shows that the fit is relatively insensitive to the use of a single mode frequency between 3200 cm^−1^ (0.4 eV) and 5600 cm^−1^ (0.7 eV). The need for a higher vibrational frequency is consistent with our expectations if we consider the following: (1) naturally, the driving force that corresponds to the top of the Marcus parabola is equal and opposite to the reorganization energy. The deviations from the value of the reorganization energy of the driving forces for CR are up to 5600 cm^−1^ (∼0.7 eV) or even 10500 cm^−1^ (∼1.3 eV) larger than the corresponding deviations for CS that occur with the same rates. Thus, the comparably high vibrational energy levels in the product parabola must be included in the modeling of the charge transfer rates. (2) Assuming the typical effective vibrational energy 1500 cm^−1^, 4 to 7 vibrational quanta need to be included in the model, which fails to reproduce the experimental data anyhow. Part of the reason for the failure is that, according to eqn (3), the contribution of vibrations rapidly decreases with their quantum number. Consequently, a few effective vibrations with much larger energy are required to provide a satisfactory fit of the measured electron transfer rates in the photocycle of [Re(dmpe)_3_]^2+^. The Marcus–Jortner–Levich fit is very sensitive to the value of the electronic coupling (Fig. S74) and an optimized value of 30 ± 1 cm^−1^ was obtained. The inner- and outer-sphere reorganization energies obtained by the best fit show that the outer-sphere reorganization energy (0.67 ± 0.05 eV) is much larger than the inner-sphere reorganization energy (0.15 ± 0.04 eV). This suggests that a large part of the reorganization required for the electron transfer process occurs in the environment of the excited transition metal complex. Furthermore, based on the fitted parameters, the Huang-Rhys factor is quite small (*S* ≫ 0.03, eqn (4)).

Marcus–Jortner–Levich theory describes our data reasonably well and indeed much better than semiclassical Marcus theory (Fig. 7), but clearly the Marcus–Jortner–Levich theory also has its limitations. For example, by using the Marcus–Jortner–Levich theory to fit our CS and CR rates, we assume that the coupling element and the energy contribution from reorganization processes are the same for both electron transfer processes. This is most likely not the case, as the electron transfer in CS involves a divalent rhenium complex and a neutral quencher molecule, whereas the electron transfer in CR is between a monovalent rhenium complex and a radical quencher cation. This discrepancy will presumably lead to differences in the coupling element and the reorganization energy for the CS and CR processes. It is interesting to note, though, that the separate fits for CS and CR rates only return the electronic coupling values of 27 and 22 cm^−1^, respectively (orange and blue fits in Fig. 7, respectively). Another possible limitation of the model is that only one effective vibrational frequency is used in the fit. For such a complex molecule as [Re(dmpe)_3_]^2+^, more degrees of structural variation could influence the charge transfer rates. Further studies with modified ligand structures to vary the electronic coupling and, possibly, the inner- and outer-sphere reorganization energies would be beneficial for a better understanding of the applicability of the Marcus–Jortner–Levich theory to describe variations of electron transfer rates over a wide range of driving forces.

In summary, we show that by using a high electron donor concentration of 5.7 M, it is possible to determine the intrinsic close-contact rates of CS and CR in photocycles of [Re(dmpe)_3_]^2+^ covering a large span of driving forces. We show that by changing the driving force by 0.5 V for CS, the rate of CS is changed by four orders of magnitude. This contrasts with the behavior for CR, where a change of 0.5 V in driving force only changes the electron transfer rate by one order of magnitude. The decreased sensitivity to a change in driving force for the CR compared to CS is rooted in the fact that the rates of CR lie in the inverted Marcus region, whereas the rates of CS follow normal Marcus behavior. Our insights into the photocycle of [Re(dmpe)_3_]^2+^ demonstrate the ability to leverage the counteracting trends in driving force dependence of CS and CR to promote the build-up of charge-separated species as a first step towards photoredox applications but simultaneously highlights the necessity to also slow down the absolute rate of CR to obtain large CE yields in bimolecular photoinduced electron transfer processes.

## Conclusion

Diffusion-controlled processes typically overshadow the intrinsic electron transfer rates in bimolecular photocycles, but by employing high electron donor concentrations, the donor–acceptor pair can be set in close contact and the electron transfer rates that reflect the electronic interactions between donor and acceptor, rather than diffusive motions, can be investigated. Using this strategy, we quantify both intrinsic CS and CR processes over a broad range of electron donor concentrations in the photocycle of [Re(dmpe)_3_]^2+^ using TA spectroscopy at ultrafast timescales. In this way, our study provides unique insights on the dynamics of the charge-separated pair because we directly monitor the generation and depletion of {[Re^I^]^+^ + D˙^+^} (Fig. 1a). Besides diffusion-controlled and close-contact CS occurring from the relaxed ^2^LMCT state of [Re(dmpe)_3_]^2+^, our analysis on the ultrafast timescales (<200 fs) suggests that a dominant part of the excited-state population can undergo electron transfer from vibrationally higher lying ^2^LMCT states. This so-called hot electron transfer is observed when the electron donor concentration is increased to a point where multiple electron donors are statistically expected to be in close contact with each [Re(dmpe)_3_]^2+^ complex. This also means that the hot electron transfer occurs on a timescale of sub-30 fs that is competitive with IVR within the ^2^LMCT manifold. While this ultrafast CS component would not be expected to persist in situations where the electron donor and [Re(dmpe)_3_]^2+^ first must diffuse to react, our observation highlights the very strong electronic coupling fundamentally enabling non-adiabatic bimolecular electron transfer for close-contact donor–acceptor pairs, despite the lack of chemical bonding between the donor and acceptor moieties. The spin-allowed nature of key transitions (including direct deactivation and the bimolecular CR in our photosensitizer/electron donor systems) in the photocycle driven from the ^2^LMCT state of the [Re(dmpe)_3_]^2+^ complex is likely to play a part in the ultrafast electron transfer rates observed in our study.

As a significant additional step, we were also able to characterize intrinsic close-contact CS and CR rates over a wide range of thermodynamic driving forces by leveraging the very strong photo-oxidation capability of our prototype photosensitizer [Re(dmpe)_3_]^2+^ together with a range of aromatic electron donors (Fig. 1c). These results demonstrate the ability to vary the thermodynamic driving forces to influence the intrinsic bimolecular electron transfer dynamics significantly. In particular, varying the driving force for CS and CR modified the CS and CR rates substantially (Fig. 7). As a result, the CS/CR ratio could be varied from a case where CR outpaced CS to a scenario where CS exceeds CR (*τ*_CR_/*τ*_CS,relax_ in Table 2) by exploiting the opposite trends in electron transfer rates, *i.e.*, CS following the normal Marcus region and CR the inverted Marcus region. Whereas it is naturally important to control the relative ratio of the CS and CR rates for building up a significant population of the charge-separated pair, the fast nature of the absolute rates in the photocycle of [Re(dmpe)_3_]^2+^ results in only minor CE (upper CE limit estimated to 10%). This demonstrates that CE cannot compete with the ultrafast dynamics governing the depletion of the charge-separated pair in the high concentration regime employed to separately determine intrinsic CS and CR rates. These findings also suggest that, at least for all the physical conditions that we have investigated here, the basic CE model focusing on diffusive separation of the charge-separated pair initially contained within a solvent cage is effectively never in play, as the ultrafast electron transfer rates clearly outcompete diffusional CE rates. This outcome is consistent with a closely-coupled charge-separated pair surrounded by a solvent cage. In other words, our study suggests that if the charge-separated pairs are too closely coupled, the charge-separated pairs are, in fact, prevented from escaping the solvent cage by the ultrafast bimolecular electron transfer kinetics, as diffusive CE dynamics taking place on slower timescales cannot compete. Means to control both relative and absolute electron transfer rates in photocycles driven by ^2^LMCT excited states are therefore required. The fact that the CS and CR rates gradually decrease as the electron donor concentrations are lowered below the saturation limit (Table 1) evidently plays a key role in making diffusive CE dynamics competitive with spatial separation of the charge-separated pair. Rather counter-intuitively, it also indicates that what has typically been described as CE efficiencies in diffusion-limited dynamics needs, in fact, to be described as a more complex interplay between intrinsic electron transfer rates and diffusive dynamics of donor–acceptor pairs undergoing more loosely-coupled encounters^81^ that are never actually fully enclosed inside a solvent cage.

Ultimately, the fast CR behavior observed for [Re(dmpe)_3_]^2+^ means that it does not perform very well as a photoredox catalyst (CE < 10%) despite its high excited-state reduction potential and favorable thermodynamic parameter for charge separation. Ligand design strategies could play a useful role in controlling the relative CS and CR rates by ideally slowing down the rate of CR due to a weaker electronic coupling between the reduced photosensitizer and oxidized quencher, and thereby favoring CE. In addition, previous studies using iron(iii) photosensitizers in ^2^LMCT-driven photoredox catalysis have shown that the solvent polarity plays a significant role in obtaining high CE yields.^26,27^ Whereas solvent modification clearly will affect solvent dynamics and therefore CE dynamics, it would, in contrast, be expected to have a relatively minor influence on the intrinsic close-contact biomolecular electron transfer rates in focus here. To add to the complexity in the discussion of CE processes, it should be realized that we do nevertheless observe some CE in cases where ultrafast electron transfer kinetics of charge-separated pairs clearly outpace diffusional dynamics required for the traditional CE model. Given the current interest in photoredox catalysis using novel photosensitizer strategies,^27,83,102–110^ this study on a ^2^LMCT driven photocycle highlights the need for a more comprehensive and refined understanding of the complex bimolecular dynamics, commonly referred to as CE dynamics; as shown here, CE dynamics involve a highly complex competition between bimolecular electron transfer processes on one hand, and physical diffusional encounter and separation processes on the other hand, and in this context, the driving force between electron donor and electron acceptor cannot alone be used as a guiding principle for the photocatalytic behavior.

## Author contributions

Conceptualization: C. W., A. Y., P. P. Formal analysis: C. W., N. C., M. K., P. C., A. Y. Funding acquisition: C. W., A. Y., P. P. Investigation: C. W., N. C., M. K., P. C., A. Y. Supervision – C. W., N. C., A. Y., P. P. Visualization: C. W., M. K. Writing – original draft: C. W., A. Y., P. P. Writing – review and editing: N. C., M. K., P. C.

## Conflicts of interest

There are no conflicts to declare.

## Supplementary Material

SC-OLF-D5SC03839A-s001

## Data Availability

The data supporting this article has been included as part of the supplementary information (SI). Supplementary information: synthetic protocols and characterization data, description of equipment and methods as well as supplementary spectroscopic data. See DOI: https://doi.org/10.1039/d5sc03839a.
