# Supplementary material for: Captivating bimolecular photoredox dynamics of a ligand-to-metal charge transfer complex
Source: Chem Sci. 2025 Oct 20;16(46):21975–90. doi: 10.1039/d5sc03839a (PMC12536450; doi:10.1039/d5sc03839a)
Supplement: SC-016-D5SC03839A-s001 [file SC-016-D5SC03839A-s001.pdf]

Supplementary Information (SI) for Chemical Science.
This journal is © The Royal Society of Chemistry 2025

Supplementary Information to accompany:

Captivating Bimolecular Photoredox Dynamics of a Ligand-to-Metal Charge Transfer Complex

Christina Wegeberg,[1,#,*] Neus A. Calvet,[1] Mila Krafft,[1] Pavel Chábera,[1,¤] Arkady Yartsev[1],* Petter Persson,[2,*]

[1]Division of Chemical Physics, Department of Chemistry, Lund University, 22100 Lund, Sweden.

[2]Division of Computational Chemistry, Department of Chemistry, Lund University, 22100 Lund, Sweden.

[#]Current address: Department of Physics, Chemistry and Pharmacy, University of Southern Denmark, 5230 Odense M, Denmark.

[¤]Current address: Renewable and Sustainable Energy Research Center, Technology Innovation Institute (TII), P.O. box 9639, Masdar City, Abu Dhabi, United Arab Emirates

Table of Contents

1. **Materials and Preparation** S2
2. **Instrumentation and Methods** S2
3. **Synthesis and Characterization of $[Re(dmpe)_3][BArF_{24}]_2$** S6
4. **Steady-state Absorption Spectroscopy** S10
5. **Poisson Distribution** S12
6. **Excited State Dynamics of $[Re(dmpe)_3]^{2+}$** S15
7. **Excited State Dynamics – Anisole Dependence** S18
8. **Excited State Dynamics – Driving Force Dependence** S41
9. **Analysis using Marcus-Jortner-Levich theory** S58
10. **Photostability studies** S60
11. **Absorption spectrum of the anisole radical cations** S61
12. **References** S62

1. Materials and Preparation

$[Re(O)_2(PPh_3)_2I]$ and tritylium tetrakis[3,5-bis(trifluoromethyl)-phenyl]borate ($[Ph_3C][BArF_{24}]$) were synthesized in accordance with previous protocols.[1, 2] The ligand 1,2-bis(dimethylphosphino)ethane (dmpe) and solvents were purchased from commercial suppliers and readily used without further purification. The synthesis was carried out under N_2 atmospheres using standard Schlenk line techniques.

A fixed concentration of 3.5 mM of $[Re(dmpe)_3]^{2+}$ was used in all photophysical measurements. Samples for optical spectroscopy were prepared by dissolving $[Re(dmpe)_3][BArF_{24}]_2$ in anhydrous acetonitrile and subsequently quencher was added to reach the appropriate quencher concentration. The sample solutions were filtered (pore size 0.22 μm) before loading to 1 mm optical path length cuvettes (Hellma – Optical Special Glass) sealed with a septum. The solutions were purged with N_2 for 5 minutes prior measurements. Optical measurements were performed at room temperature. The photostability of the samples was monitored by measuring the steady-state absorption spectra before and after transient absorption (TA) experiments.

2. Instrumentation and Methods

Nuclear magnetic resonance (NMR) spectra (^1H, ^{13}C, ^{31}P, DEPT-135) were recorded on a Bruker Avance II 400 MHz spectrometer. Chemical shifts (δ) are denoted relative to the residual solvent peak of CD_2Cl_2 (δ_H = 5.32 ppm; δ_C = 53.84 ppm). The coupling constants (J) of ^1H-^1H couplings are given in *Hz* and these are described by the following abbreviations: s (singlet), t (triplet) and m (multiplet). Elementary analysis was performed on a FlesgEA 1112 instrument. High-resolution electrospray ionization (ESI) mass spectra were recorded in high-resolution positive-ionization mode with a Bruker microTOF-QII mass spectrometer. Electron paramagnetic resonance (EPR) spectra (X-band) were recorded on a Bruker EMX Plus CW spectrometer and eview4wr was used for data analysis.[3] Cyclic voltammetry was recorded using a EmStat3 potentiostat from PalmSens, a glassy carbon disk working electrode, a platinum wire counter electrode, and Ag/Ag^+ (silver wire in 5 mM $AgNO_3$/0.1 M Bu_4NPF_6 in acetonitrile) isolated by a frit used as a reference electrode. Internal potential calibration occurred by addition of small amounts of ferrocene. The solvent was dry acetonitrile containing 0.1 M Bu_4NPF_6 as the supporting electrolyte. The solutions for the electrochemical experiments were purged with solvent-saturated nitrogen and kept under an inert atmosphere throughout the measurements. The scan rate was 100 mV/s. The spectroelectrochemical generated reference spectrum of the anisole radical cation ion was obtained by applying a constant potential of +1.9 V vs. SCE for 10 min to a solution of anisole in acetronitrile. Steady-state absorption spectra were recorded on an Agilent 8453 spectrophotometer. Photostability studies were carried out with a 470 nm fiber-coupled LED (M470F4) from Thorlabs. Steady-state emission spectra (i.e. spontaneous emission) were recorded on a Horiba Fluorolog-3 fluorimeter. To account for the contribution from stimulated emission in recorded transient absorption spectra, we convert the measured spontaneous emission spectrum to that of the stimulated emission by a two-step

procedure. First, the spontaneous emission was converted from wavelength (nm) to wavenumbers (cm^{-1}) scale using eq. S1. Then the stimulated emission spectrum was obtained from the spontaneous emission spectrum by accounting for the differences in the Einstein coefficients, see eq. S2.

$$I(\bar{\nu})_{spon} = \lambda^2 \cdot I(\lambda)_{spon} \qquad \text{(eq. S1)}$$

The Einstein coefficient of spontaneous emission $\boldsymbol{A_{21}}$ is coupled to the stimulated emission coefficient $\boldsymbol{B_{21}}$, as:

$$\boldsymbol{A_{21}} = \frac{8\pi h \nu^3}{c^3} \cdot \boldsymbol{B_{21}} \qquad \text{(eq. S2)}$$

Broadband transient absorption (TA) spectra were obtained by using an in-house build setup based on a Solstice Ace (Spectra Physics) laser amplifier system that produces ~80 fs pulses at a central wavelength of 796 nm at 4 kHz repetition rate. The laser output is split into two parts that are used to pump two collinear optical parametric amplifiers (TOPAS-C, Light Conversion). The first TOPAS is used to generate the pump beam (530 or 540 nm). The second TOPAS is used to generate a NIR beam (1350 nm) that is focused onto a 5 mm CaF_2 crystal to generate a supercontinuum probe beam. The delay between pump and probe beams is introduced by a computer-controlled delay stage (Aerotech) placed in the probe beam's path. After supercontinuum generation the probe pulses are split into two parts: the former overlapping with the pump pulse in the sample volume and the latter serving as a reference. The probe and the reference beams are then relayed onto the entrance aperture of a prism spectrograph and dispersed onto a double photo-diode array, each holding 512 elements (Pascher Instruments). The power of excitation pulses for samples containing anisole was set to 7 mW and for all other samples it was set to 3.5 mW, corresponding to 2.5 x 10^{15} and 1.2 x 10^{15} photons/pulse/cm^2, respectively. The diameters of the pump and the probe were ~700 µm and 150 µm, respectively. The polarization between pump and probe beams was set to either 90° (perpendicular) or 54.7° (magic angle) by placing a Berek compensator in the pump beam. Time-resolution of the setup after dispersion correction is ≤200 fs. Background- and chirp-correction of the TA data as well as global fit analysis were performed in software DA Fit 3 from Pascher instruments. Kinetic traces were averaged over a range of wavelengths as indicated by the figure inserts to improve the signal-to-noise ratio. The averaged spectral range is, however, still narrow (maximum ± 10 nm).

Femtosecond transient absorption (fs-TA) spectroscopy kinetics are obtained with an in-house built setup with two different laser sources.
Measurements with 590 nm probe: The probe light at 590 nm is produced by a set-up pumped by a Clark MXR CPA 2001 laser that provides 100 fs pulses with 1 mJ energy centered at 775 nm at 1 kHz repetition rate. A portion (60% of power) of this output is sent into a self-built nonlinear optical parametric amplifier (NOPA)

with double channels for the generation of a pump and a probe beam. The excitation pump with a central wavelength of 530 nm and a bandwidth of 25 nm is obtained in a double-stage amplification NOPA scheme and further compressed with a prism compressor into 23 fs pulses. The probe centered at 590 nm with a bandwidth of 35 nm is achieved by a single stage NOPA and sent into another prism compressor to achieve a pulse duration of 27 fs.
Measurements with 800 nm probe: The 800 nm probe with a bandwidth of 40 nm and a pulse duration of 25 fs, and its corresponding excitation pump at a 530 nm wavelength (bandwidth: 22 nm, pulse duration: 26 fs) are produced by a Pharos (Light Conversion) laser source, set to 1 kHz repetition rate and modulated by two commercially available NOPAs (Orpheus-N, Light Conversion), that have in-built compressors.

The delay between pump and probe pulses in the fs-TA spectroscopy setup is achieved by sending the probe beams into a combination of two computer-controlled delay lines: a shorter 500-ps to resolve fast decay components and a longer 12-ns (Aerotech) to capture longer-lived dynamics. Before the sample, the probe beam is split into a reference probe beam and a sample probe beam, the latter overlapping with the pump beam at the sample position. At the sample position the pulse excitation energy of the pump beam is 0.4 μJ. The beam width (D4σ) of the pump and probe beams is 320 μm and 140 μm, respectively, for measurements at 590 nm probe and 300 μm and 100 μm, respectively, for measurements at 800 nm probe. The spot size is achieved by soft focusing: The pump is focused with a lens of 1 m focal length, and the sample probe is sent into a spherical mirror with a radius of curvature of 0.5 m. The mutual polarization of pump and probe beams was set to 90° (perpendicular polarization).

The pulse duration of the pump and probe beams from both laser sources are characterized with cross- and autocorrelation at the sample position. The detection system comprises two single silicon photodetectors (Pascher Instruments) to collect the sample and reference probe beams. A third photodetector is used to detect a part of the chopped pump (Chopper: Thorlabs, frequency 0.5 kHz) for the measuring software to sort out pump and non-pumped events.

We learn about the excited state dynamics of $[Re(dmpe)_3]^{2+}$ by collecting TA data in two ways, broadband TA and fs-TA, as detailed above. The two approaches have both their advantages and disadvantages. For the broadband TA, we resolve spectra from ~350 nm to ~1150 nm with a spectral bandwidth of ~1 nm in the UV to green region and ~5 nm in the red to nearIR region. For the broadband TA setup it is, however, not possible to time resolve dynamics faster than 200 fs due to solvent and cuvette artefacts. This means that the broadband TA is a useful method for getting information on spectral features, but not on the ultrafast dynamics. In contrast, in the fs-TA the entire probe beams are detected implying that no spectral resolution apart from the spectral shape of the probe pulses is available. This approach, however, enables instrument response function of ~30 fs. In this way, we can use the fs-TA approach to gain information on the ultrafast timescales, which is

not possible to time-resolve using the broadband TA method. The different nature of broadband TA and fs-TA also means that a kinetic trace centered at the same wavelength is not measured over the same spectral region in the two methods. For instance, following the kinetic trace at 590 nm with broadband TA means 590 ±1 nm, whereas for fs-TA it is more like 590 ± 40 nm. For this reason, the resulting kinetic traces presented for the same probe wavelength will not necessarily be the same for the two TA methods, because there will be different spectral contributions to the absorption kinetics measured by the two methods. This effect will especially be pronounced for narrow spectral features such as the SE and GSB in the TA spectra of $[Re(dmpe)_3]^{2+}$ or where the differential absorption changes behavior from positive to negative or *vice versa*.

Generation of the ESA spectra

In general, a TA spectrum represents the sum of contributions from GSBs, ESAs, and SE. For $[Re(dmpe)_3]^{2+}$ it is informative to deduce the ”pure” ESA spectrum to gain further insight into photoinduced electron transfer processes. This can be done, because both the ground state absorption (GSA) spectrum and the TA spectra have unique spectral features in the region between 450 and 550 nm, which allows for a proper scaling of the GSA spectrum relative to the TA spectrum and hereby for subsequent subtraction of the consequent GSB signal. In this way, a spectrum only including contributions from the ESA and SE was calculated (orange dotted spectrum in Figure 2d). It is worth mentioning that subtraction of the scaled GSA signal imposes a scaling factor for the excited state concentration that can be calculated from the amplitude of the GSA and the ground state extinction coefficient. In other words, the y-axis of the TA spectrum can be converted from differential optical density (ΔOD) to extinction units. The shape of the SE spectrum was calculated from the steady-state emission spectrum (i.e., spontaneous emission, see eq. S1 and S2) and subsequently scaled to the TA spectra. As a result, the SE can be removed from the TA spectrum and a purified ESA spectrum (blue spectrum in Figure 2d) is obtained in extinction units.

3. Synthesis and Characterization of [Re(dmpe)$_3$][BArF$_{24}$]$_2$

[Re(dmpe)$_3$][BArF$_{24}$]$_2$ was synthesized following the previously published procedure for [Re(dmpe)$_3$][B(C_6H_5)$_4$]$_2$.[4]

[Re(dmpe)$_3$][BArF$_{24}$]

[Re(O)$_2$(PPh_3)$_2$I] (360 mg, 0.41 mmol, 1 eq.) and dmpe (620 µL, 3.7 mmol, 9 eq.) were dissolved in dry 1,2-dichlorobenzene and heated under N_2 at 150 °C for 3 hours, during which time the reaction mixture changed color from red, to yellow, and finally to colorless alongside precipitation of a colorless solid. The reaction mixture was cooled to room temperature, and the solid was removed by filtration. Diethyl ether (20 mL) was added to the filtrate, whereupon [Re(dmpe)$_3$]I precipitated as a white solid. [Re(dmpe)$_3$]I was isolated by vacuum filtration in nearly quantitative yield (316 mg). The iodide complex [Re(dmpe)$_3$]I and Na[BArF$_{24}$] (404 mg, 0.46 mmol, 1.1 eq.) were suspended in dry dichloromethane (8 mL) and stirred at room temperature for 5 hours, whereupon NaI precipitated as a white solid. The suspension was filtered, and methanol (40 mL) was added to the filtrate whereupon [Re(dmpe)$_3$][BArF$_{24}$] precipitated as a white solid. The volume was reduced to approximately half to induce further precipitation. [Re(dmpe)$_3$][BArF$_{24}$] was isolated by vacuum filtration (405 mg, 65% yield). ^{1}H NMR (400 MHz, CD_2Cl_2) δ 7.73 (t, *J* = 5.0, Hz, 8H), 7.57 (s, 4H), 1.71 – 1.59 (m, 6H), 1.56 (s, 18H), 1.45 (s, 18H), 1.40 (m, 6H) ppm. ^{13}C NMR (101 MHz, CD_2Cl_2) δ 162.19, 135.22, 125.02, 117.89, 34.61 (CH_2), 24.98 (CH_3), 23.15 (CH_3) ppm.^{31}P{^{1}H} NMR (162 MHz, CD_2Cl_2) δ 1.8 (br. s, $\nu_{½}$ = 800 Hz) ppm. HRMS (ESI, positive ions): *m/z* 637.1727 (calc. for [Re(dmpe)$_3$]$^+$ 637.1734). Elemental analysis for [Re(dmpe)$_3$][BArF$_{24}$](H_2O)$_2$(CH_3OH)$_2$ ($C_{52}H_{72}BF_{24}O_4P_6Re$) found (calcd.): C, 38.72 (39.04); H, 4.65 (4.54) %. $E_{½}$ (Re^{II}/Re^{I}) = -0.1 V vs $Fc^{+/0}$.

[Re(dmpe)$_3$][BArF$_{24}$]$_2$

[Re(dmpe)$_3$][BArF$_{24}$] (240 mg, 0.16 mmol, 1 eq.) and [CPh_3][BArF$_{24}$] (204 mg, 0.18 mmol, 1.15 eq.) were dissolved in dry acetonitrile (10 mL). The reaction mixture was refluxed for 4 hours under N_2, during which time the reaction mixture changed color from yellow to pink alongside precipitation of a white solid. The reaction mixture was cooled to room temperature, and the white solid was removed by filtration. The filtrate was concentrated in vacuum to afford a pink solid. Diethyl ether (5 ml) was added to pink solid, and trituration yielded [Re(dmpe)$_3$][BArF$_{24}$]$_2$ as a fine pink powder, which was isolated by vacuum filtration (307 mg, 81% yield). HRMS (ESI, positive ions): *m/z* 637.1682 (calc. for [Re(dmpe)$_3$]$^{2+}$ 637.1728). Elemental analysis for [Re(dmpe)$_3$][BArF$_{24}$]$_2$(H_2O)$_2$(CH_3CN)$_2$ ($C_{86}H_{82}B_2F_{48}N_2O_2P_6Re$) found (calcd.): C, 41.76 (41.63); H, 3.70 (3.33) %. $E_{½}$ (Re^{III}/Re^{II}) = +0.8 vs $Fc^{+/0}$. *g*-value: 2.20 (*S* = ½).

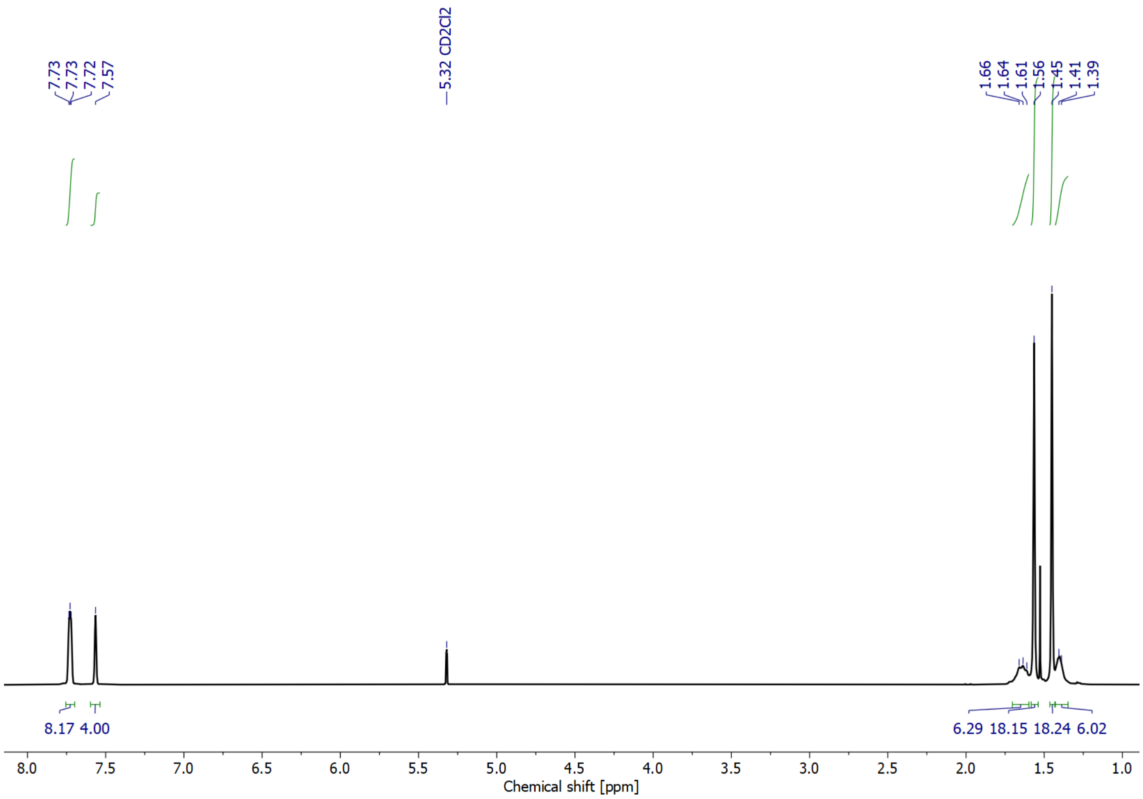

Figure S1. ^{1}H NMR spectrum of $[Re(dmpe)_3][BArF_{24}]$ in CD_2Cl_2 (400 MHz, 298 K).

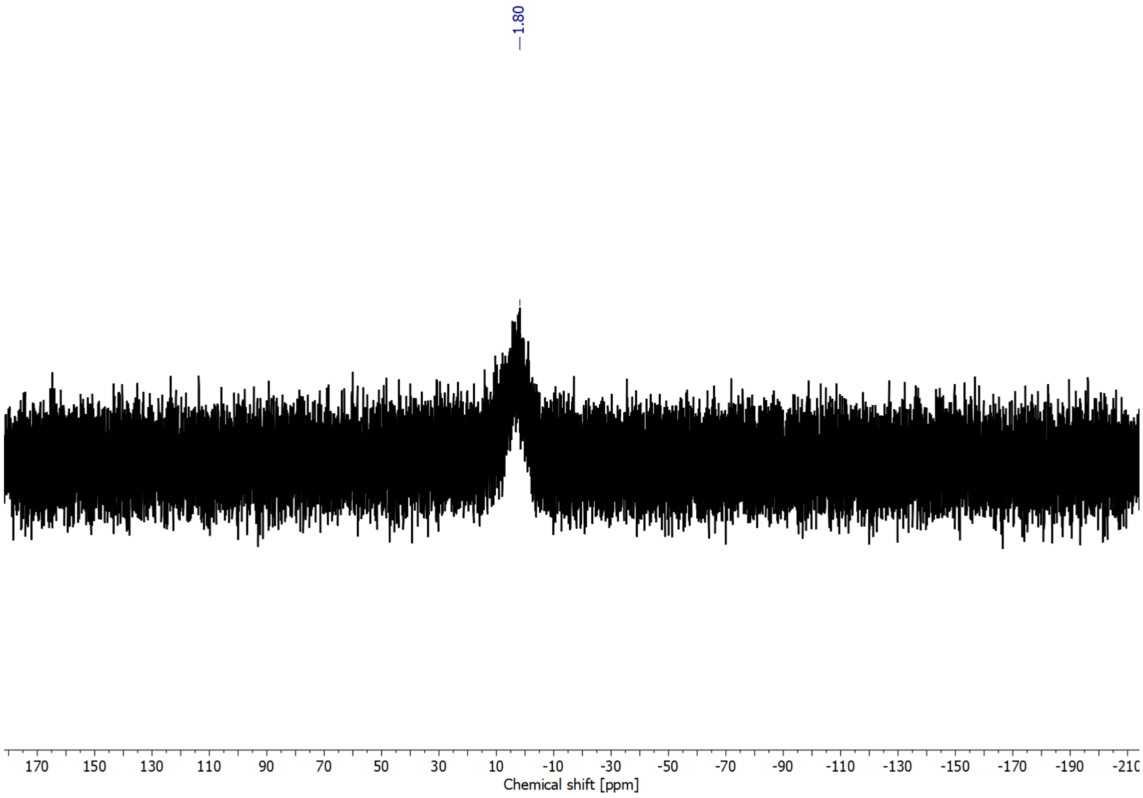

Figure S2. ^{31}P NMR spectrum of $[Re(dmpe)_3][BArF_{24}]$ in CD_2Cl_2 (162 MHz, 298 K).

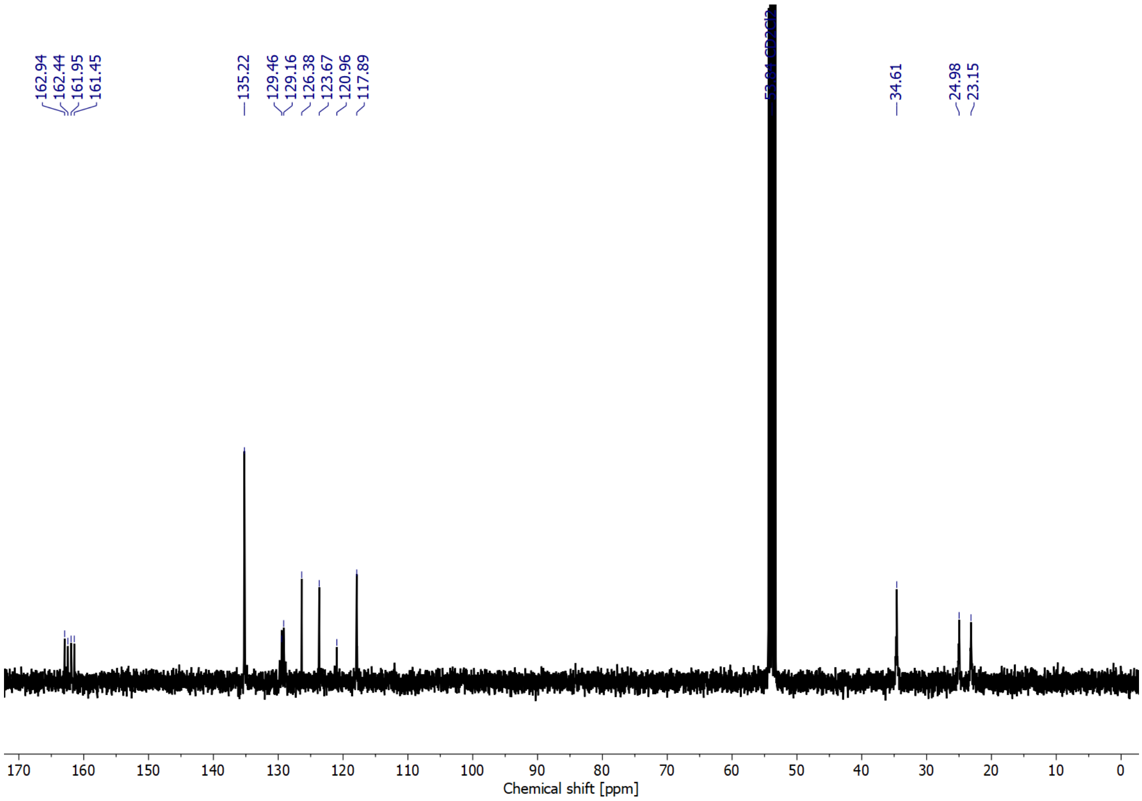

Figure S3. $^{13}C\{^{1}H\}$ NMR spectrum of $[Re(dmpe)_3][BArF_{24}]$ in CD_2Cl_2 (101 MHz, 298 K).

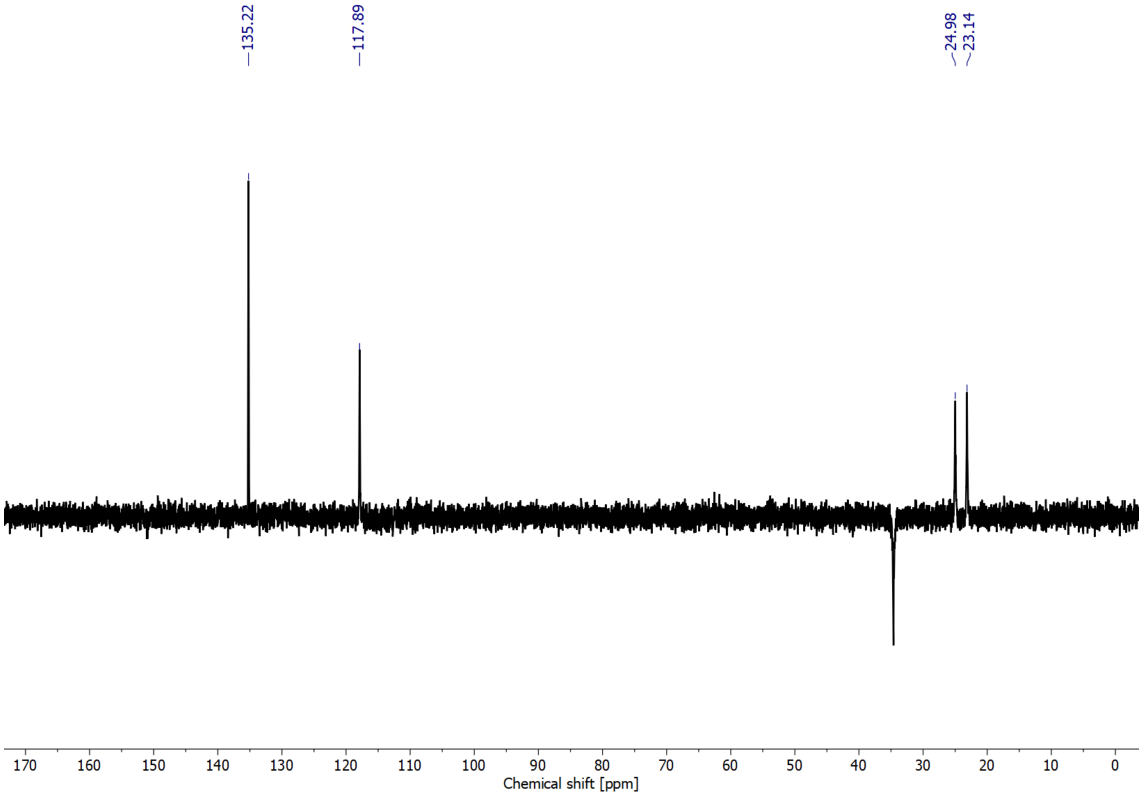

Figure S4. DEPT-135 NMR spectrum of $[Re(dmpe)_3][BArF_{24}]$ in CD_2Cl_2 (101 MHz, 298 K).

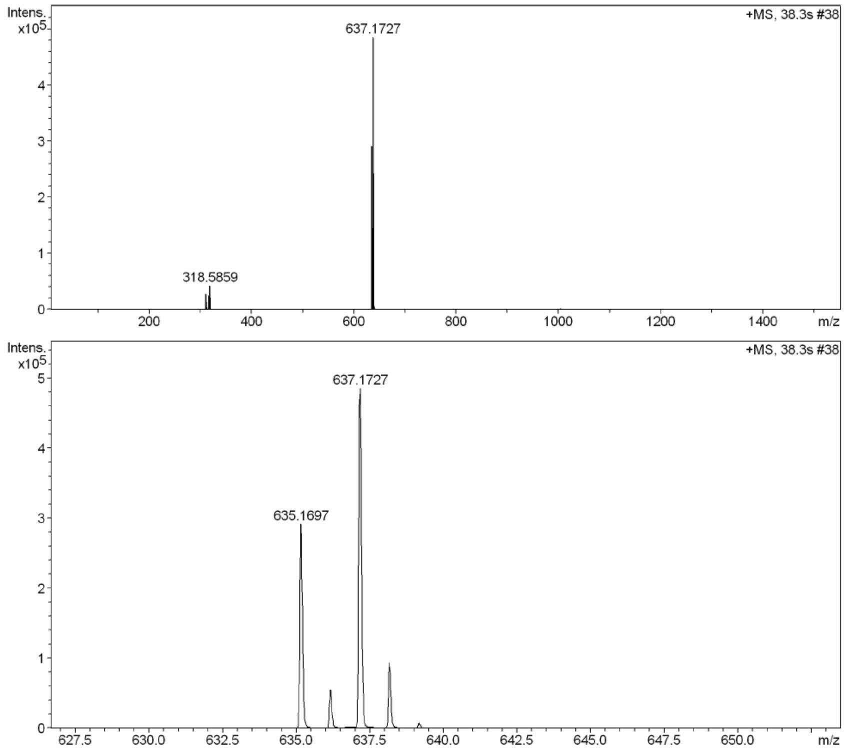

Figure S5. High resolution ESI mass spectrometry spectrum of $[Re(dmpe)_3]^+$ (ESI, positive ions).

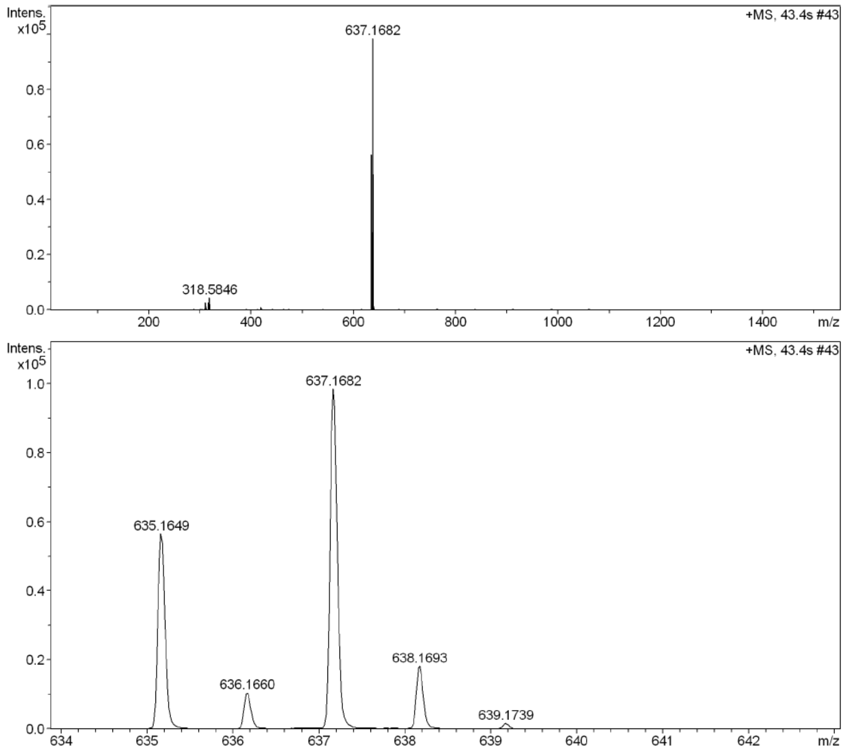

Figure S6. High resolution ESI mass spectrometry spectrum of $[Re(dmpe)_3]^{2+}$ (ESI, positive ions).

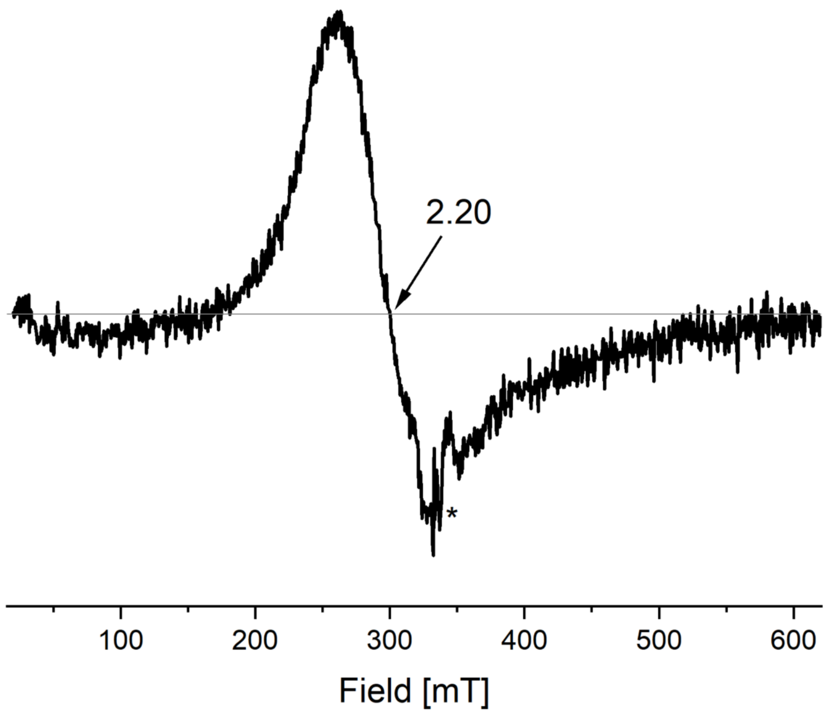

Figure S7. X-band EPR spectrum of $[Re(dmpe)_3][BArF_{24}]_2$ in Acetonitrile (1 mM) at 100 K. Frequency: 9.31 GHz. Modulation: 10 dB. * = Artefact from cavity. The *g*-value is 2.20.

4. Steady-state Absorption Spectroscopy

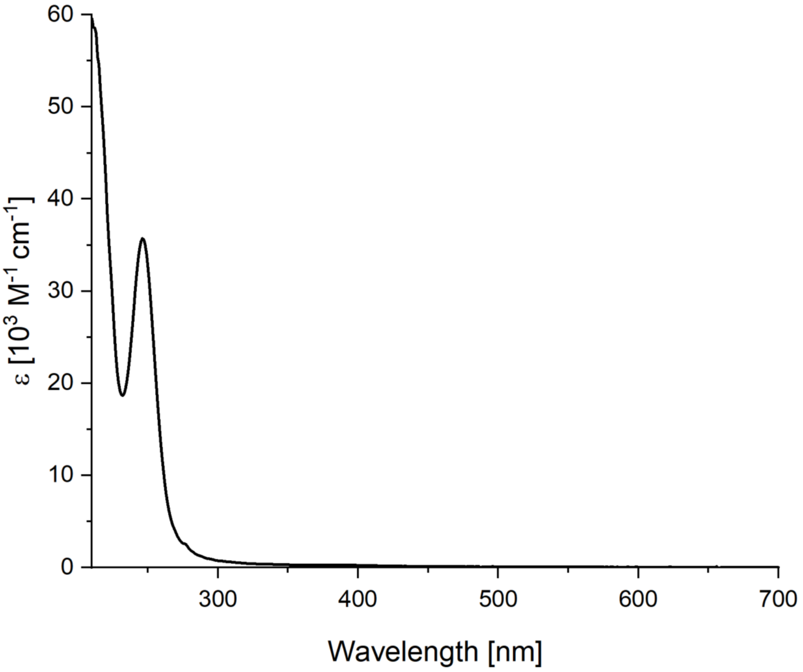

Figure S8. Absorbance spectrum of $[Re(dmpe)_3]^+$ in acetonitrile.

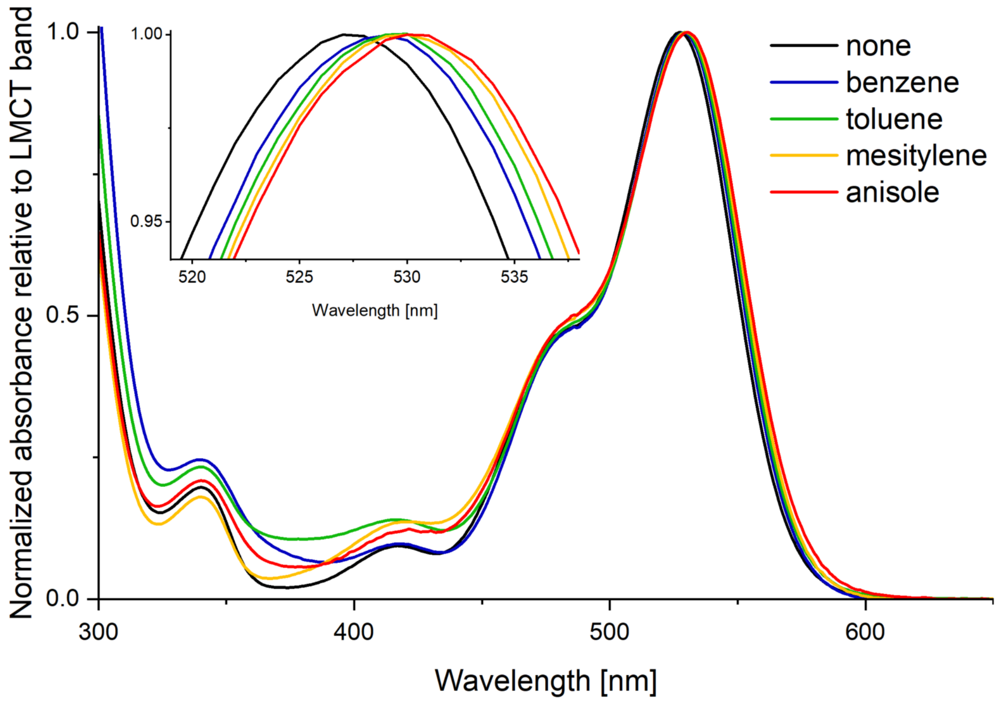

Figure S9. Normalized absorption spectra of $[Re(dmpe)_3]^{2+}$ in deaerated acetonitrile (black) and mixtures of acetonitrile and quencher molecules, where the color coding of the quenchers is noted in the insert. The quencher concentration is 5.7 M. The LMCT band redshifts as a function of increased polarity of the quencher.

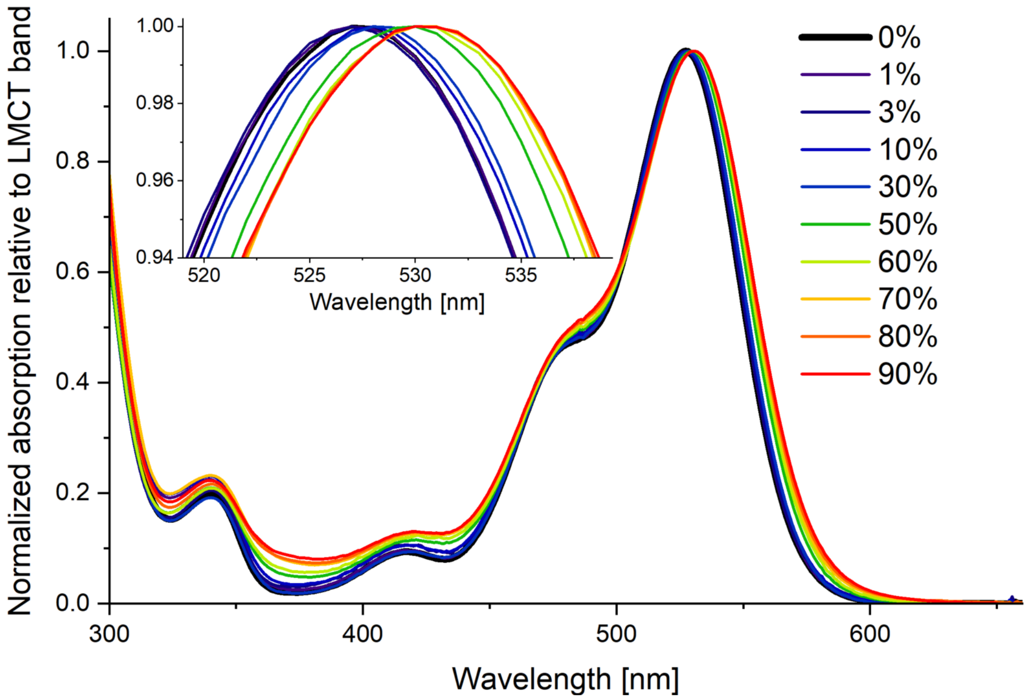

Figure S10. Normalized absorption spectra of $[Re(dmpe)_3]^{2+}$ in deaerated mixtures of acetonitrile and anisole. The concentration of anisole in acetonitrile (vol%) is seen in the insert. The LMCT band redshifts as a function of increased anisole concentration.

5. Poisson Distribution

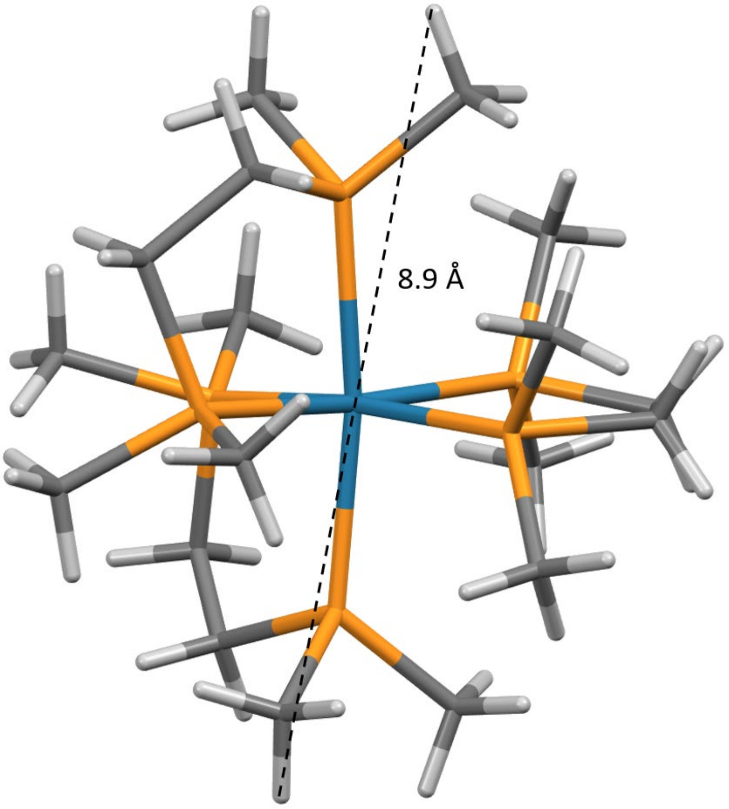

Figure S11. Crystal structure of the cation in $[Re(dmpe)_3][B(C_6H_5)_4]_2$. Cambridge crystallographic data center (CCDC) refcode: JUWJUI. Color coding: Rhenium (blue), phosphine (orange), carbon (dark gray) and hydrogen (pale gray) atoms.

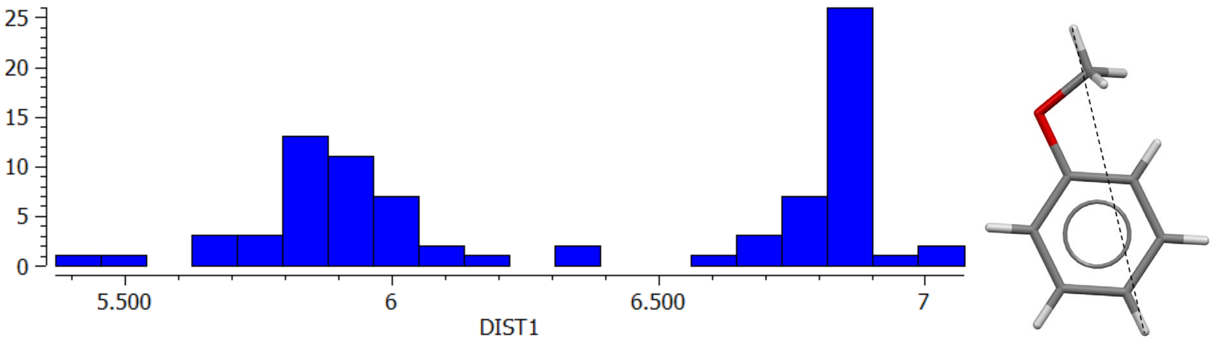

Figure S12. Distance between hydrogen atoms in the methyl group and the hydrogen atom in para position in the anisole molecule found in the CCDC database (version 2022). Based on the 65 crystal structures, the longest most typical distance is 6.9 Å. Color coding: Oxygen (red), carbon (dark gray) and hydrogen (pale gray) atoms.

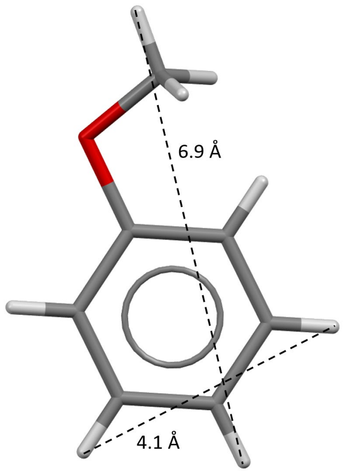

Figure S13. Typical dimensions of anisole.

Number of anisole molecules in the solvent shell of $[Re(dmpe)_3]^{2+}$

To a first approximation $[Re(dmpe)_3]^{2+}$ is a sphere and the diameter of $[Re(dmpe)_3]^{2+}$ is 8.9 Å based on crystallographic data (Figure S11). The dimensions of an anisole molecule are approximately 6.9 Å x 4.1 Å (Figure S13). For simplicity, we treat the anisole molecule as a circle with an averaged diameter of 5.6 Å, resulting in a flat circle area of ~25 Å^2. The average center-to-center distance of the $[Re(dmpe)_3]^{2+}$-anisole pair is 7.3 Å (Figure S14), which yields a spherical surface area of ~660 Å^2.

Based on these numbers, it is now possible to estimate a theoretical maximum number of anisole neighbors per $[Re(dmpe)_3]^{2+}$ to 26 anisole molecules (660 Å^2/ 25 Å^2= 26). It is, however, important to realize that the maximum packing density of circles on a spherical surface area is far from perfect. In the following Poisson calculations (Table S1), we therefore use the rough estimate of maximum 20 anisole neighbors per $[Re(dmpe)_3]^{2+}$ (N_{max}) to take imperfect packing into account in addition to allow for thermal wiggling motions.

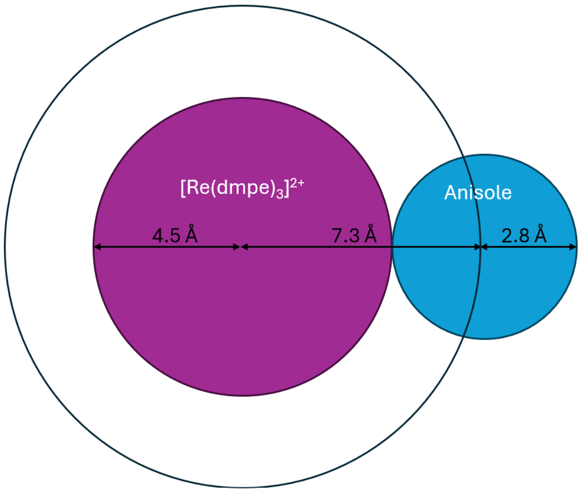

Figure S14. Schematic drawing illustrating the average center-to-center distance of $[Re(dmpe)_3]^{2+}$ and anisole.

The probability of a given number of quencher molecules (n) to be within the sphere of the photosensitizer $[Re(dmpe)_3]^{2+}$ at low concentrations can be estimated using Poisson statistics:

$$P(n) = \frac{\lambda^n}{n!} e^{-\lambda} \quad ; \quad \lambda = N_{max} \cdot c[\%]$$

where N_{max} is the maximum number of neighbors per photosensitizer and c[%] is the quencher concentration in volume%. This model ignores any specific interactions between the molecules that might favor attraction/repulsion.

Table S1. Probability of at least one molecule ($n \geq 1$) being within the solvent shell of $[Re(dmpe)_3]^{2+}$ at various low anisole concentrations based on Poisson distribution.

[anisole] [vol%]	1	3	10	30	50
[anisole] [M]	0.094	0.28	0.94	2.8	4.7
P(≥1) [%]	18	45	86	100	100

6. Excited State Dynamics of $[Re(dmpe)_3]^{2+}$

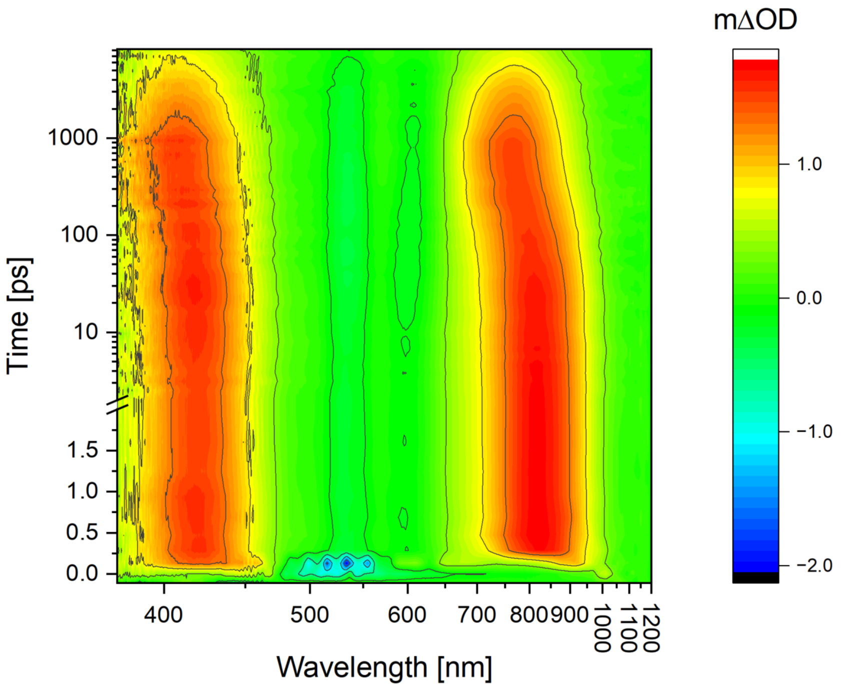

Figure S15. 2D contour plot showing the TA data of $[Re(dmpe)_3]^{2+}$ in deaerated acetonitrile at 20 °C as a function of delay time between the pump and probe. Excitation occurred at 540 nm. The polarization between pump and probe beams was set to 90° (perpendicular polarization).

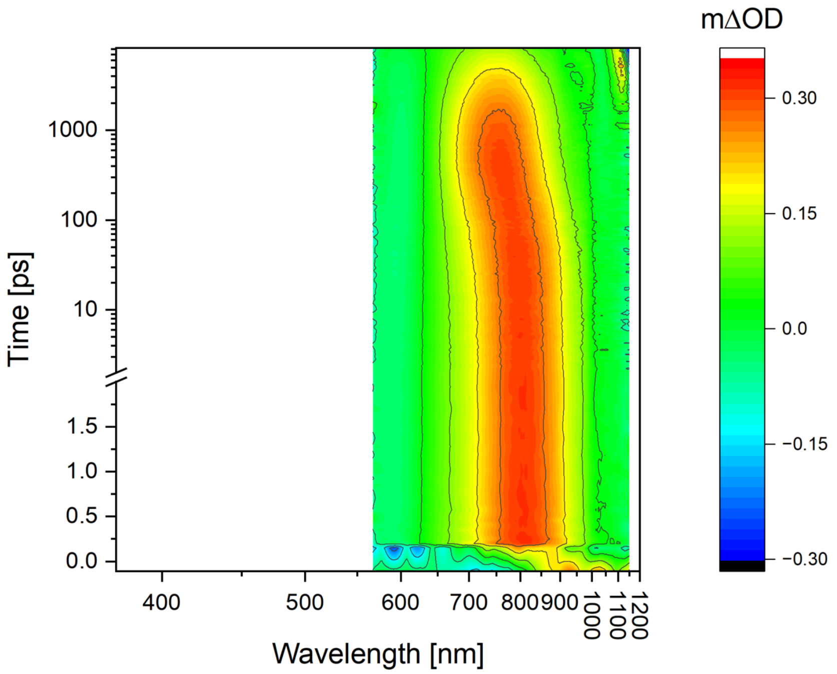

Figure S16. 2D contour plot showing the TA data of $[Re(dmpe)_3]^{2+}$ in deaerated acetonitrile at 20 °C as a function of delay time between the pump and probe. Excitation occurred at 530 nm. The polarization between pump and probe beams was set to 54.7° (magic angle). Wavelengths < 560 nm were omitted in the plot due to significant scatter around the excitation wavelength and instability of the probe light in the blue spectral region.

Global Fit Analysis

The TA data of $[Re(dmpe)_3]^{2+}$ in acetonitrile (Figure S15) was modelled with a two-step consecutive model:

$$\textbf{photogenerated }{}^2\textbf{LMCT} \xrightarrow{k_{Re,1}} \textbf{relaxed }{}^2\textbf{LMCT} \xrightarrow{k_{Re,2}} {}^2\textbf{GS}$$

where $k_{Re,1}$ represents molecular transformation occurring on the picosecond timescale and $k_{Re,2}$ is the rate constant describing the decay of the relaxed ^{2}LMCT state back to the ground state, ^{2}GS.

The rate constants for the deactivation of the individual excited states of $[Re(dmpe)_3]^{2+}$ in deaerated acetonitrile were determined to $k_{Re,1} = 5.3 \times 10^{-3}$ ps^{-1} and $k_{Re,2} = 1.1 \times 10^{-4}$ ps^{-1}, translating to lifetimes of 200 ps and 9.1 ns, respectively (Figure S17).

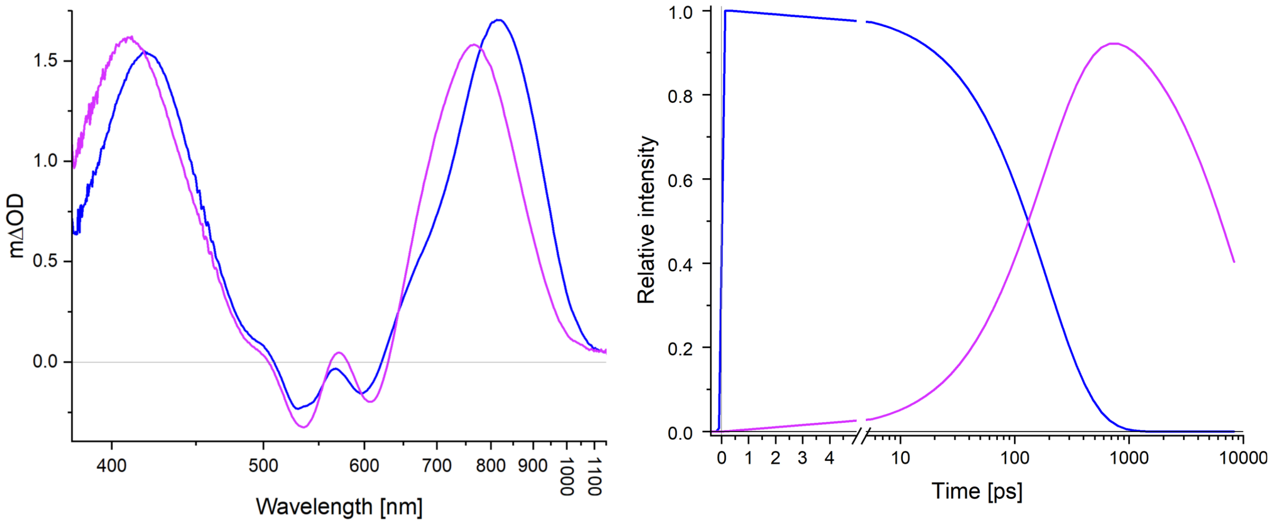

(a) (b)

Figure S17. (a) Species associated spectra associated with the photogenerated ^{2}LMCT state (blue) and the relaxed ^{2}LMCT state (pink) and the corresponding concentration profiles related to the excited state dynamics of $[Re(dmpe)_3]^{2+}$ in deaerated acetonitrile at 20 °C.

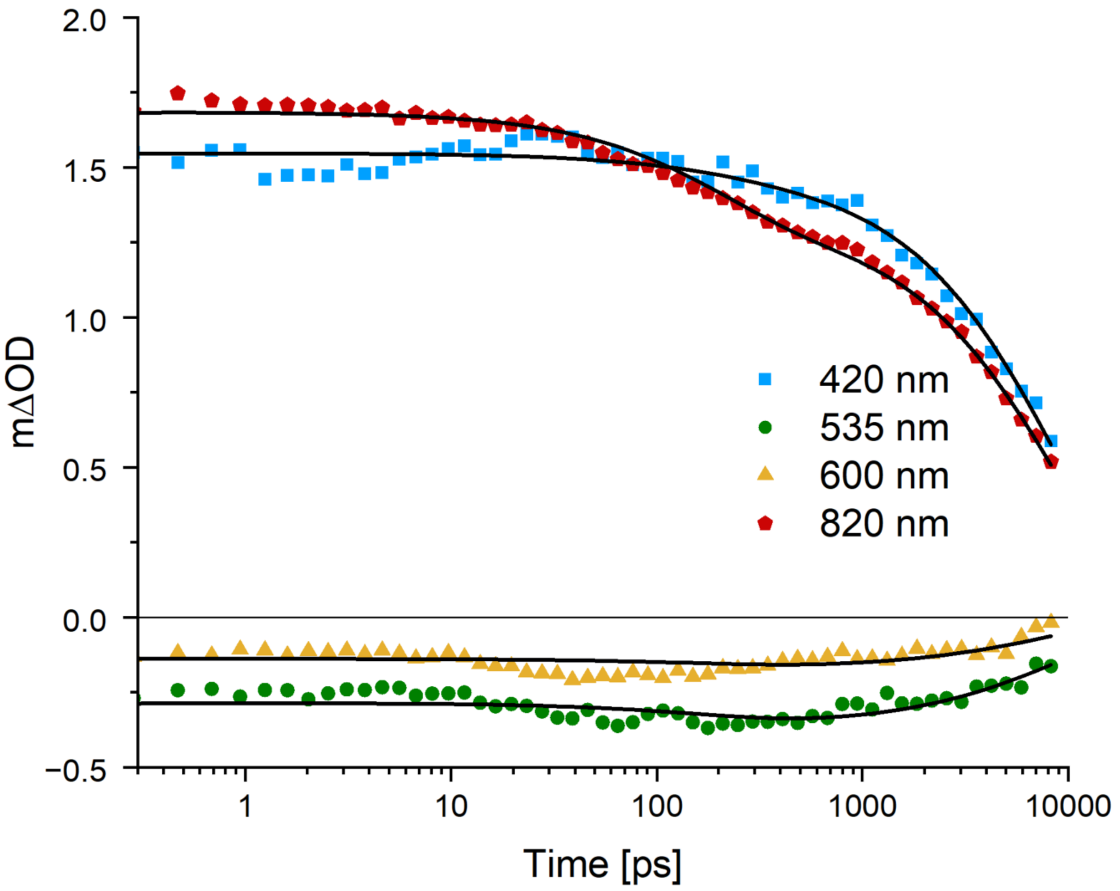

Figure S18. Measured kinetics (symbols) and the result of global fit analysis (solid lines) at selected wavelengths (see insert) for $[Re(dmpe)_3]^{2+}$ in deaerated acetonitrile. Excitation occurred at 540 nm. Data points recorded between 0 and 200 fs after the laser pulse were omitted in the global fit analysis due to the artifacts at these short time scales.

7. Excited State Dynamics – Anisole Dependence

1 vol% anisole in acetonitrile

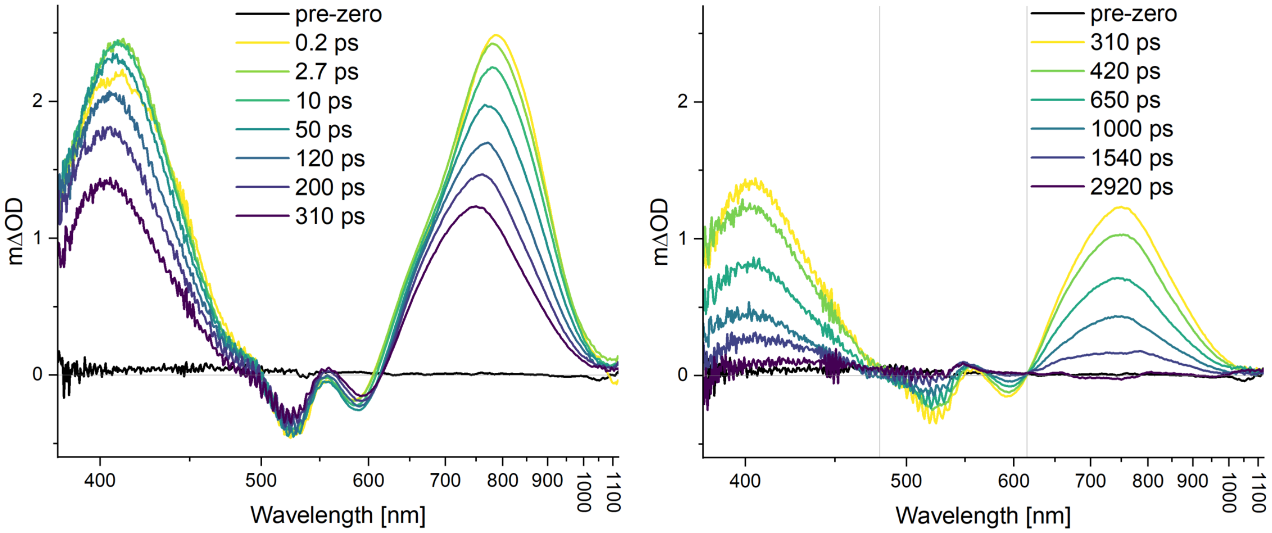

(a) (b)

Figure S19. TA spectra of $[Re(dmpe)_3]^{2+}$ in deaerated solutions of acetonitrile with 1 vol% anisole at 20 °C at (a) early and (b) later time scales. Delay times between pump and probe and their corresponding color coding are noted in ps in the inserts. Excitation occurred at 540 nm. The polarization between pump and probe beams was set to 90° (perpendicular polarization). Gray vertical lines indicate isosbestic points.

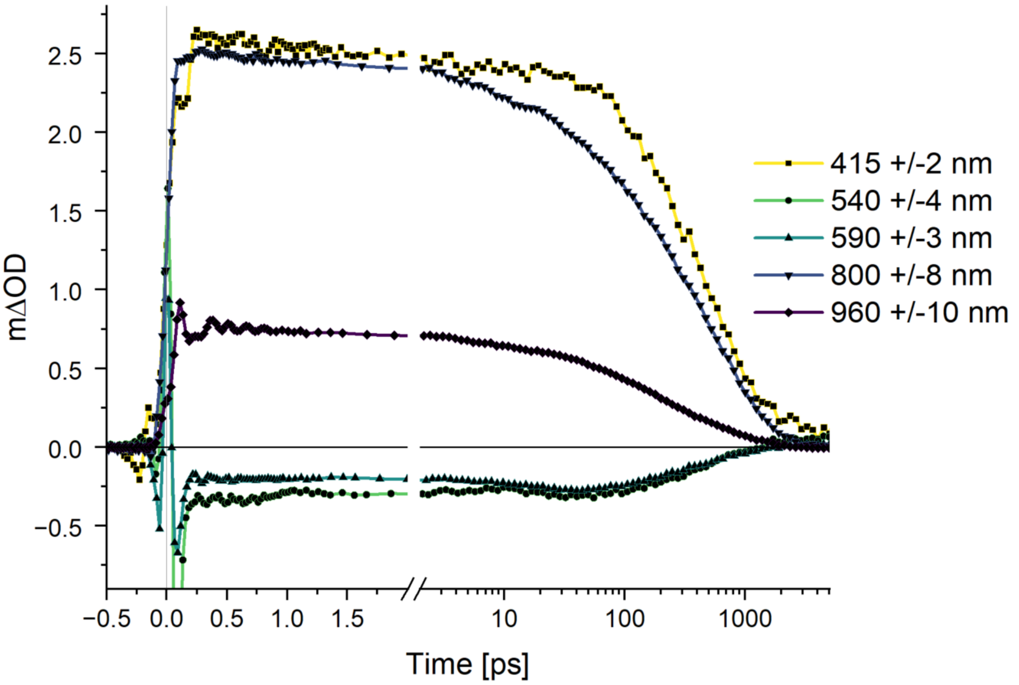

Figure S20. Measured kinetics for $[Re(dmpe)_3]^{2+}$ in deaerated solutions of acetonitrile with 1 vol% anisole at 20 °C at selected wavelengths (see color coding in insert). Excitation occurred at 540 nm.

Global fit analysis

The stimulated emission (SE) at 590 nm and the ground state bleach (GSB) at 540 nm of $[Re(dmpe)_3]^{2+}$ follow the same kinetics (Figure S20) in deaerated solutions of acetonitrile with 1 vol% anisole, which suggests that the rate of CR is faster than the rate of CS (i.e. $k_{CR} > k_{CS}$). In other words, there is no build-up of the charge separated ion pair. This observation seems reasonable as in contrast to CS no diffusion is required for recombination of the charge separated pair. Consequently, the TA data of $[Re(dmpe)_3]^{2+}$ (Figure S19) in deaerated solutions of acetonitrile with 1 vol% anisole was modelled with a two-step consecutive model:

$$\textbf{photogenerated }^{2}\textbf{LMCT} \xrightarrow{k_{Re,1}} \textbf{relaxed }^{2}\textbf{LMCT} \xrightarrow{k_{CS,diff}} {}^{2}\textbf{GS}$$

where $k_{Re,1}$ represents the rate constant related to molecular transformations occurring on the picosecond timescale and $k_{CS,diff}$ is diffusion-controlled photoinduced electron transfer rate. The rate constants were determined to $k_{Re,1} = 1.8 \times 10^{-3}$ ps^{-1} and $k_{Re,diff} = 1.8 \times 10^{-4}$ ps^{-1}, translating to lifetimes of 55 ps and 560 ns, respectively (Figure S21).

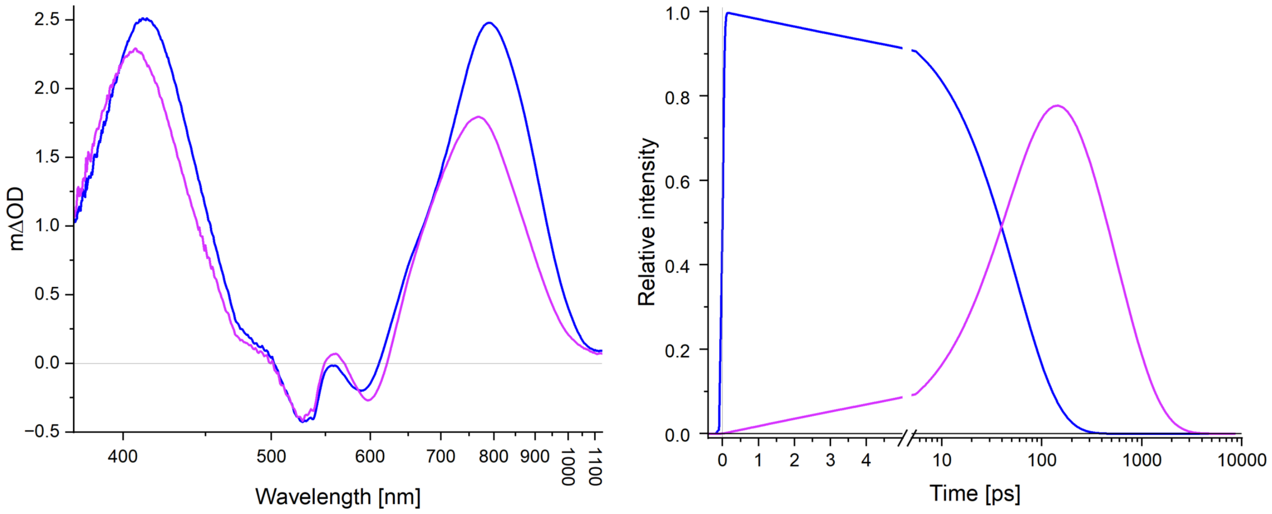

(a) (b)

Figure S21. (a) Species associated spectra associated with the photogenerated ^{2}LMCT state (blue) and the relaxed ^{2}LMCT state (pink) and (b) the corresponding concentration profiles related to the excited state dynamics of $[Re(dmpe)_3]^{2+}$ in deaerated solutions of acetonitrile with 1 vol% anisole at 20 °C.

Determination of τ_{CR}

The rate of CR is not directly observed in the TA data of $[Re(dmpe)_3]^{2+}$ in deaerated solutions of acetonitrile with 1 vol% anisole. However, the kinetic traces at 415 nm and 800 nm do not develop identically between 10-1000 ps (Figure S20). Aromatic radical cation monomers are known to absorb in the range of 400 to 450 nm.[5, 6] Against this background, it seems plausible that the kinetic trace at 800 nm are dominated by an absorption contribution only from the excited state of $[Re(dmpe)_3]^{2+}$, whereas the kinetic trace at 415 nm contains both the absorption contribution from the excited state of $[Re(dmpe)_3]^{2+}$ as well as an absorption contribution from the anisole radical cation monomer. This also means that deviation of the dynamics at 415 nm and 800 nm reflect the contribution of CR. It was therefore possible to estimate the rate of CR to 30 ps through a two-step procedure, where we first normalize the two kinetic traces at early time scales (Figure S22a) and then divided the normalized dynamics to obtain their deviation (Figure S22b).

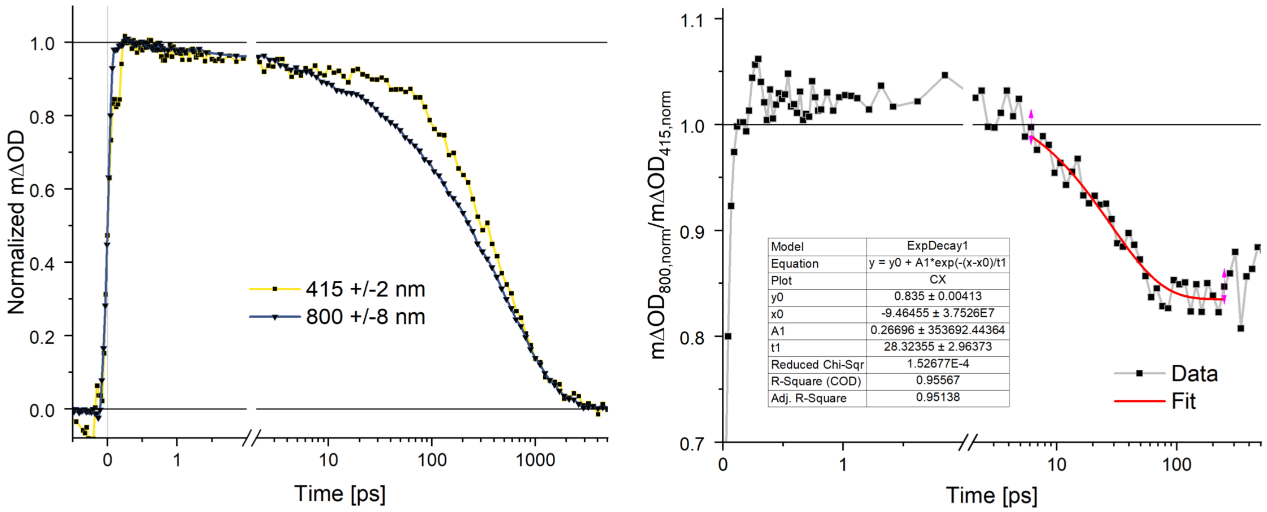

(a) (b)

Figure S22. (a) Normalized kinetics traces at 415 nm and 800 nm and (b) divided normalized kinetics traces at 415 nm and 800 nm for $[Re(dmpe)_3]^{2+}$ in deaerated solutions of acetonitrile with 1 vol% anisole.

3 vol% anisole in acetonitrile

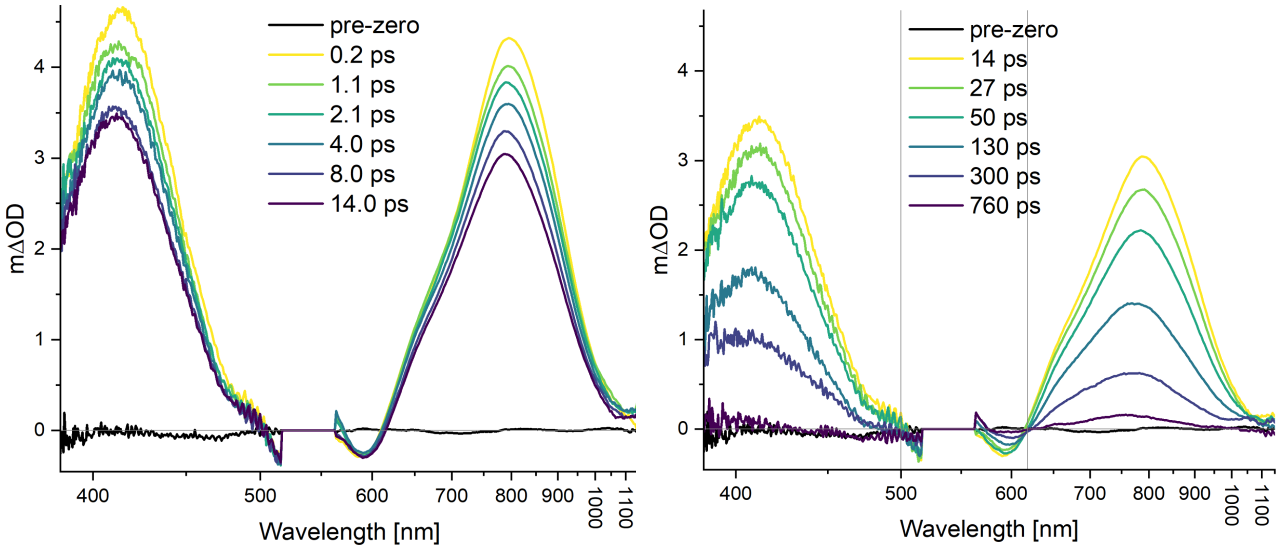

(a) (b)

Figure S23. TA spectra of $[Re(dmpe)_3]^{2+}$ in deaerated solutions of acetonitrile with 3 vol% anisole at 20 °C at (a) early and (b) later time scales. Delay times between pump and probe and their corresponding color coding are noted in ps in the inserts. Excitation occurred at 540 nm. A scratch in the cuvette caused significant scatter around the pump wavelength, thus this spectral range is omitted. Gray vertical lines indicate isosbestic points.

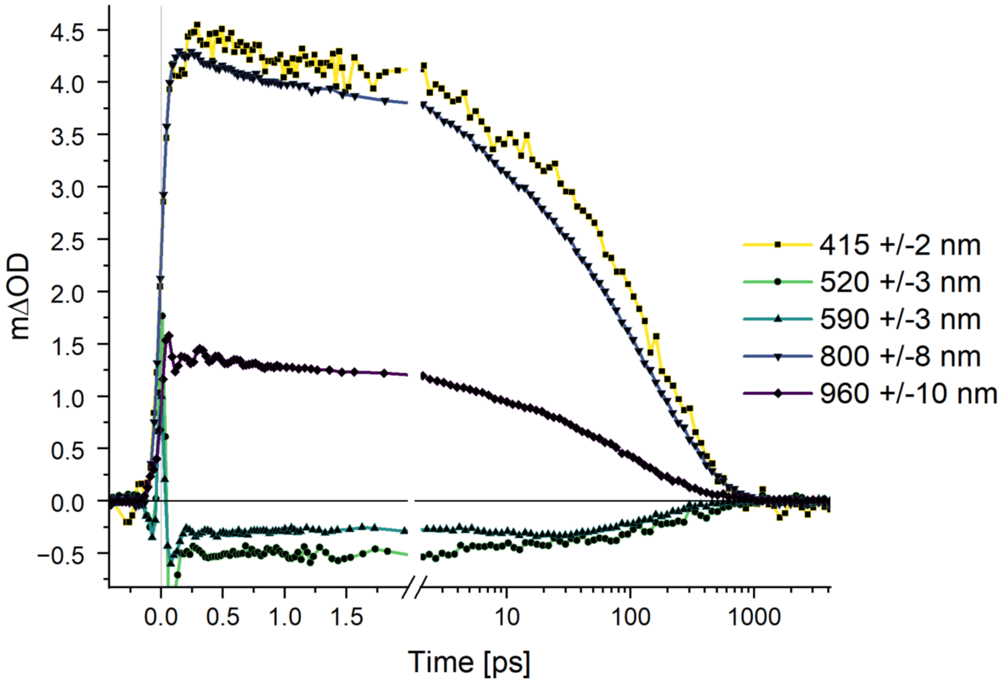

Figure S24. Measured kinetics for $[Re(dmpe)_3]^{2+}$ in deaerated solutions of acetonitrile with 3 vol% anisole at 20 °C at selected wavelengths (see color coding in insert). Excitation occurred at 540 nm.

Determination of τ_{CS}

The TA spectra of $[Re(dmpe)_3]^{2+}$ in deaerated solutions of acetonitrile with 3 vol% anisole show that the amplitudes of the ESAs at 415 nm and 800 nm decay at the picosecond time scale concurrently with a change of the SE at 590 nm (Figure S23a). This suggests that a part of the excited state population is undergoing CS independent of diffusion, because a concentration of 3 vol% anisole is high enough to give a substantial probability of having $[Re(dmpe)_3]^{2+}$ and an anisole molecule in close-contact (Table S1). In addition to this close-contact CS, also diffusion-controlled CS is observed on the sub-nanosecond time scale (Figure S23b). A biexponential fit function allowed for the estimation of these two time components to 7 ps and 170 ps, respectively (Figure S25).

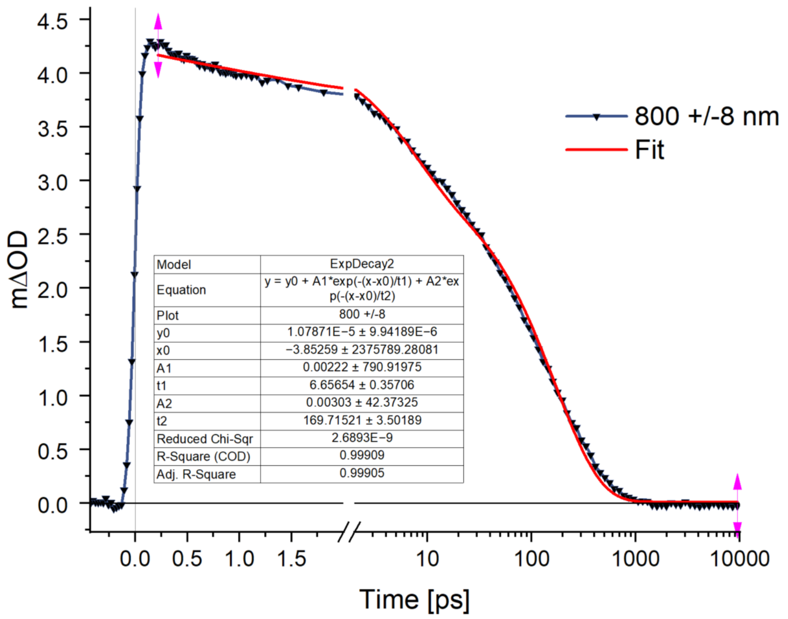

Figure S25. Measured kinetics for $[Re(dmpe)_3]^{2+}$ in deaerated solutions of acetonitrile with 3 vol% anisole at 20 °C at 800 nm and a biexponential fit to estimate the time components related to CS relaying on either close contact (7 ps) or diffusion (170 ps) between $[Re(dmpe)_3]^{2+}$ and anisole Excitation occurred at 540 nm.

Determination of τ_{CR}

With the same arguments as for the analysis of the TA data in the presence of 1 vol% anisole (Figure S22), the rate of CR was estimated to 25 ps in the photocycle of $[Re(dmpe)_3]^{2+}$ in deaerated solutions of acetonitrile with 3 vol% anisole (Figure S26).

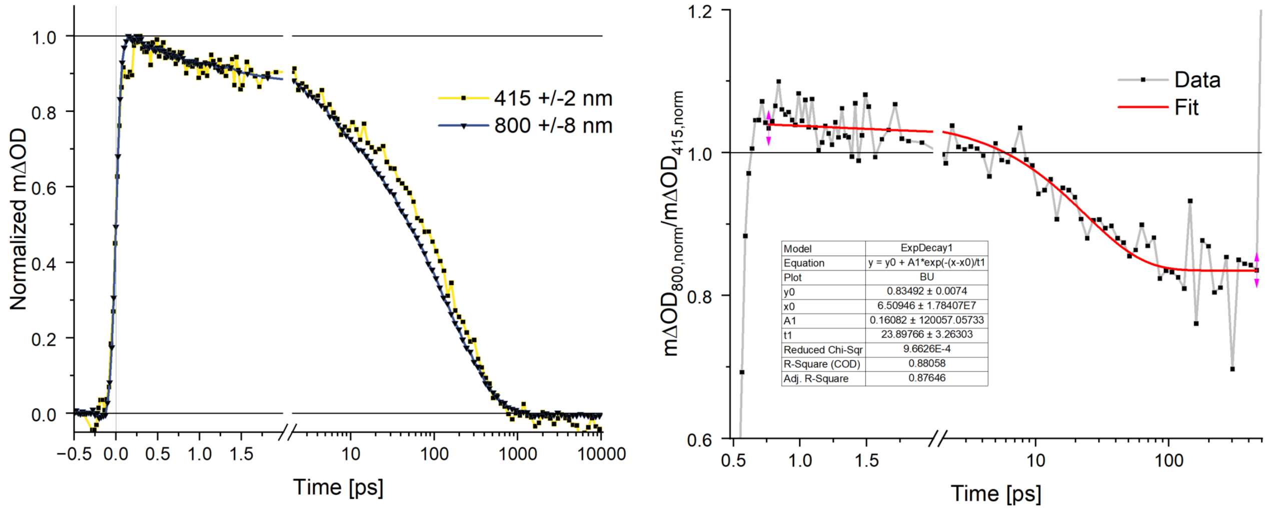

(a) (b)

Figure S26. (a) Normalized kinetics traces at 415 nm and 800 nm and (b) divided normalized kinetics traces at 415 nm and 800 nm for $[Re(dmpe)_3]^{2+}$ in deaerated solutions of acetonitrile with 3 vol% anisole.

10 vol% anisole in acetonitrile

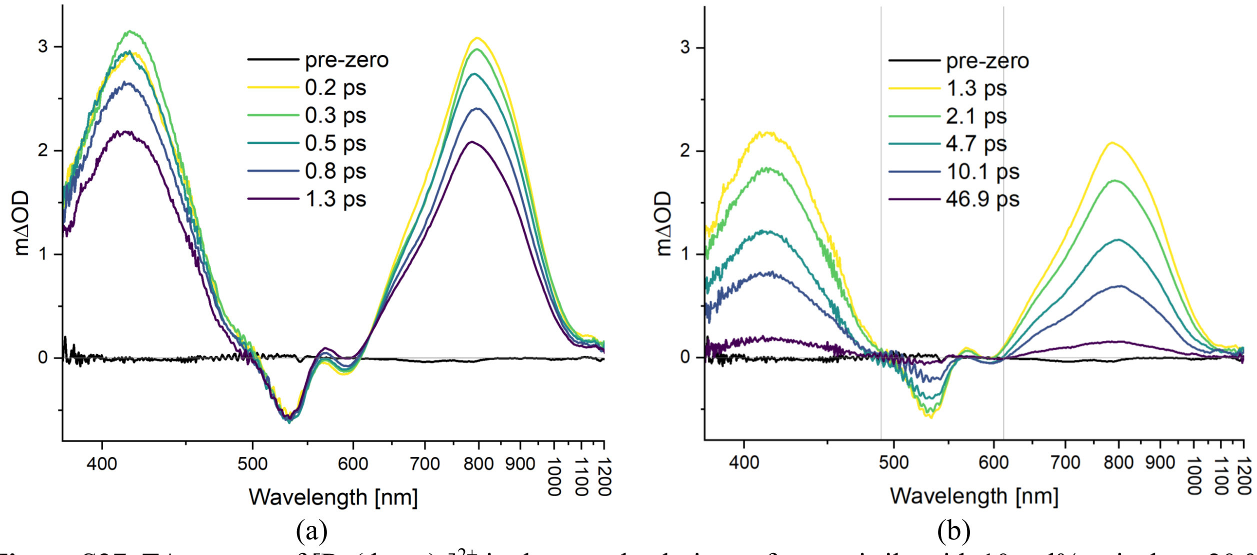

Figure S27. TA spectra of $[Re(dmpe)_3]^{2+}$ in deaerated solutions of acetonitrile with 10 vol% anisole at 20 °C at (a) early and (b) later time scales. Delay times between pump and probe and their corresponding color coding are noted in ps in the inserts. Excitation occurred at 540 nm. Gray vertical lines indicate isosbestic points.

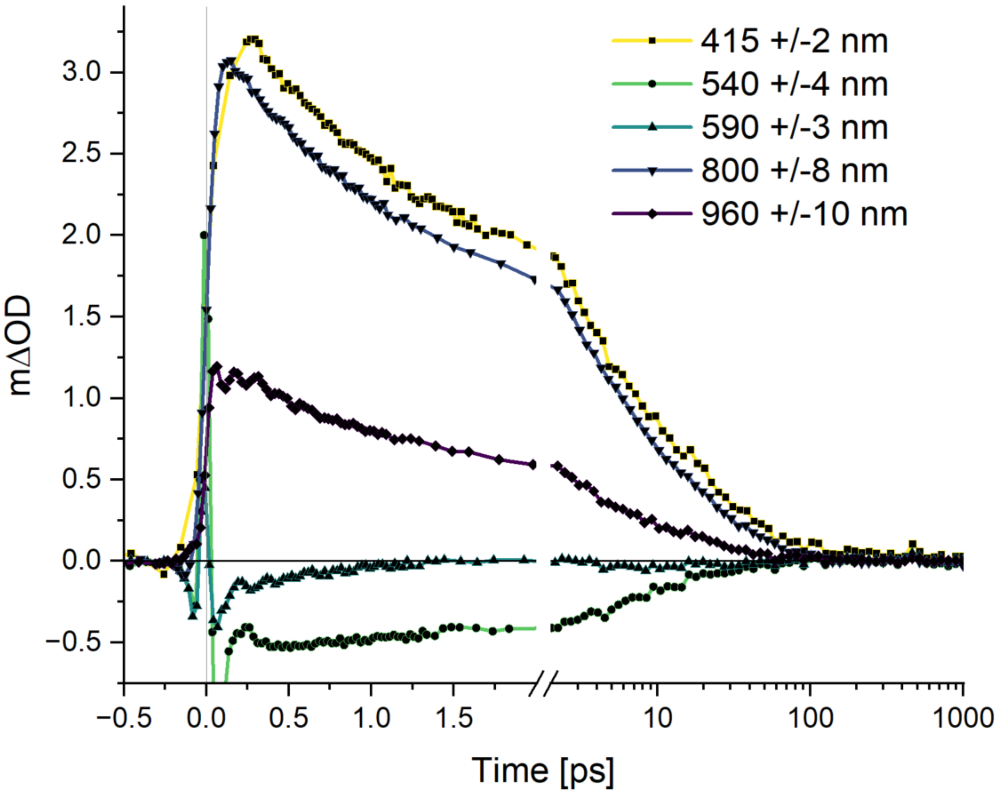

Figure S28. Measured kinetics for $[Re(dmpe)_3]^{2+}$ in deaerated solutions of acetonitrile with 10 vol% anisole at 20 °C at selected wavelengths (see color coding in insert). Excitation occurred at 540 nm.

Determination of τ_{CS} and τ_{CR}

The isosbestic point at 630 nm observed in the TA data of $[Re(dmpe)_3]^{2+}$ in deaerated solutions of acetonitrile with 10 vol% anisole (Figure S27a) suggests that the rate of CR is slower than the rate of close-contact CS in the photocycle. The time component related to close-contact CS can therefore be estimated from the growth of the spectral feature associated with the stimulated emission at 590 nm, whereas the subsequent disappearance of the 590 nm signal to zero differential absorption is associated with the CR process (Figure S29). The time components will naturally also be present in kinetic traces at other key wavelengths such as at 800 nm, however, at 590 nm the two processes have conveniently opposite signs i.e. CS is related to *a growth*, whereas CR is related to *a decay*. In other words, it is easier to separate the individual contributions at 590 nm than at other wavelengths.

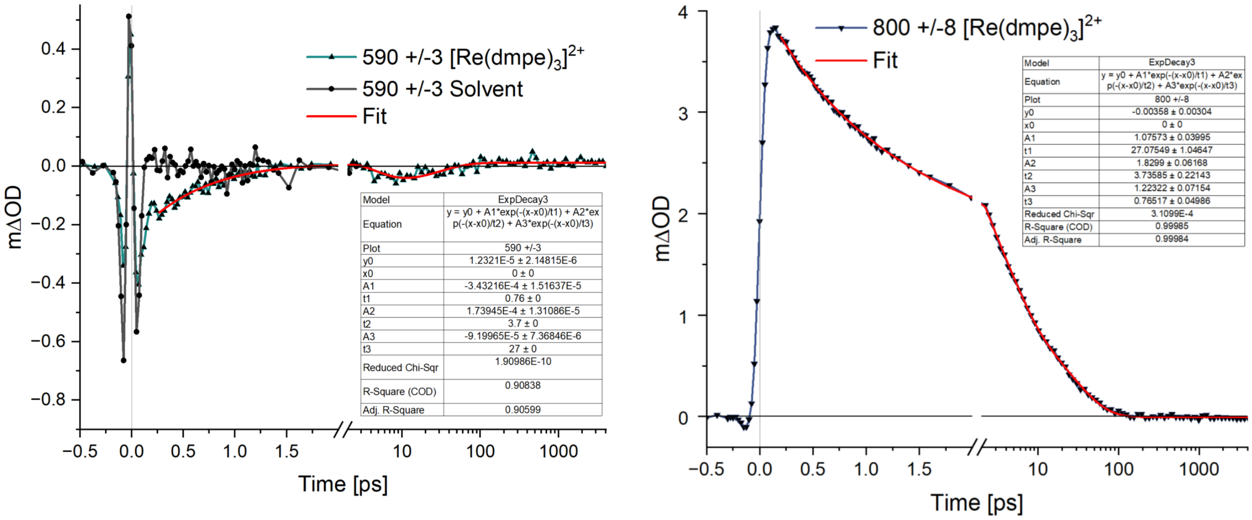

(a) (b)

Figure S29. Measured kinetics (symbols) and fit functions (red solid) at (a) 590 nm and (b) 800 nm of deaerated solutions of $[Re(dmpe)_3]^{2+}$ in acetonitrile with 10 vol% anisole at 20 °C. The solvent response (gray) is included in (a) to judge at what time point the dynamics are free of coherent artefacts at the ultrafast timescales. Excitation occurred at 540 nm.

In the presence of 10 vol% anisole, the dynamics at 590 nm is, however, on the order of the noise level, which challenges a reliable fit at this wavelength. Moreover, there are some slower dynamics occurring on the nanosecond timescale due to diffusion-controlled CS. This means that only a part of the excited state population is quenched by close-contact CS in the presence of 10 vol% anisole, whereas the remaining of the excited state dynamics is quenched by diffusion-controlled dynamics. The rates in the photocycle of $[Re(dmpe)_3]^{2+}$ in the presence of 10 vol% anisole was thus estimated by using a triexponential fit function for the decay at 800 nm (Figure S29b). This approach resulted in time components of 0.8 ps, 3.7 ps and 27 ps translating to close-contact CS, CR and diffusion-controlled CS, respectively. To validate the 800 nm fit, the

kinetic trace at 590 nm was fitted with a triexponential fit function, where the time components were fixed to 0.8 ps, 3.7 ps and 27 ps. The resulting fit agrees very well with the experimental data at 590 nm (Figure S29a).

30 vol% anisole in acetonitrile

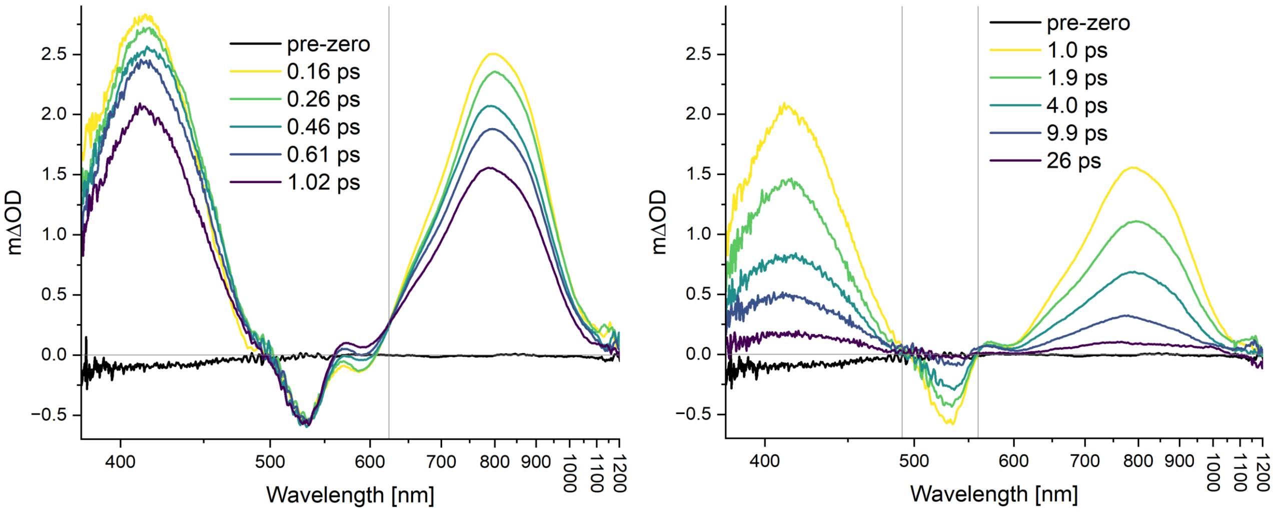

(a) (b)

Figure S30. TA spectra of $[Re(dmpe)_3]^{2+}$ in deaerated solutions of acetonitrile with 30 vol% anisole at 20 °C at (a) early and (b) later time scales. Delay times between pump and probe and their corresponding color coding are noted in ps in the inserts. Excitation occurred at 540 nm. Gray vertical lines indicate isosbestic points.

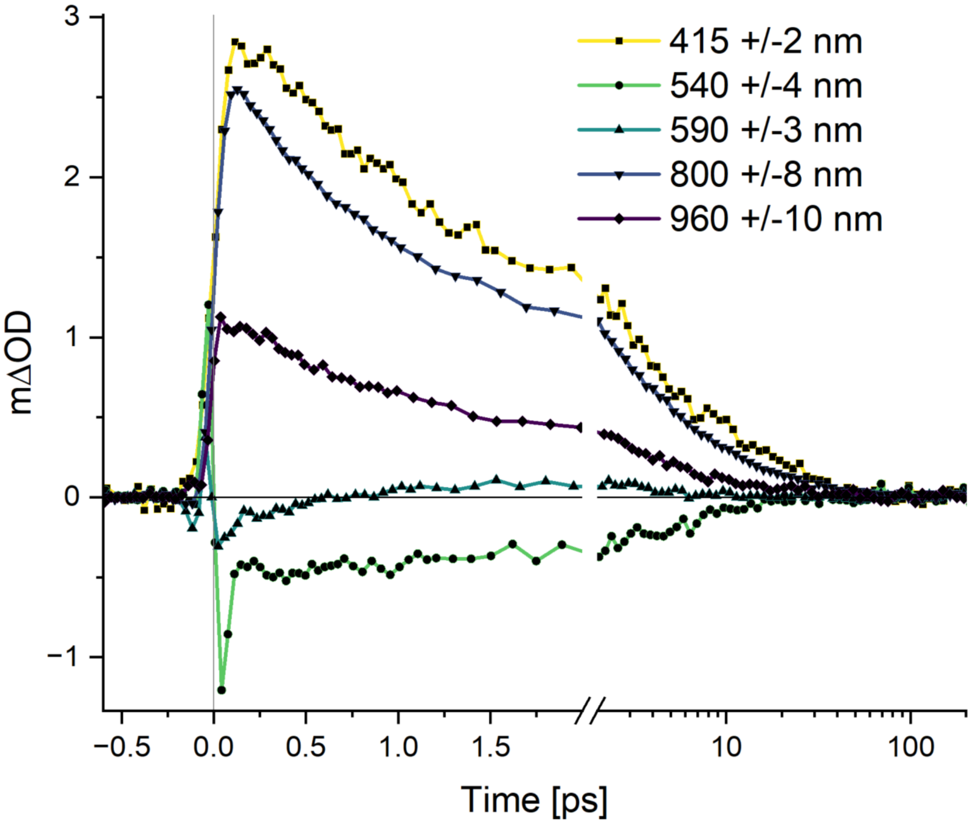

Figure S31. Measured kinetics for $[Re(dmpe)_3]^{2+}$ in deaerated solutions of acetonitrile with 30 vol% anisole at 20 °C at selected wavelengths (see color coding in insert). Excitation occurred at 540 nm.

Determination of τ_{CS} and τ_{CR}

The isosbestic point at 630 nm observed in the TA data for $[Re(dmpe)_3]^{2+}$ in deaerated solutions of acetonitrile with 30 vol% anisole (Figure S30a) suggests that the rate of CR is slower than the rate of CS in the photocycle. The time component related to close-contact CS can be estimated from the sub-ps growth of the spectral feature associated with the stimulated emission at 590 nm (Figure S32a), whereas the subsequent disappearance of the 590 nm signal to zero differential absorption is related to the CR process. The time components will naturally also be present in kinetic traces at other key wavelengths (e.g. at 800 nm, Figure S32b), however, at 590 nm the two processes have conveniently opposite signs i.e. CS is related to *a growth*, whereas CR is related to *a decay*. In other words, there is a clearer separation of the individual contributions in the kinetics at 590 nm than at other wavelengths. Using a biexponential fit function for the kinetic trace at 590 nm, a time component of 0.6 ps was found to be related to the growth of the signal (CS). The fit of the 590 nm kinetics was, however, not very sensitive to the value of the second time-component. The second time component was thus determined by fitting the kinetic trace at 800 nm with a biexponential fit function, where one time component was fixed to 0.6 ps. In this way, a second time-component of 4 ps could be determined (CR).

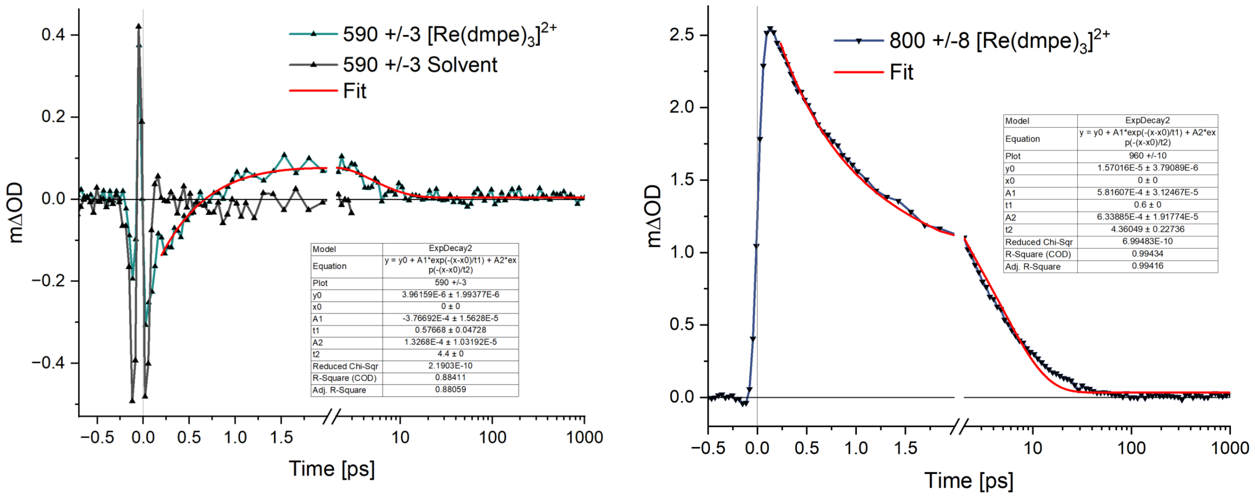

(a) (b)

Figure S32. Measured kinetics (symbols) and fit functions (red solid) at (a) 590 nm and (b) 800 nm of deaerated solutions of $[Re(dmpe)_3]^{2+}$ in acetonitrile with 30 vol% anisole at 20 °C. The solvent response (gray) is included in (a) to judge at what time point the dynamics are free of coherent artefacts at the ultrafast timescales. Excitation occurred at 540 nm.

50 vol% anisole in acetonitrile

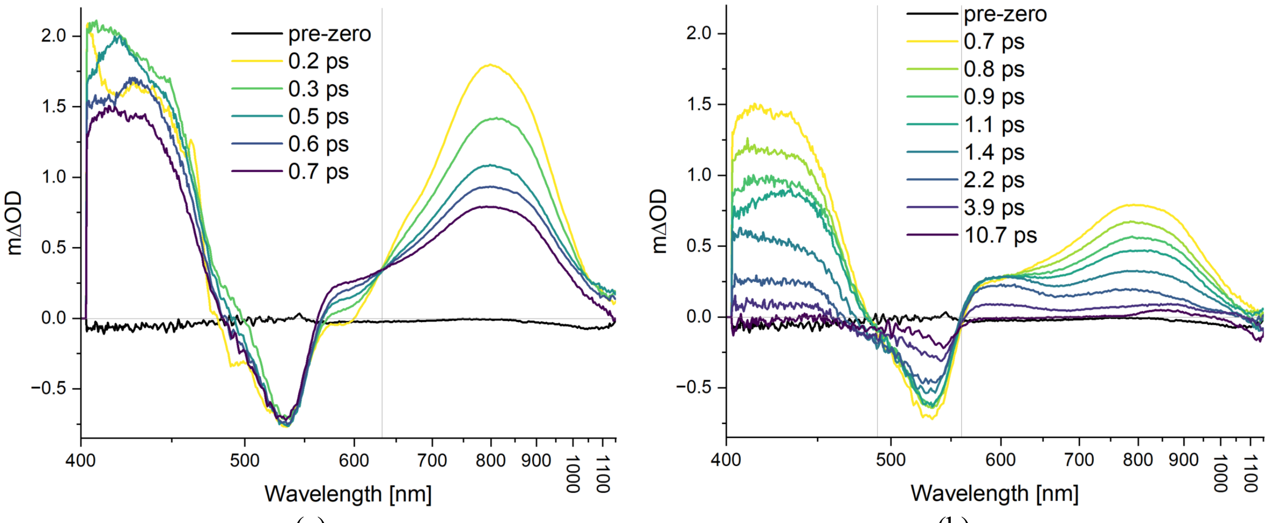

Figure S33. TA spectra of $[Re(dmpe)_3]^{2+}$ in deaerated solutions of acetonitrile with 50 vol% anisole at 20 °C at (a) early and (b) later time scales. Delay times between pump and probe and their corresponding color coding are noted in ps in the inserts. Excitation occurred at 540 nm. Gray vertical lines indicate isosbestic points.

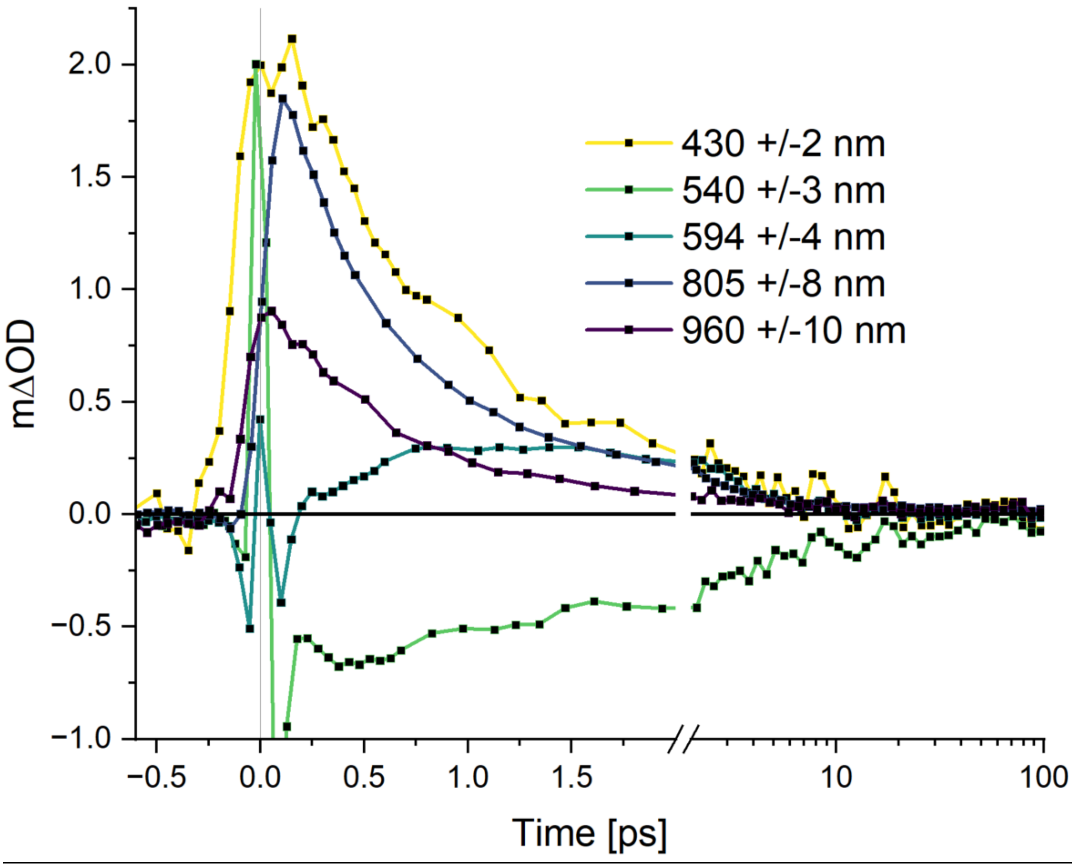

Figure S34. Measured kinetics for $[Re(dmpe)_3]^{2+}$ in deaerated solutions of acetonitrile with 50 vol% anisole at 20 °C at selected wavelengths (see color coding in insert). Excitation occurred at 540 nm.

Determination of τ_{CS} and τ_{CR}

The isosbestic point at 630 nm observed in the TA data for $[Re(dmpe)_3]^{2+}$ in deaerated solutions of acetonitrile with 50 vol% anisole (Figure S33a) suggests that the rate of CR is slower than the rate of CS in the photocycle. Based on analogous arguments as for the photocycle in the presence of 30 vol% anisole (Figure S32) and using a biexponential fit function for the kinetic trace at 594 nm (Figure S35a), a time component of 0.5 ps was found to be related to CS and a time component of 2.3 ps was found to be related to CR. To validate the 594 nm fit, the kinetic trace at 805 nm was likewise fitted with a biexponential fit function, but in this fit the time components were fixed to 0.5 ps and 2.3 ps. The resulting fit agrees very well with the experimental data at 805 nm (Figure S35b).

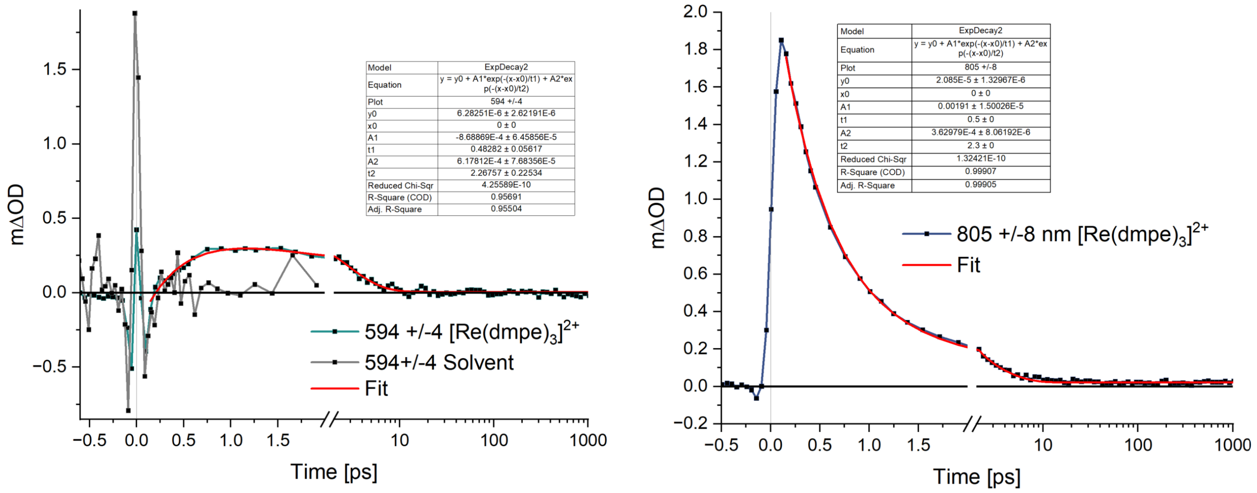

(a) (b)

Figure S35. Measured kinetics (symbols) and fit functions (red solid) at (a) 594 nm and (b) 805 nm of deaerated solutions of $[Re(dmpe)_3]^{2+}$ in acetonitrile with 50 vol% anisole at 20 °C. The solvent response (gray) is included in (a) to judge at what time point the dynamics are free of coherent artefacts at the ultrafast timescales. Excitation occurred at 540 nm.

60 vol% anisole in acetonitrile (equivalent to 5.7 M)

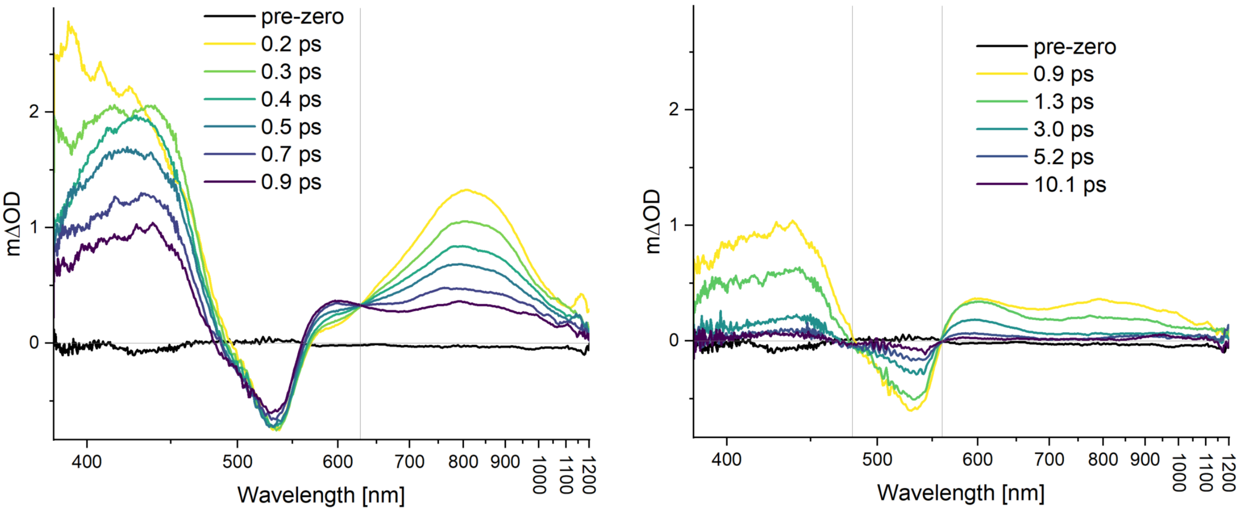

(a) (b)

Figure S36. TA spectra of $[Re(dmpe)_3]^{2+}$ in deaerated solutions of acetonitrile with 60 vol% anisole at 20 °C at (a) early and (b) later time scales. Delay times between pump and probe and their corresponding color coding are noted in ps in the inserts. Excitation occurred at 540 nm. Gray vertical lines indicate isosbestic points.

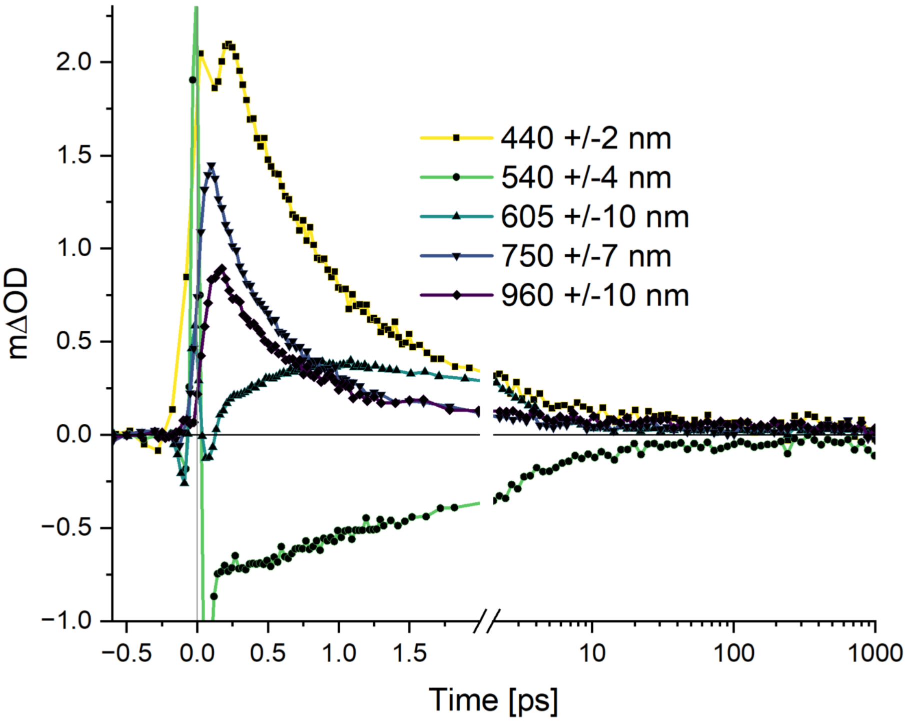

Figure S37. Measured kinetics for $[Re(dmpe)_3]^{2+}$ in deaerated solutions of acetonitrile with 60 vol% anisole at 20 °C at selected wavelengths (see color coding in insert). Excitation occurred at 540 nm. The kinetic trace of the GSB at 540 nm does not return to zero differential absorption at longer time scales indicating minor CE at this high quencher concentration. Comparing the amplitudes at 540 nm at 0.3 ps with that at 1 ns suggests that 5-10 % of the excited state population has not returned to the ground state, which would translate to the CE yield.

Determination of τ_{CS} and τ_{CR}

The isosbestic point at 630 nm observed in the TA spectra for $[Re(dmpe)_3]^{2+}$ in deaerated solutions of acetonitrile with 60 vol% anisole (Figure S36a) suggests that the rate of CR is slower than the rate of CS in the photocycle. Based on analogous arguments as for the photocycle in the presence of 30 vol% anisole (Figure S32) and using a biexponential fit function for the kinetic trace at 605 nm, a time component of 0.5 ps was found to be related to CS and a time component of 2.2 ps was found to be related to CR (Figure S38a). To validate the 605 nm fit, the kinetic trace at 750 nm was likewise fitted with a biexponential fit function, but in this fit the time components were fixed to 0.5 ps and 2.2 ps. The resulting fit agrees very well with the experimental data at 750 nm (Figure S38b).

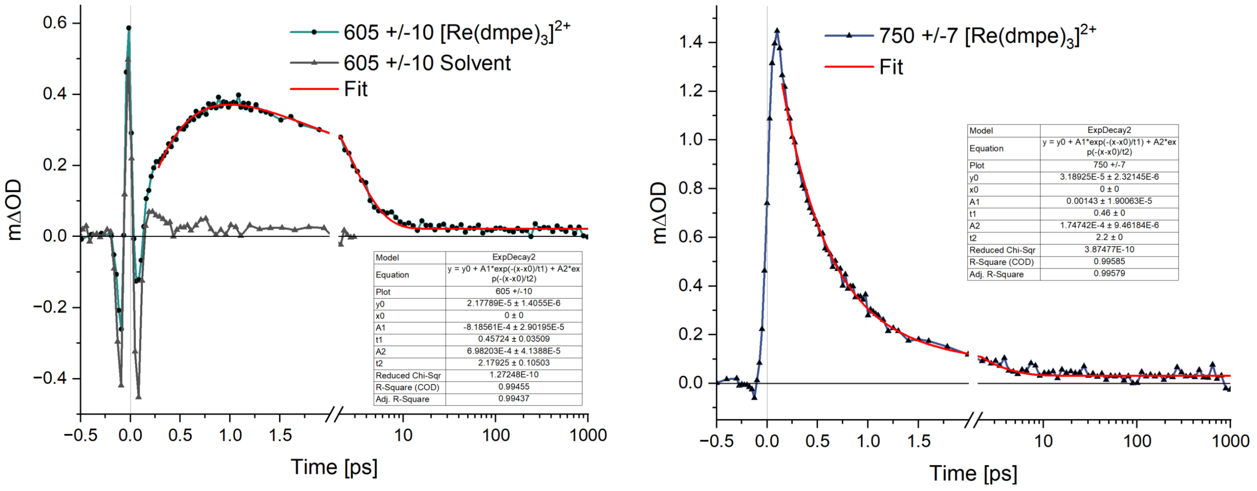

(a) (b)

Figure S38. Measured kinetics (symbols) and fit functions (red solid) at (a) 605 nm and (b) 750 nm of deaerated solutions of $[Re(dmpe)_3]^{2+}$ in acetonitrile with 60 vol% anisole at 20 °C. The solvent response (gray) is included in (a) to judge at what time point the dynamics are free of coherent artefacts at the ultrafast timescales. Excitation occurred at 540 nm.

70 vol% anisole in acetonitrile

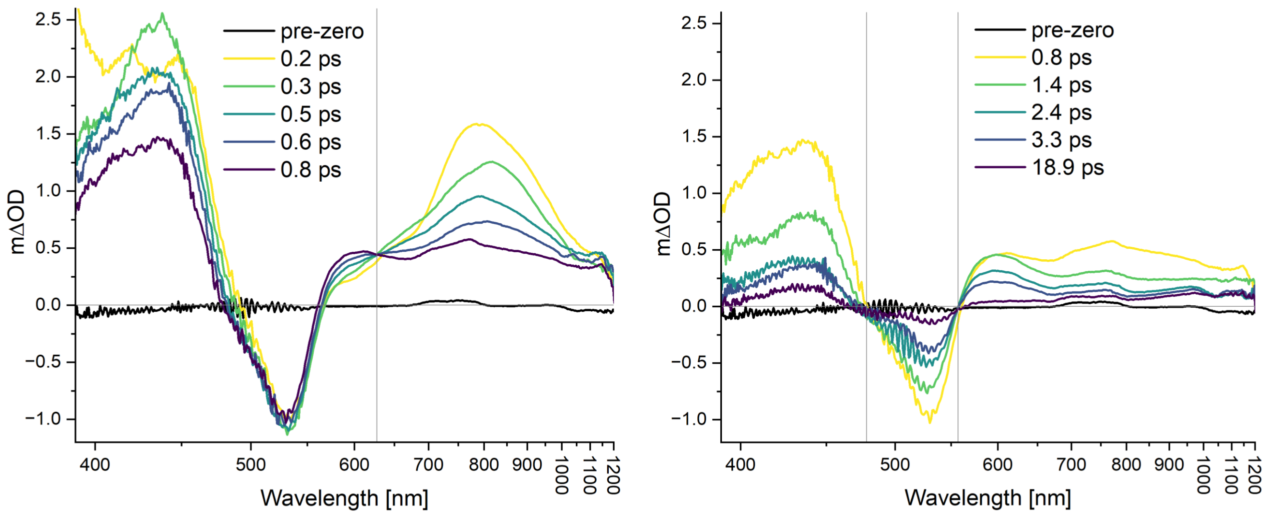

Figure S39. TA spectra of $[Re(dmpe)_3]^{2+}$ in deaerated solutions of acetonitrile with 70 vol% anisole at 20 °C at (a) early and (b) later time scales. Delay times between pump and probe and their corresponding color coding are noted in ps in the inserts. Excitation occurred at 540 nm. Gray vertical lines indicate isosbestic points.

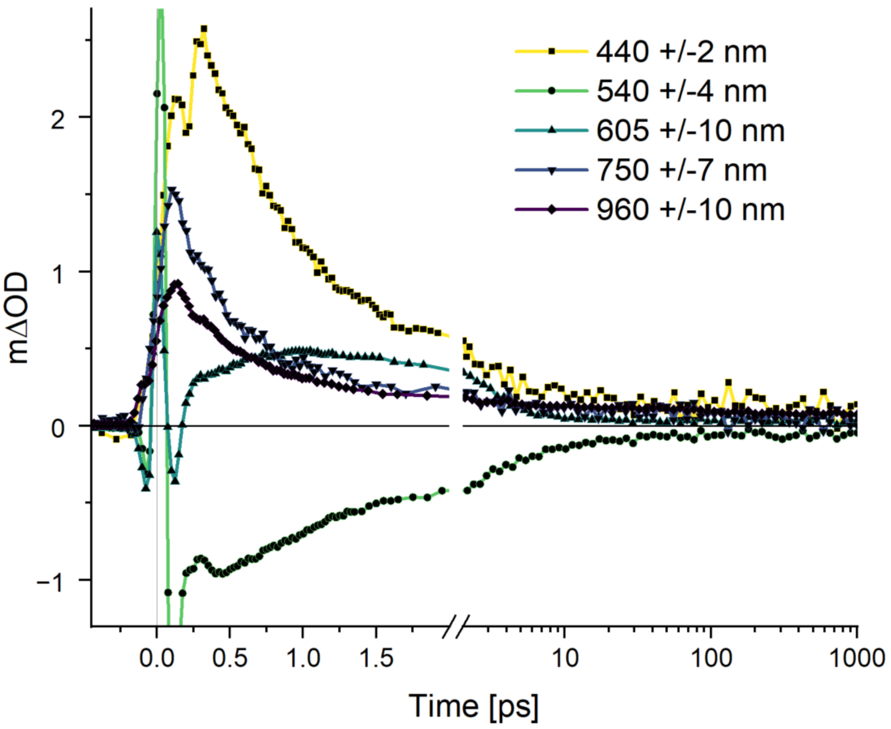

Figure S40. Measured kinetics for $[Re(dmpe)_3]^{2+}$ in deaerated solutions of acetonitrile with 70 vol% anisole at 20 °C at selected wavelengths (see color coding in insert). Excitation occurred at 540 nm. The kinetic trace of the GSB at 540 nm does not return to zero differential absorption at longer time scales indicating minor CE at this high quencher concentration. Comparing the amplitudes at 540 nm at 0.3 ps with that at 1 ns suggests that 5-10 % of the excited state population has not returned to the ground state, which would translate to the CE yield.

Determination of τ_{CS} and τ_{CR}

The isosbestic point at 630 nm observed in the TA spectra for $[Re(dmpe)_3]^{2+}$ in deaerated solutions of acetonitrile with 70 vol% anisole (Figure S39a) suggests that the rate of CR is slower than the rate of CS in the photocycle. Based on analogous arguments as for the photocycle in the presence of 30 vol% anisole (Figure S32) and using a biexponential fit function for the kinetic trace at 605 nm, a time component of 0.5 ps was found to be related to CS and a time component of 1.8 ps was found to be related to CR (Figure S41a). To validate the 605 nm fit, the kinetic trace at 750 nm was likewise fitted with a biexponential fit function, but in this fit the time components were fixed to 0.5 ps and 1.8 ps. The resulting fit agrees very well with the experimental data at 750 nm (Figure 41b).

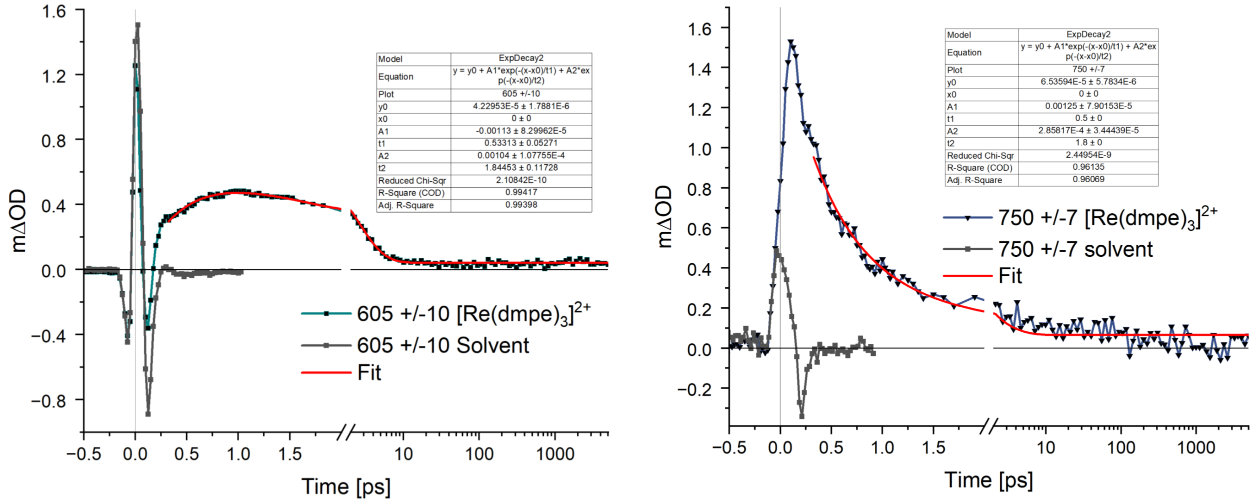

(a) (b)

Figure S41. Measured kinetics (symbols) and fit functions (red solid) at (a) 605 nm and (b) 750 nm of deaerated solutions of $[Re(dmpe)_3]^{2+}$ in acetonitrile with 70 vol% anisole at 20 °C. Solvent responses (gray) are included to judge at what time point the dynamics are free of coherent artefacts at the ultrafast timescales. Excitation occurred at 540 nm.

80 vol% anisole in acetonitrile

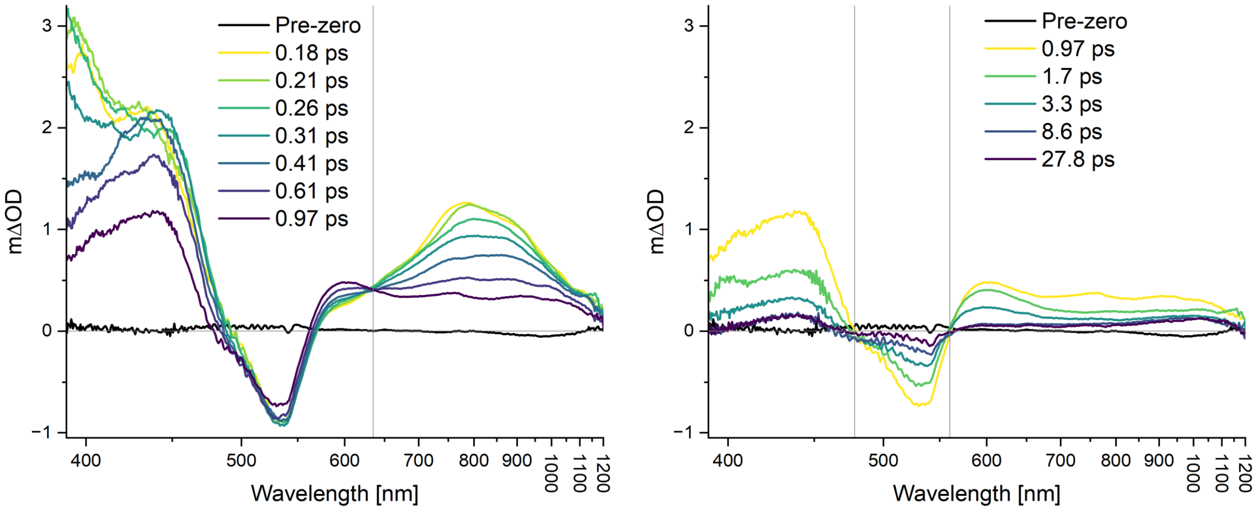

(a)42a (b)

Figure S42. TA spectra of $[Re(dmpe)_3]^{2+}$ in deaerated solutions of acetonitrile with 80 vol% anisole at 20 °C at (a) early and (b) later time scales. Delay times between pump and probe and their corresponding color coding are noted in ps in the inserts. Excitation occurred at 540 nm. Gray vertical lines indicate isosbestic points.

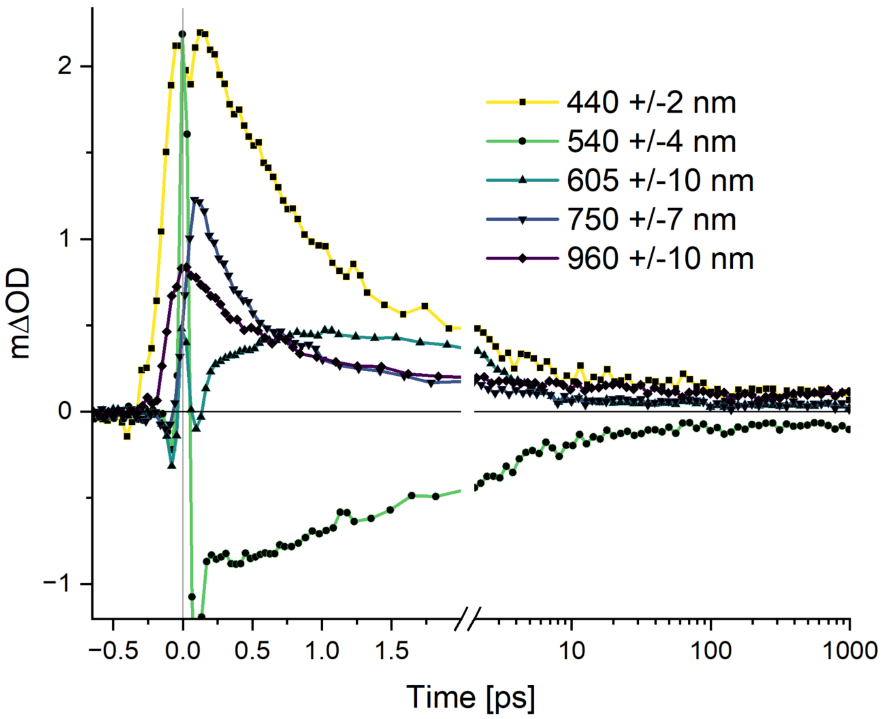

Figure S43. Measured kinetics for $[Re(dmpe)_3]^{2+}$ in deaerated solutions of acetonitrile with 80 vol% anisole at 20 °C at selected wavelengths (see color coding in insert). Excitation occurred at 540 nm. The kinetic trace of the GSB at 540 nm does not return to zero differential absorption at longer time scales indicating CE at this high quencher concentration. Comparing the amplitudes at 540 nm at 0.3 ps with that at 1 ns suggests that 10-15 % of the excited state population has not returned to the ground state, which would translate to the CE yield.

Determination of τ_{CS} and τ_{CR}

The isosbestic point at 630 nm observed in the TA spectra for $[Re(dmpe)_3]^{2+}$ in deaerated solutions of acetonitrile with 80 vol% anisole (Figure S42a) suggests that the rate of CR is slower than the rate of CS in the photocycle. The time component related to close-contact CS can be estimated from the growth of the spectral feature associated with the stimulated emission at 605 nm (Figure S44a), whereas the subsequent disappearance of the 605 nm signal to zero differential absorption is related to the CR process. The time components will naturally also be present in kinetic traces at other key wavelengths, however, at 605 nm the two processes have conveniently opposite signs i.e. CS is related to *a growth*, whereas CR is related to *a decay*. In other words, there is a clearer separation of the individual contributions in the kinetics at 605 nm than at other wavelengths. The kinetic trace at 605 nm has not returned to zero differential absorption on the nanosecond time scale in the presence of 80 vol% anisole, which suggests that the entire excited state population has not returned to the ground state. This effect could be due to CE. A triexponential decay function was thus used to fit the kinetic trace a 605 nm to allow for the presence of a long-lived decay component (Figure S44a). By doing so, a time component of 0.5 ps was found to be related to the growth of the signal (CS) and a time component of 2.2 ps was found to be related to the decay of the signal (CR). To validate the 605 nm fit, the kinetic trace at 750 nm was likewise fitted with a triexponential fit function, but in this fit the time components were fixed to 0.5 ps, 2.2 ps and 1247 ps. The resulting fit agrees very well with the experimental data at 750 nm (Figure S44b).

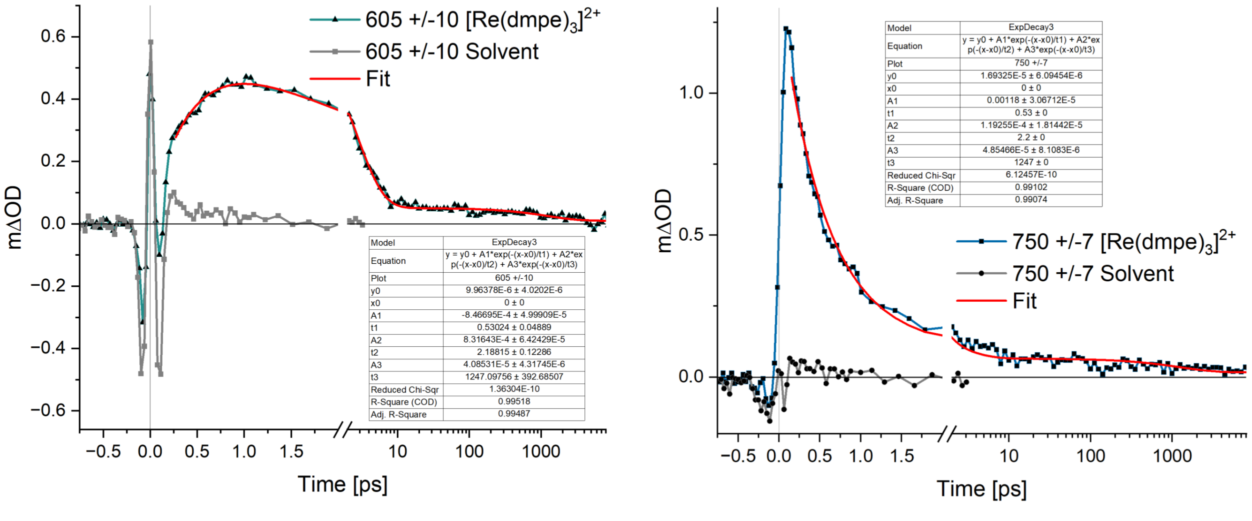

(a) (b)

Figure S44. Measured kinetics (symbols) and fit functions (red solid) at (a) 605 nm and (b) 750 nm of deaerated solutions of $[Re(dmpe)_3]^{2+}$ in acetonitrile with 80 vol% anisole at 20 °C. Solvent responses (gray) are included to judge at what time point the dynamics are free of coherent artefacts at the ultrafast timescales. Excitation occurred at 540 nm.

<u>90 vol% anisole in acetonitrile</u>

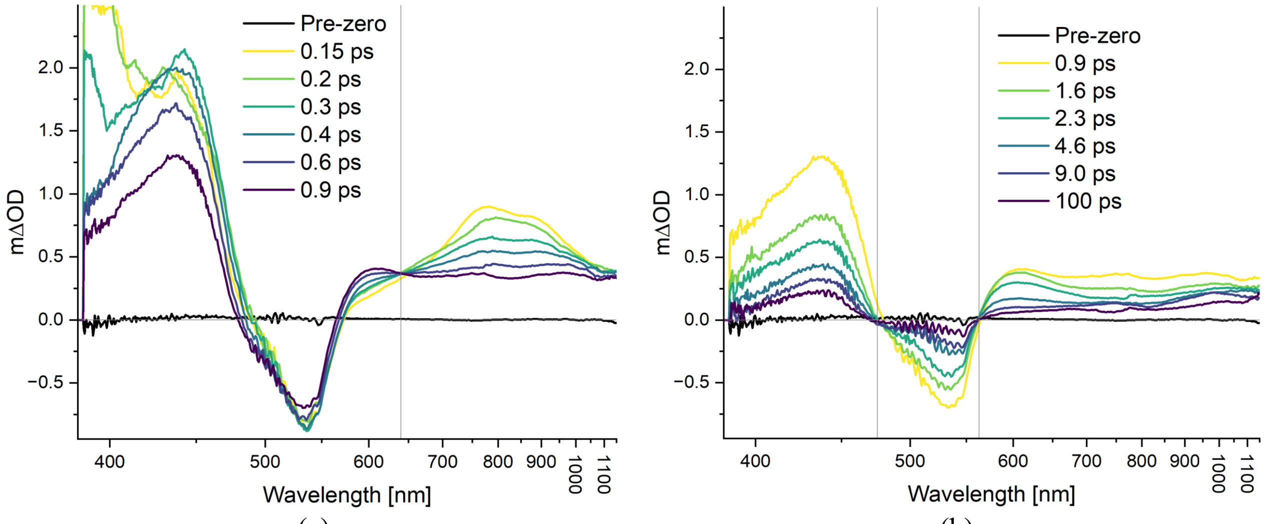

Figure S45. TA spectra of $[Re(dmpe)_3]^{2+}$ in deaerated solutions of acetonitrile with 90 vol% anisole at 20 °C at (a) early and (b) later time scales. Delay times between pump and probe and their corresponding color coding are noted in ps in the inserts. Excitation occurred at 540 nm. Gray vertical lines indicate isosbestic points.

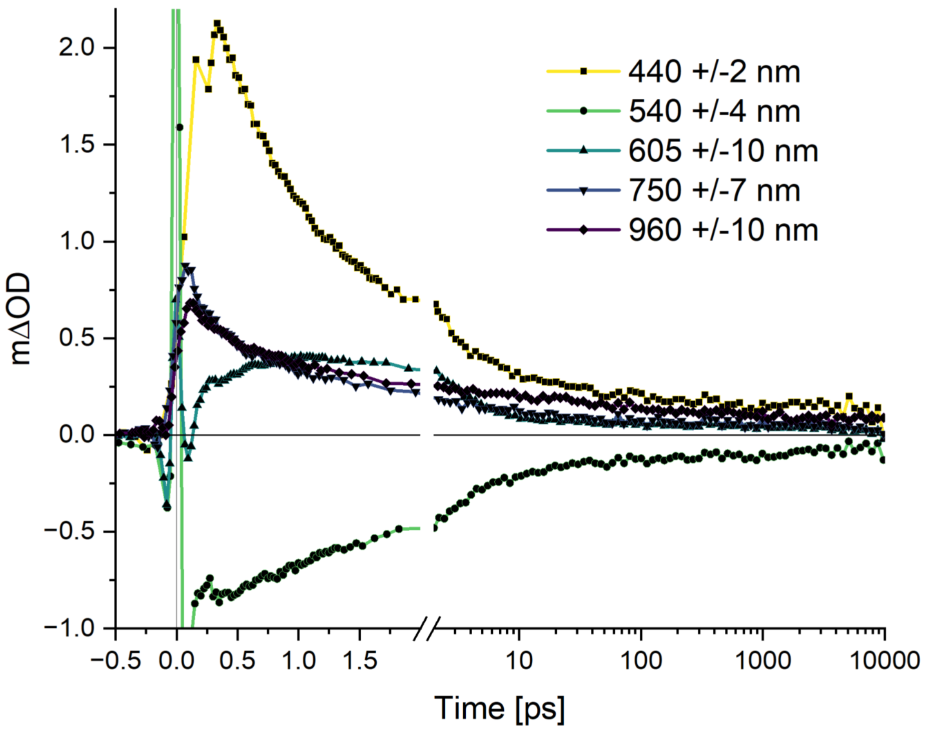

Figure S46. Measured kinetics for $[Re(dmpe)_3]^{2+}$ in deaerated solutions of acetonitrile with 90 vol% anisole at 20 °C at selected wavelengths (see color coding in insert). Excitation occurred at 540 nm. The kinetic trace of the GSB at 540 nm does not return to zero differential absorption at longer time scales indicating CE at this high quencher concentration. Comparing the amplitudes at 540 nm at 0.4 ps with that at 1 ns suggests that 10-15 % of the excited state population has not returned to the ground state, which would translate to the CE yield.

Determination of τ_{CS} and τ_{CR}

The isosbestic point at 630 nm observed in the TA spectra for $[Re(dmpe)_3]^{2+}$ in deaerated solutions of acetonitrile with 90 vol% anisole (Figure S45a) suggests that the rate of CR is slower than the rate of CS in the photocycle. The kinetic trace at 605 nm has not returned to zero differential absorption on the nanosecond time scale in the presence of 90 vol% anisole, which suggests that not the entire excited state population has returned to the ground state. This effect is presumably due to CE. Based on analogous arguments as for the photocycle in the presence of 80 vol% anisole (Figure S44), a triexponential decay function was thus used to fit the kinetic trace a 605 nm to allow for the presence of a long-lived decay component in the photocycle in the presence of 90 vol% anisole (Figure S47a). By doing so, a time component of 0.5 ps was found to be related to CS and a time component of 2.5 ps was found to be related to CR. To validate the 605 nm fit, the kinetic trace at 750 nm was likewise fitted with a triexponential fit function, but in this fit the time components were fixed to 0.5 ps, 2.5 ps and 486 ps. The resulting fit agrees very well with the experimental data at 750 nm (Figure S47b).

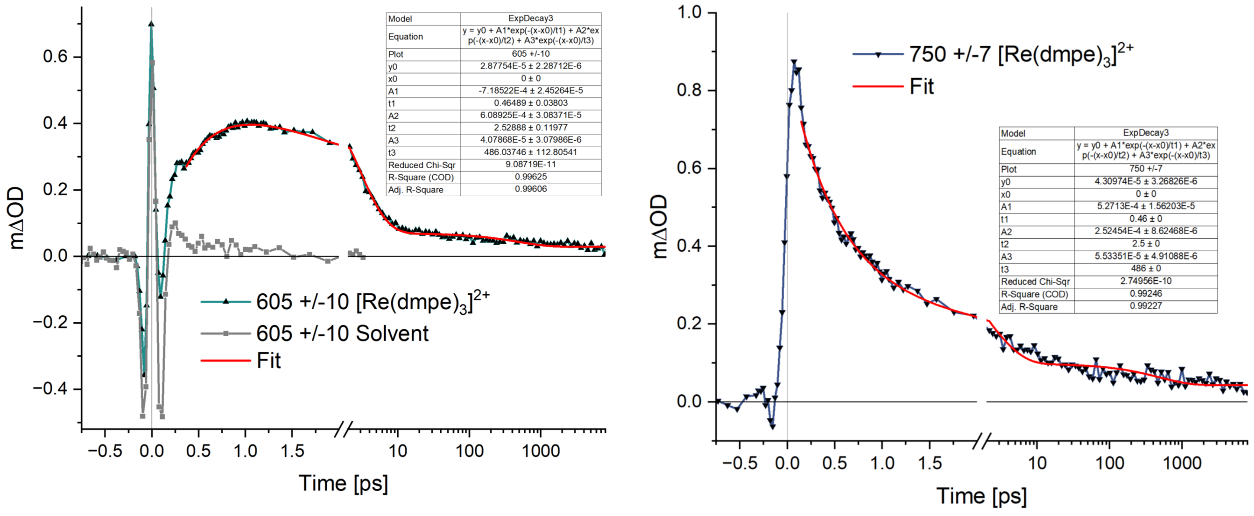

(a) (b)

Figure S47. Measured kinetics (symbols) and fit functions (red solid) at (a) 605 nm and (b) 750 nm of deaerated solutions of $[Re(dmpe)_3]^{2+}$ in acetonitrile with 90 vol% anisole at 20 °C. Solvent response at 605 nm (gray) is included to judge at what time point the dynamics are free of coherent artefacts at the ultrafast timescales. Excitation occurred at 540 nm.

Comparison of different anisole concentrations

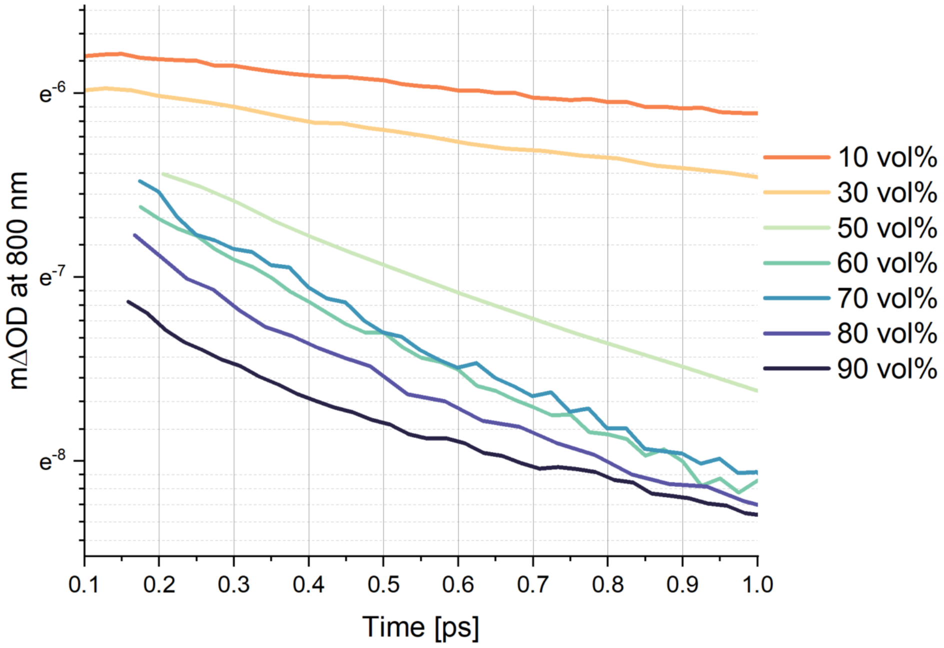

Figure S48. Measured kinetics at 800 nm of $[Re(dmpe)_3]^{2+}$ in deaerated solutions of acetonitrile with anisole at 20 °C. The concentration of anisole and the corresponding color coding are seen in the insert. Excitation occurred at 540 nm. The amplitude of the signal decreases as a function of anisole concentration at the earliest time scales indicating that an increased part of the excited state population has undergone CS.

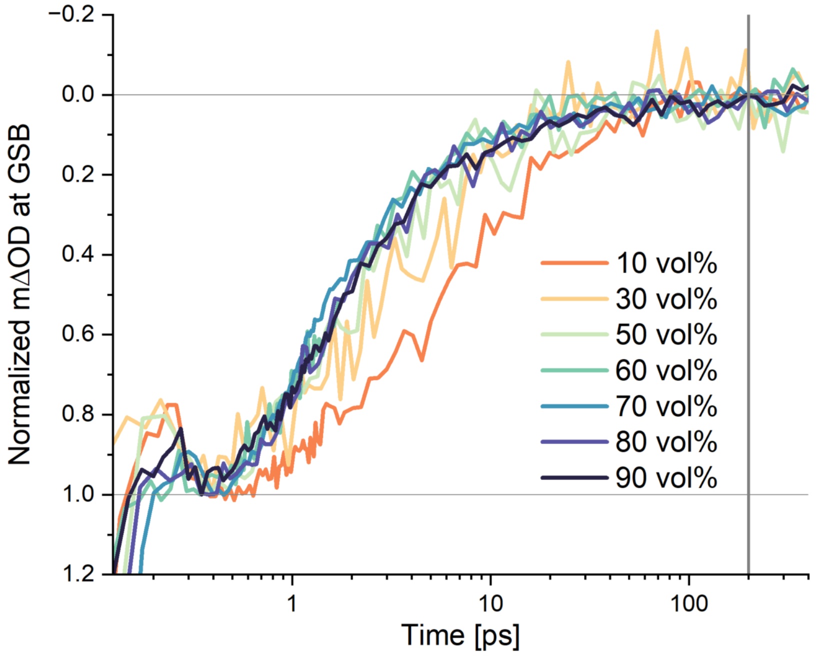

Figure S49. Normalized kinetic trace for the differential absorption at 540 nm (GSB) referenced to the delay time of 200 ps for $[Re(dmpe)_3]^{2+}$ in deaerated anisole/acetronitrile mixtures at 20 °C. The ground state recovery is associated to CR. The concentration of anisole in vol% and the corresponding color coding is seen in the insert.

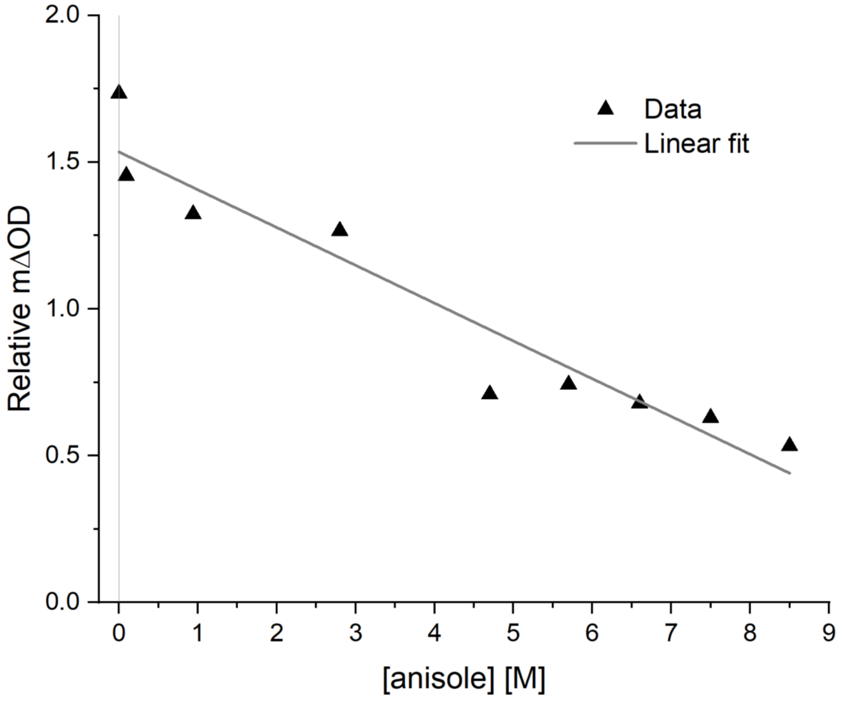

Figure S50. Relative ESA amplitude at 800 nm relative to the GSB for $[Re(dmpe)_3]^{2+}$ in deaerated solutions of acetonitrile with anisole at 20 °C. See spectra in Figure 3a. Excitation occurred at 540 nm.

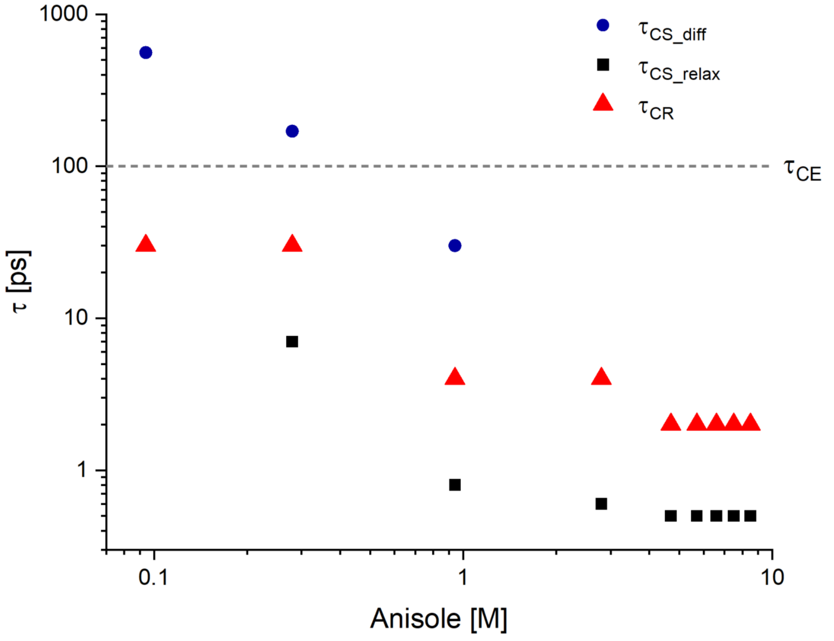

Figure S51. Anisole concentration dependence on diffusion-controlled CS (τ_{CS_diff}), close-contact CS (τ_{CS_relax}) and CR (τ_{CR}) lifetimes (Table 1). The dashed line indicates the typically estimated CE lifetime (τ_{CE}).[7-10]

8. Excited State Dynamics – Driving Force Dependence

Table S2: Singlet and triplet energies of investigated electron donors.[11] The ^{2}LMCT energy of $[Re(dmpe)_3]^{2+}$ is 2.2 eV.

	Singlet energy [eV]	Triplet energy [eV]
Benzene	4.8	3.9
Toluene	4.6	3.6
o-Xylene	4.5	3.7
m-Xylene	4.6	3.5
Mesitylene	4.5	2.8
Anisole	4.5	3.5

Benzene

Solutions of $[Re(dmpe)_3]^{2+}$ in acetonitrile containing 5.7 M benzene suffered from poor photostability. It was possible to obtain a reasonable spectral resolution by movement of the sample holder during the data collection (Figure S52), but the signal-to-noise ratio on the kinetic traces is very low (Figure S53). We therefore performed single wavelength TA spectroscopy at selected wavelengths, because the measurement time is shorter and excitation power is lower using this approach. This procedure resulted in an improved signal-to-noise ratio for the kinetic data (Figure S55).

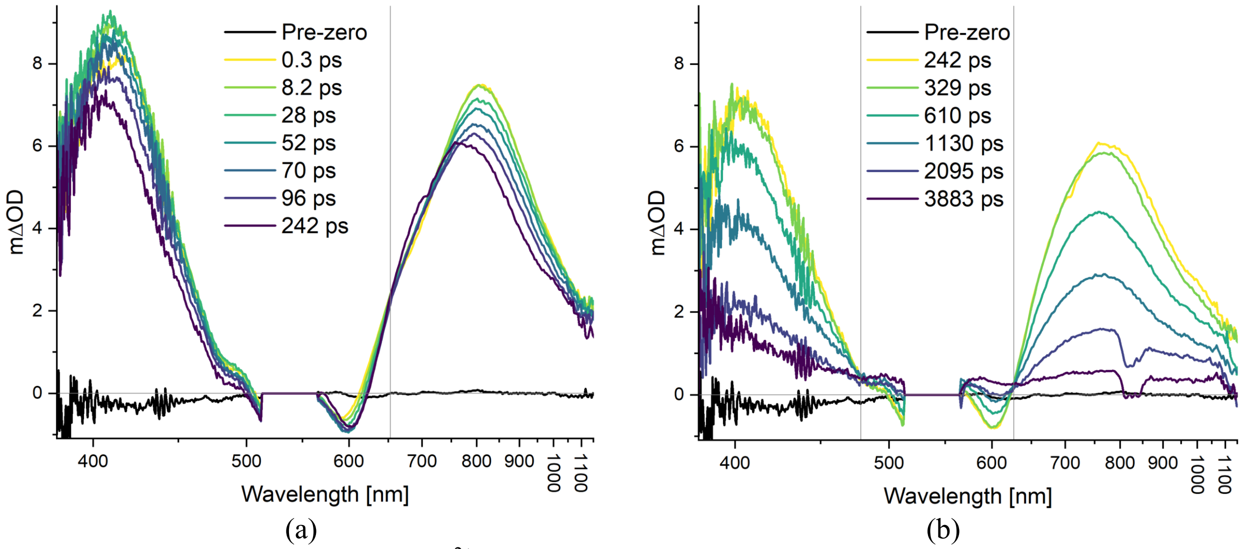

Figure S52. TA spectra of $[Re(dmpe)_3]^{2+}$ in a deaerated solution of 5.7 M benzene at (a) early and (b) later time scales. Delay times are given in picoseconds and their corresponding color codings are shown in the inserts. Gray vertical lines indicate isosbestic points. Excitation occurred at 540 nm. The region around the excitation wavelength was omitted due to significant scattering in this spectral range.

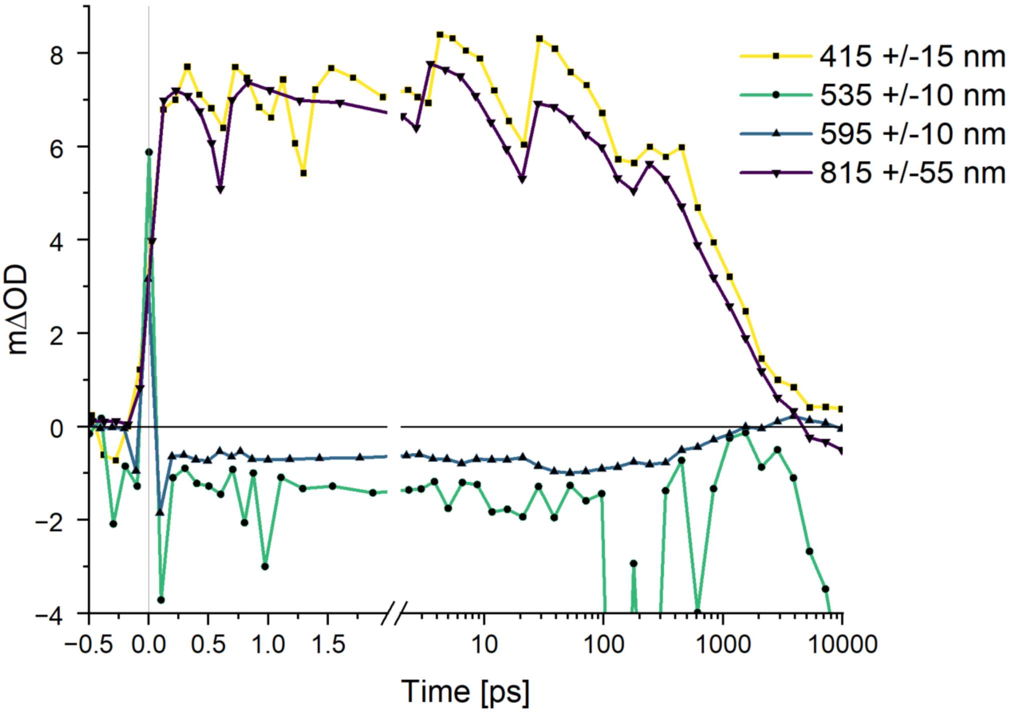

Figure S53. Measured kinetics for $[Re(dmpe)_3]^{2+}$ in deaerated solutions of 5.7 M benzene in acetonitrile at 20 °C at selected wavelengths (see color coding in insert). Excitation occurred at 540 nm.

<u>Global fit analysis</u>

The rate of CR is faster than the rate of CS for the photocycles of $[Re(dmpe)_3]^{2+}$ in deaerated solutions of 5.7 M benzene. Consequently, the TA data of $[Re(dmpe)_3]^{2+}$ in deaerated solutions of 5.7 M benzene in acetonitrile (Figure S52) was modelled with a two-step consecutive model:

$$\textbf{Photogenerated } ^2\textbf{LMCT} \xrightarrow{k_{Re,1}} \textbf{relaxed } ^2\textbf{LMCT} \xrightarrow{k_{CS,relax}} {}^2\textbf{GS}$$

where $k_{\text{Re},1}$ represents the rate constant caused by molecular transformations occurring on the picosecond timescale and $k_{\text{CS,relax}}$ is the rate of photoinduced electron transfer rate occurring from the relaxed ^{2}LMCT state. The rate constants were determined to $k_{Re,hot} = 5.6 \times 10^{-3}$ ps^{-1} and $k_{Re,relax} = 7.5 \times 10^{-4}$ ps^{-1}, translating to lifetimes of 180 ps and 1350 ns, respectively (Figure S54).

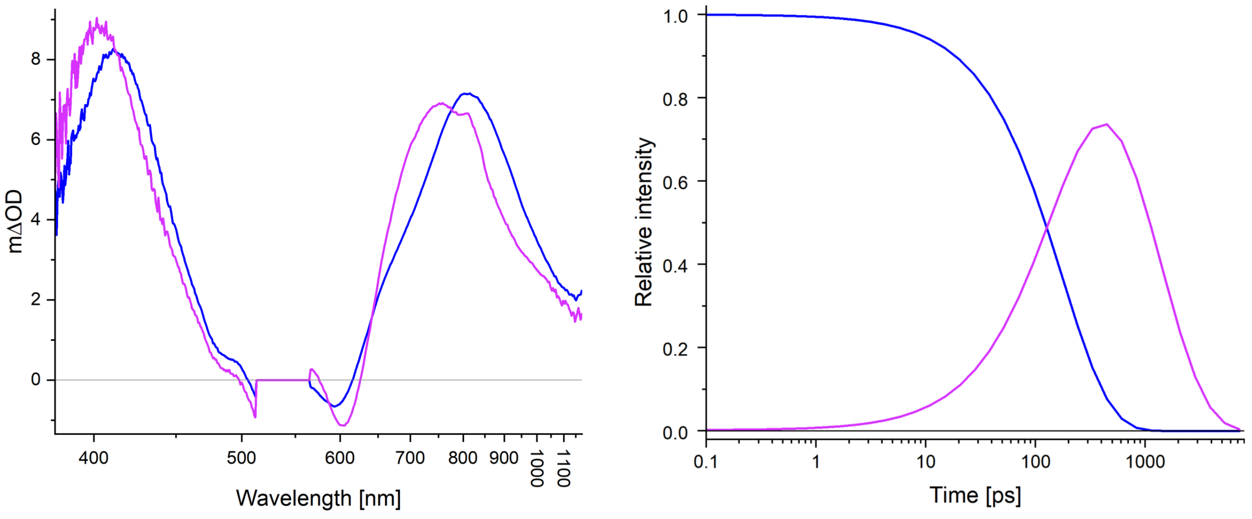

(a) (b)

Figure S54. (a) Species associated spectra associated with the photogenerated ^{2}LMCT state (blue) and the relaxed ^{2}LMCT state (pink) and the corresponding concentration profiles related to the excited state dynamics of $[Re(dmpe)_3]^{2+}$ in deaerated solutions of acetonitrile with 5.7 M benzene at 20 °C.

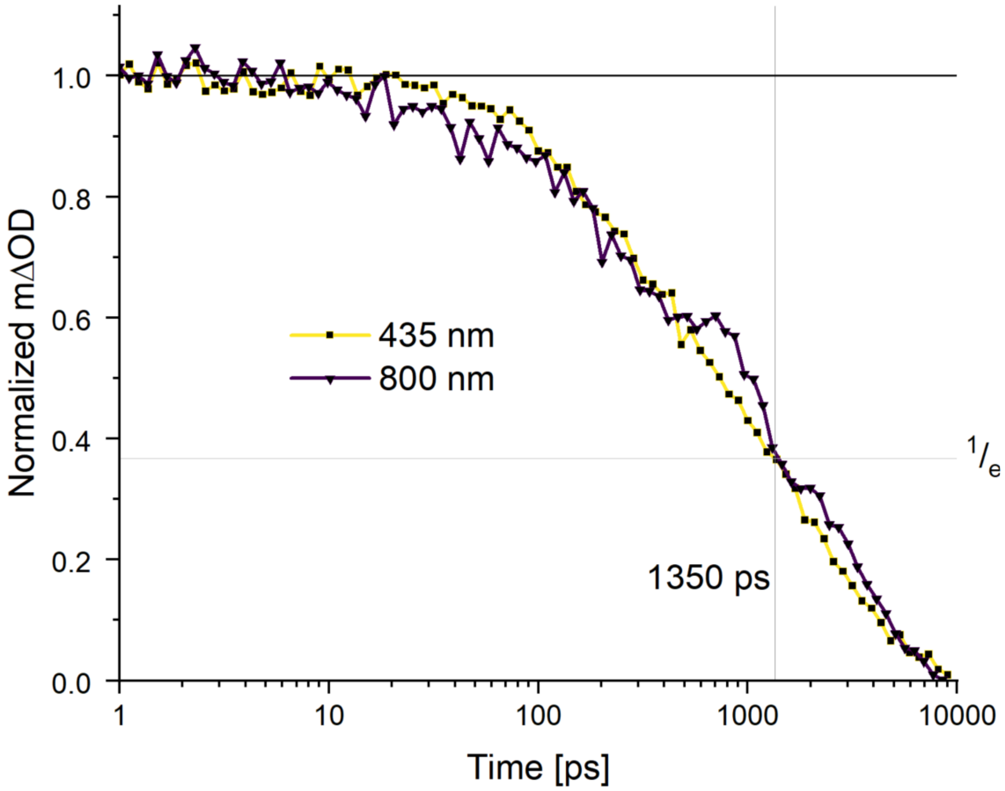

Figure S55. Measured kinetics of $[Re(dmpe)_3]^{2+}$ in a deaerated solution of 5.7 M benzene in acetonitrile at selected wavelengths (see color coding in insert). Excitation occurred at 535 nm. In agreement with the global fit analysis (Figure S54), the slow decay component was found to have a lifetime of 1350 ps.

Determination of τ_{CR}

With the same arguments as for the analysis of the TA data in the presence of 1 vol% anisole (Figure S22), the rate of CR was estimated to 20 ps in the photocycle of $[Re(dmpe)_3]^{2+}$ in deaerated solutions of acetonitrile with 5.7 M benzene (Figure S56).

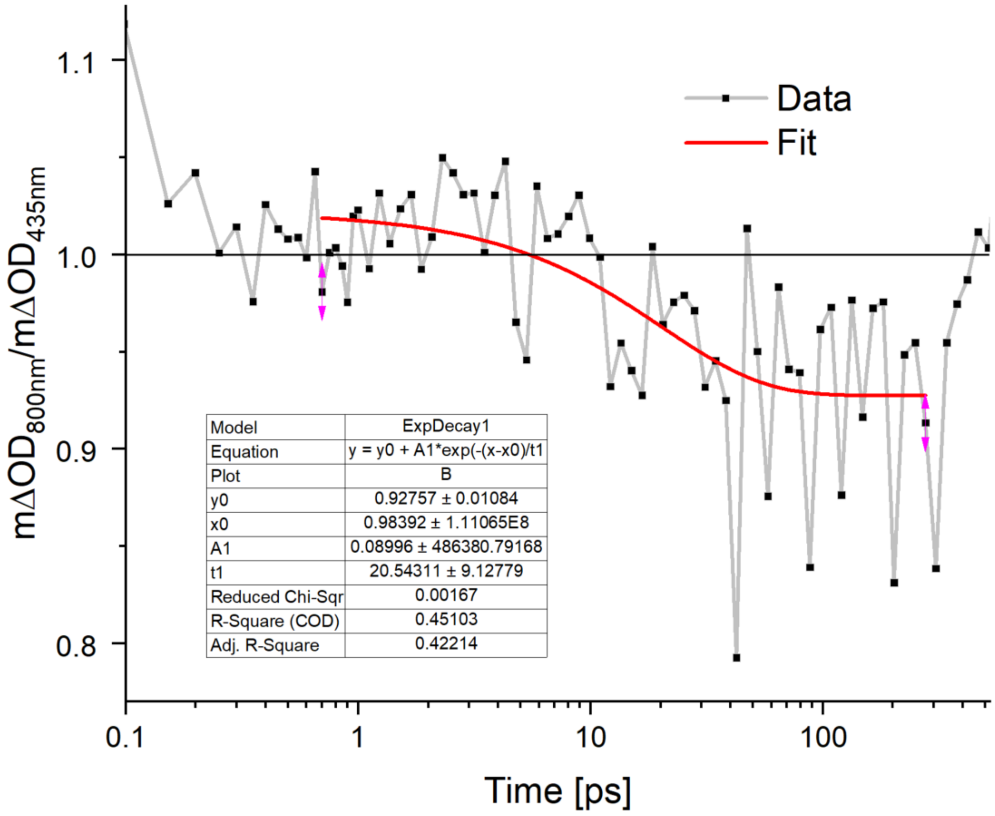

Figure S56. Divided normalized kinetics traces at 800 nm and 435 nm for $[Re(dmpe)_3]^{2+}$ in deaerated solutions of acetonitrile with 5.7 M benzene.

Toluene

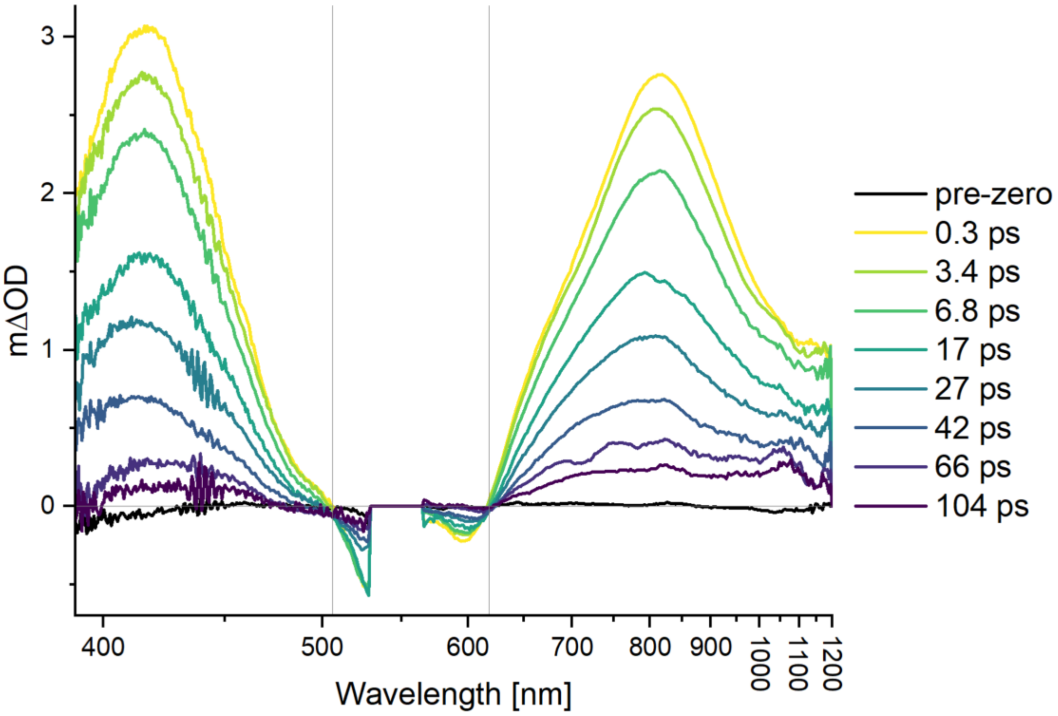

Figure S57. TA spectra of $[Re(dmpe)_3]^{2+}$ in a deaerated solution of 5.7 M toluene in acetonitrile. Delay times are given in picoseconds and their corresponding color codings are shown in the inserts. Gray vertical lines indicate isosbestic points. Excitation occurred at 540 nm. The region around the excitation wavelength was omitted due to significant scattering in this spectral range.

Solutions of $[Re(dmpe)_3]^{2+}$ in acetonitrile containing 5.7 M toluene suffered from poor photostability (Figure S58), however, better than when benzene was used as a quencher. The signal-to-noise ratio was improved by measuring single kinetic wavelengths at selected wavelengths, allowing for estimation of the time component related to CS (Figure S59) of 30 ps. Unfortunately, trustworthy determination of the recombination rate was not possible in this case.

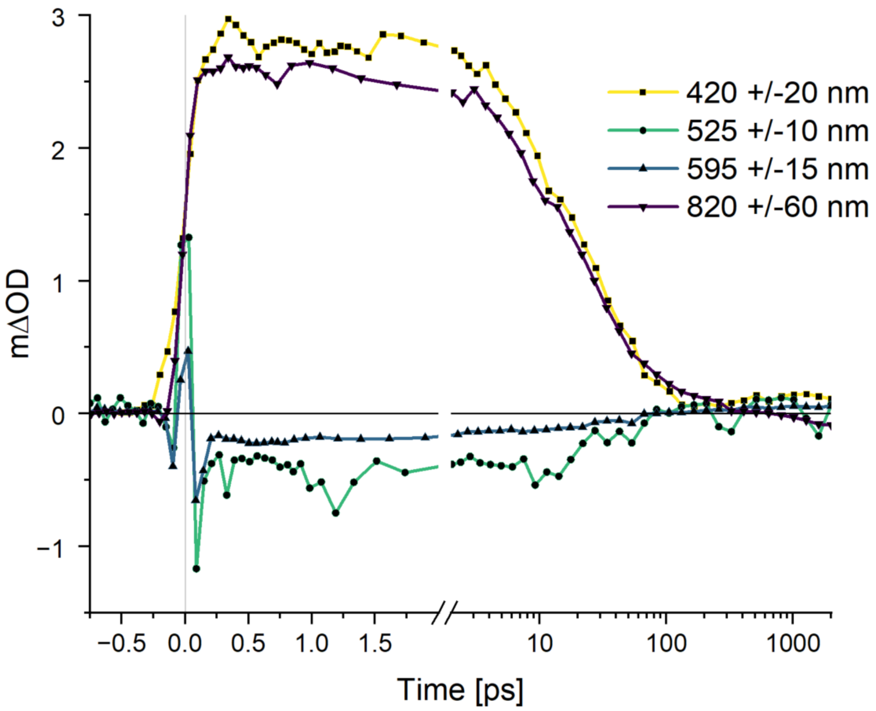

Figure S58. Measured kinetics for $[Re(dmpe)_3]^{2+}$ in deaerated solutions of 5.7 M toluene in acetonitrile at 20 °C at selected wavelengths (see color coding in insert). Excitation occurred at 540 nm.

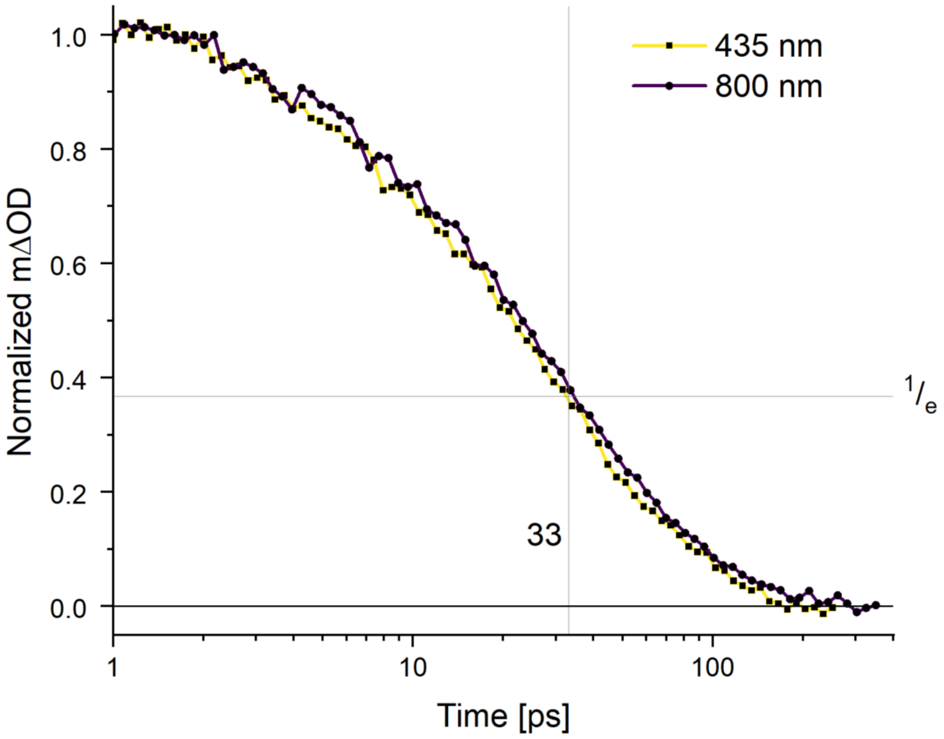

Figure S59. Measured kinetics for $[Re(dmpe)_3]^{2+}$ in deaerated solutions of 5.7 M toluene in acetonitrile at 20 °C at selected wavelengths (see color coding in insert). Excitation occurred at 535 nm.

o-Xylene

(a)

(b)

(c)

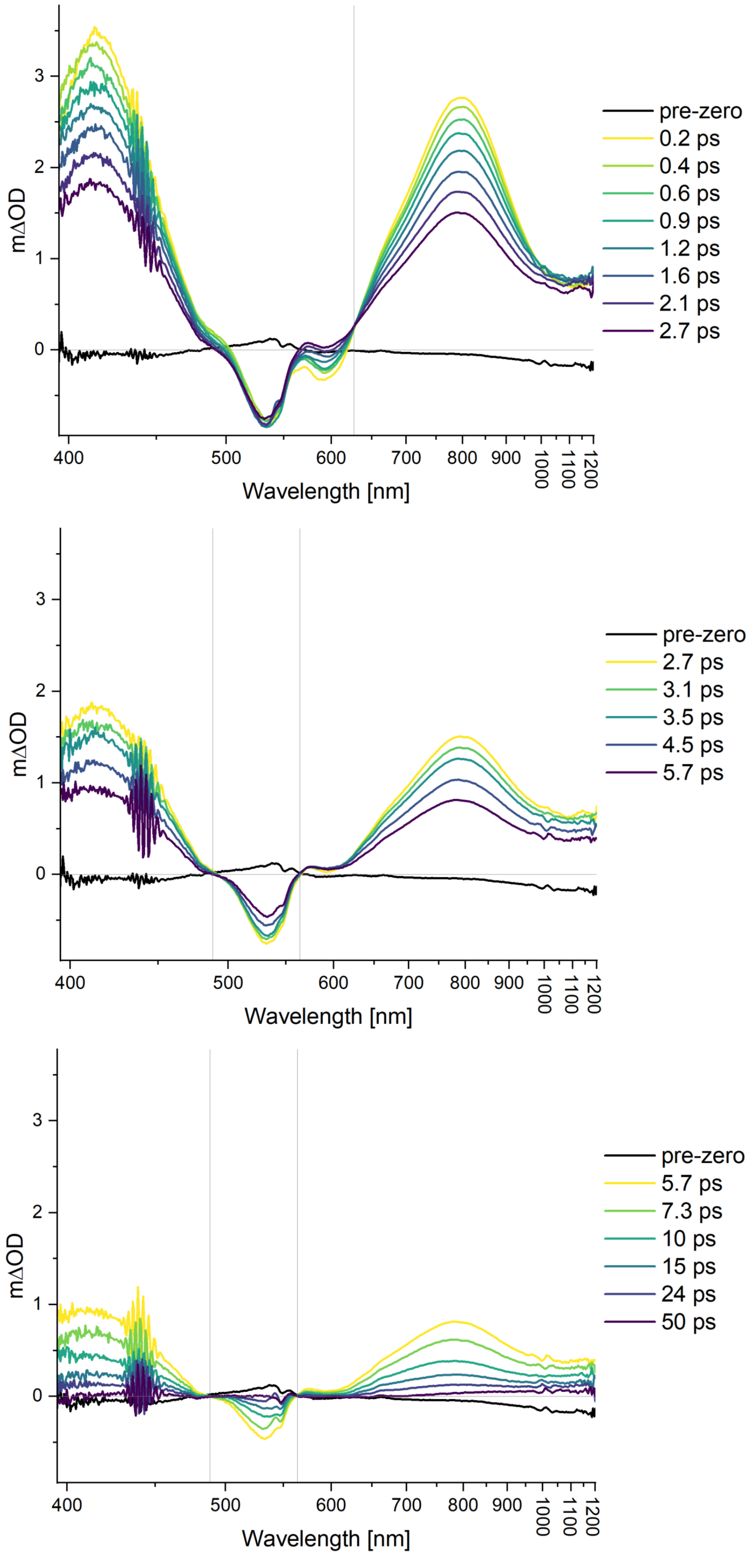

Figure S60. TA spectra of $[Re(dmpe)_3]^{2+}$ in a deaerated solution of 5.7 M o-xylene at (a) early, (b) intermediate and (c) later time scales. Delay times are given in picoseconds and their corresponding color codings are shown in the inserts. Gray vertical lines indicate isosbestic points. Excitation occurred at 540 nm.

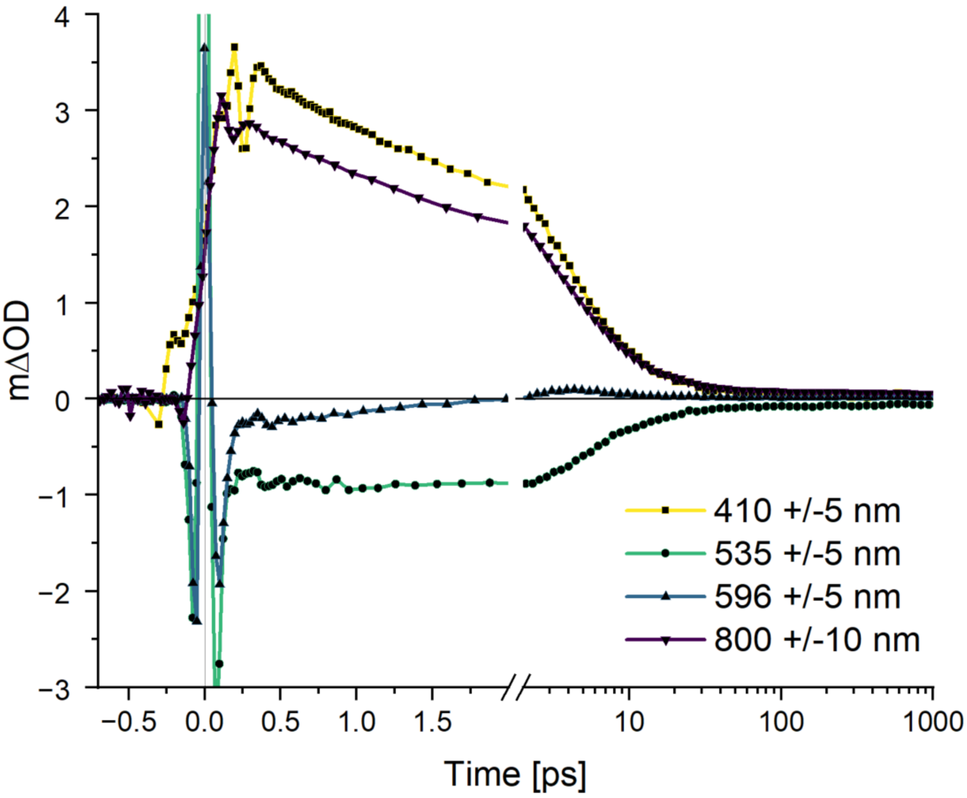

Figure S61. Measured kinetics for $[Re(dmpe)_3]^{2+}$ in deaerated solutions of 5.7 M *o*-xylene in acetonitrile at 20 °C at selected wavelengths (see color coding in insert). Excitation occurred at 540 nm. The kinetic trace of the GSB at 535 nm does not return to zero differential absorption at longer time scales indicating non-negligible CE. Comparing the GSB amplitude at sub-picosecond timescale with that at sub-nanosecond timescale suggests that CE accounts for 5-10%.

Determination of τ_{CS} and τ_{CR}

The isosbestic point at 630 nm observed in the TA spectra for $[Re(dmpe)_3]^{2+}$ in deaerated solutions of acetonitrile with 70 vol% *o*-xylene (Figure S60a) suggests that the rate of CR is slower than the rate of CS in the photocycle. The time component related to close-contact CS can be estimated from the growth of the spectral feature associated with the stimulated emission at 596 nm (Figure S62), whereas the subsequent disappearance of the 596 nm signal to zero differential absorption is related to the CR process. The time components will naturally also be present in kinetic traces at other key wavelengths, however, at 596 nm the two processes have conveniently opposite signs i.e. CS is related to *a growth*, whereas CR is related to *a decay*. In other words, there is a clearer separation of the individual contributions in the kinetics at 596 nm than at other wavelengths. The kinetic trace at 596 nm has not returned to zero differential absorption on the nanosecond time scale in the presence of 70 vol% *o*-xylene, which suggests that the entire excited state population has not returned to the ground state. This effect is presumably due to non-negligible CE. Using a triexponential fit function to fit the kinetics at 596 nm yields a fit that matches the data (green in Figure S62) but this fit does not describe the kinetics at 800 nm well. The time components for the photocycle of

$[Re(dmpe)_3]^{2+}$ with a quencher concentration of 70 vol% *o*-xylene were therefore estimated from the kinetic trace 800 nm (Figure S63). Using a biexponential fit function or a triexponential fit function resulted in fits with a good agreement between the 800 nm kinetic trace and the fit. However, using the output from the 800 nm fits as input for fitting the kinetic trace at 596 makes it clear that three time components (red in Figure S62) are required to get a good agreement between the data and the fit. The time components related to close-contact CS and CR are therefore estimated to 1.8 ps and 4.5 ps, respectively.

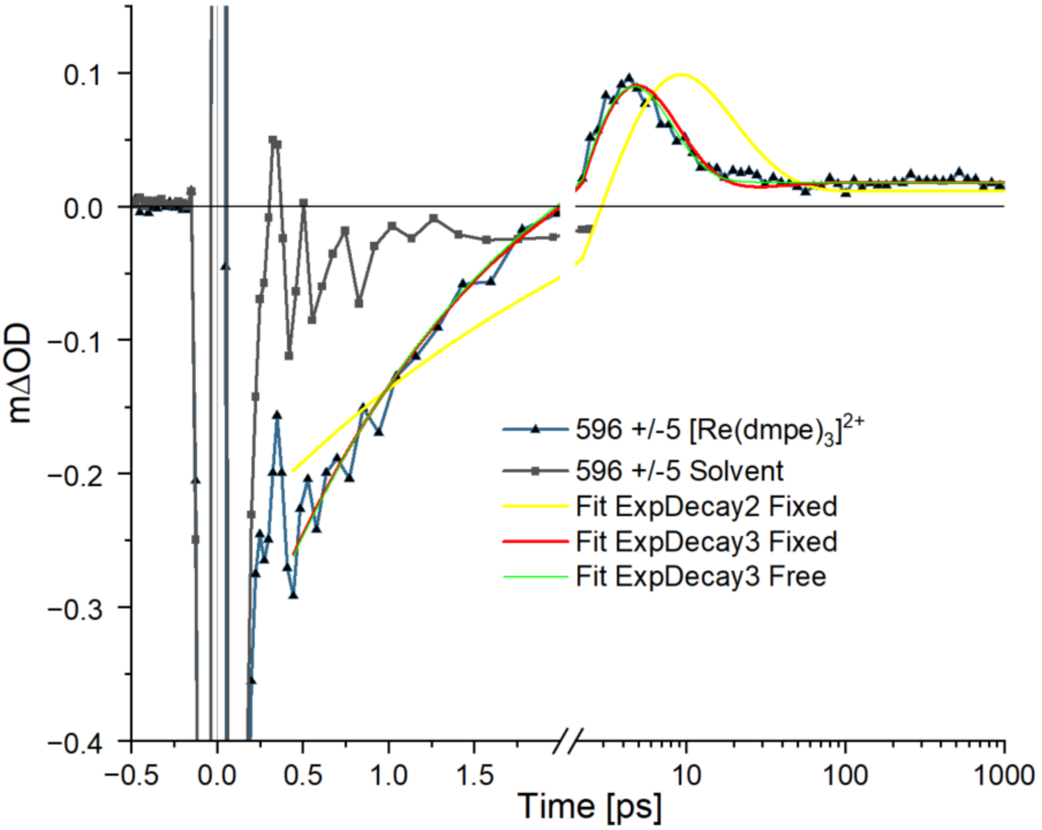

Fit ExpDecay3 Free

Model	ExpDecay3
Equation	y = y0 + A1*exp(-(x-x0)/t1) + A2*exp(-(x-x0)/t2) + A3*exp(-(x-x0)/t
Plot	596 +/-5
y0	1.81034E-5 ± 1.22902E-6
x0	0 ± 0
A1	-0.00259 ± 0.25943
t1	2.24128 ± 15.32697
A2	0.00117 ± --
t2	2.73133 ± 312.04581
A3	0.001 ± --
t3	2.74418 ± 192.83836
Reduced Chi-Sq	7.83679E-11
R-Square (COD)	0.98811
Adj. R-Square	0.98724

Fit ExpDecay2 Fixed

Model	ExpDecay2
Equation	y = y0 + A1*exp(-(x-x0)/t1) + A2*exp(-(x-x0)/t2)
Plot	596 +/-5
y0	1.12426E-5 ± 4.32417E-6
x0	0 ± 0
A1	-4.69416E-4 ± 2.55232E-5
t1	3 ± 0
A2	2.0172E-4 ± 1.85264E-5
t2	15 ± 0
Reduced Chi-Sq	9.44073E-10
R-Square (COD)	0.84979
Adj. R-Square	0.8463

Fit ExpDecay3 Fixed

Model	ExpDecay3
Equation	y = y0 + A1*exp(-(x-x0)/t1) + A2*exp(-(x-x0)/t2) + A3*exp(-(x-x0)
Plot	596 +/-5
y0	1.81966E-5 ± 1.35846E-6
x0	0 ± 0
A1	-8.09622E-4 ± 1.86909E-5
t1	1.8 ± 0
A2	4.05258E-4 ± 1.90353E-5
t2	4.5 ± 0
A3	-1.29223E-5 ± 6.53055E-6
t3	26 ± 0
Reduced Chi-Sq	8.35255E-11
R-Square (COD	0.98687
Adj. R-Square	0.9864

Figure S62. Measured kinetics (symbols) and fit functions (solid) at 596 nm and of deaerated solutions of $[Re(dmpe)_3]^{2+}$ in acetonitrile with 70 vol% *o*-xylene at 20 °C. Solvent response at 596 nm (gray) is included to judge at what time point the dynamics are free of coherent artefacts at the ultrafast timescales. Excitation occurred at 540 nm.

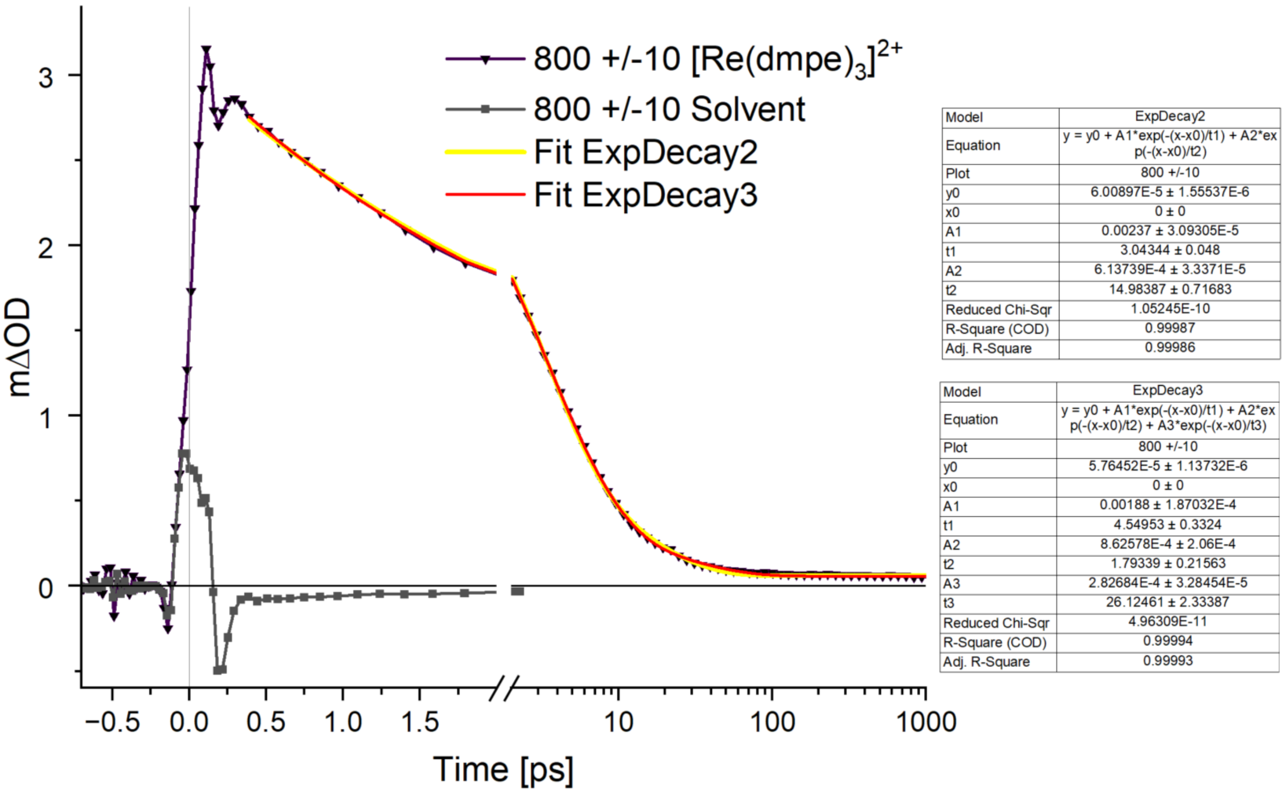

Model	ExpDecay2
Equation	y = y0 + A1*exp(-(x-x0)/t1) + A2*exp(-(x-x0)/t2)
Plot	800 +/-10
y0	6.00897E-5 ± 1.55537E-6
x0	0 ± 0
A1	0.00237 ± 3.09305E-5
t1	3.04344 ± 0.048
A2	6.13739E-4 ± 3.3371E-5
t2	14.98387 ± 0.71683
Reduced Chi-Sqr	1.05245E-10
R-Square (COD)	0.99987
Adj. R-Square	0.99986

Model	ExpDecay3
Equation	y = y0 + A1*exp(-(x-x0)/t1) + A2*exp(-(x-x0)/t2) + A3*exp(-(x-x0)/t3)
Plot	800 +/-10
y0	5.76452E-5 ± 1.13732E-6
x0	0 ± 0
A1	0.00188 ± 1.87032E-4
t1	4.54953 ± 0.3324
A2	8.62578E-4 ± 2.06E-4
t2	1.79339 ± 0.21563
A3	2.82684E-4 ± 3.28454E-5
t3	26.12461 ± 2.33387
Reduced Chi-Sqr	4.96309E-11
R-Square (COD)	0.99994
Adj. R-Square	0.99993

Figure S63. Measured kinetics (symbols) and fit functions (solid) at 800 nm and of deaerated solutions of $[Re(dmpe)_3]^{2+}$ in acetonitrile with 70 vol% *o*-xylene at 20 °C. Solvent response (gray) is included to judge at what time point the dynamics are free of artefacts at the ultrafast timescales. Excitation occurred at 540 nm.

m-Xylene

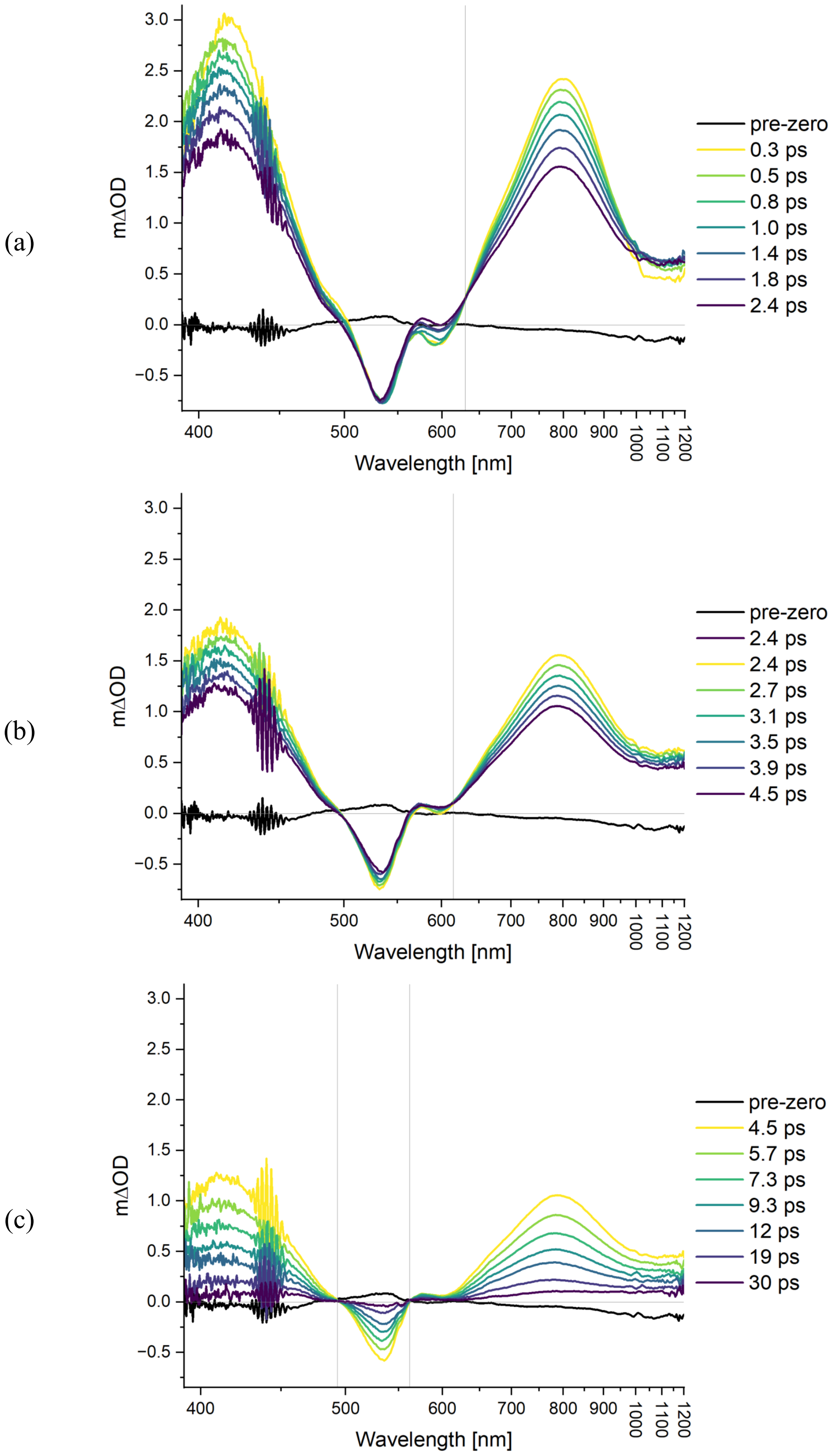

Figure S64. TA spectra of $[Re(dmpe)_3]^{2+}$ in a deaerated solution of 5.7 M *m*-xylene at (a) early, (b) intermediate and (c) later time scales. Delay times are given in picoseconds and their corresponding color codings are shown in the inserts. Gray vertical lines indicate isosbestic points. Excitation occurred at 540 nm.

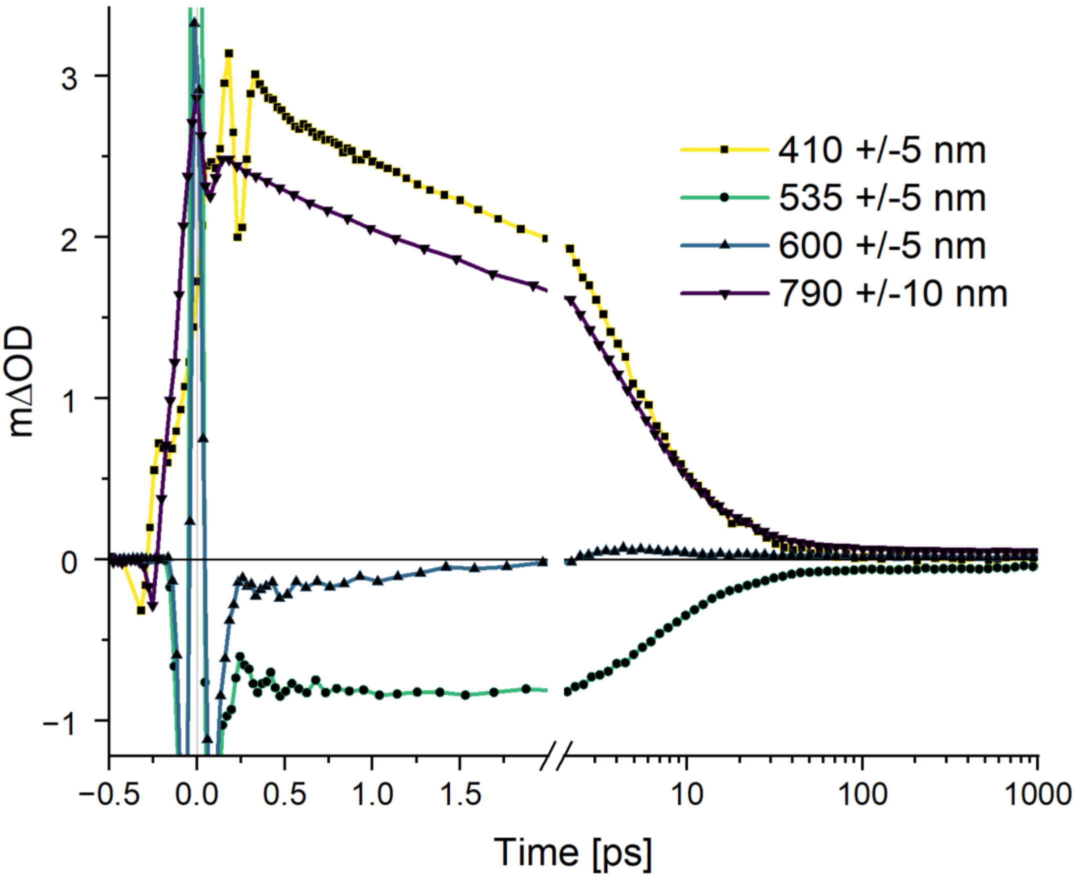

Figure S65. Measured kinetics for $[Re(dmpe)_3]^{2+}$ in deaerated solutions of 5.7 M *m*-xylene in acetonitrile at 20 °C at selected wavelengths (see color coding in insert). Excitation occurred at 540 nm. The kinetic trace of the GSB at 535 nm does not return to zero differential absorption at longer time scales indicating non-negligible CE. Comparing the GSB amplitude at sub-picosecond timescale with that at sub-nanosecond timescale suggests that CE accounts for 5-10%.

Determination of τ_{CS} and τ_{CR}

The isosbestic point at 630 nm observed in the TA spectra for $[Re(dmpe)_3]^{2+}$ in deaerated solutions of acetonitrile with 70 vol% *m*-xylene (Figure S64a) suggests that the rate of CR is slower than the rate of CS in the photocycle. Using the same arguments as for photocycles containing 70 vol% *o*-xylene (Figures S62 and S63), the kinetic trace at 596 nm was first fitted with a triexponential fit function, however, the result does, not support well the dynamics at 790 nm (green in Figures S66 and S67). The kinetic trace at 600 nm was thus fitted with a biexponential fit function to yield a time component for CS of 2.0 ps and for CR of 3.7 ps (red in Figure S66). Using these time components as input for fitting the kinetic trace at 790 nm (red in Figure S67) made it clear that a long-lived component is additionally required to obtain good agreement between the data at 790 nm and the fit (yellow in Figure S67). Including a long-lived time component at 600 nm did not alter the fit significantly (compare red and yellow in Figure S66).

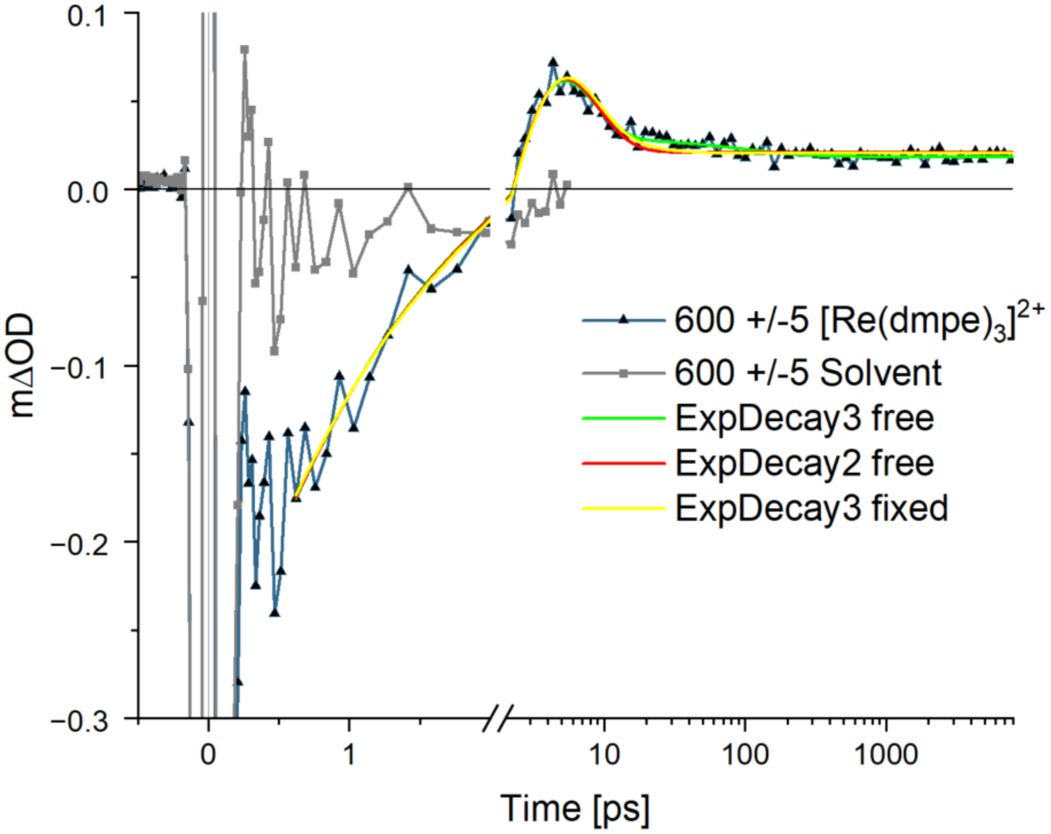

ExpDecay3 free

Model	ExpDecay3
Equation	y = y0 + A1*exp(-(x-x0)/t1) + A2*exp(-(x-x0)/t2) + A3*exp(-(x-x0)/t3)
Plot	598.06714
y0	1.85261E-5 ± 1.37796E-6
x0	0 ± 0
A1	-0.00873 ± 3.59814
t1	2.40725 ± 16.74216
A2	0.00839 ± 3.59815
t2	2.4895 ± 17.87714
A3	1.07593E-5 ± 4.79396E-6
t3	106.02816 ± 95.7595
Reduced Chi-Sqr	5.39762E-11
R-Square (COD)	0.98032
Adj. R-Square	0.97881

ExpDecay2 free

Model	ExpDecay2
Equation	y = y0 + A1*exp(-(x-x0)/t1) + A2*exp(-(x-x0)/t2)
Plot	598.06714
y0	2.09662E-5 ± 1.06904E-6
x0	0 ± 0
A1	-7.07315E-4 ± 4.80533E-4
t1	1.9962 ± 0.5087
A2	3.81296E-4 ± 4.89801E-4
t2	3.70711 ± 1.39639
Reduced Chi-Sqr	6.04425E-11
R-Square (COD)	0.9774
Adj. R-Square	0.97627

ExpDecay3 fixed

Model	ExpDecay3
Equation	y = y0 + A1*exp(-(x-x0)/t1) + A2*exp(-(x-x0)/t2) + A3*exp(-(x-x0)/t3)
Plot	598.06714
y0	2.02782E-5 ± 1.10208E-6
x0	0 ± 0
A1	-6.80579E-4 ± 2.84259E-5
t1	2 ± 0
A2	3.49095E-4 ± 2.88341E-5
t2	3.7 ± 0
A3	1.03712E-5 ± 7.28037E-6
t3	17 ± 0
Reduced Chi-Sqr	5.82373E-11
R-Square (COD)	0.97795
Adj. R-Square	0.97714

Figure S66. Measured kinetics (symbols) and fit functions (solid) at 600 nm and of deaerated solutions of $[Re(dmpe)_3]^{2+}$ in acetonitrile with 70 vol% *m*-xylene at 20 °C. Solvent response at 600 nm (gray) is included to judge at what time point the dynamics are free of coherent artefacts at the ultrafast timescales. Excitation occurred at 540 nm.

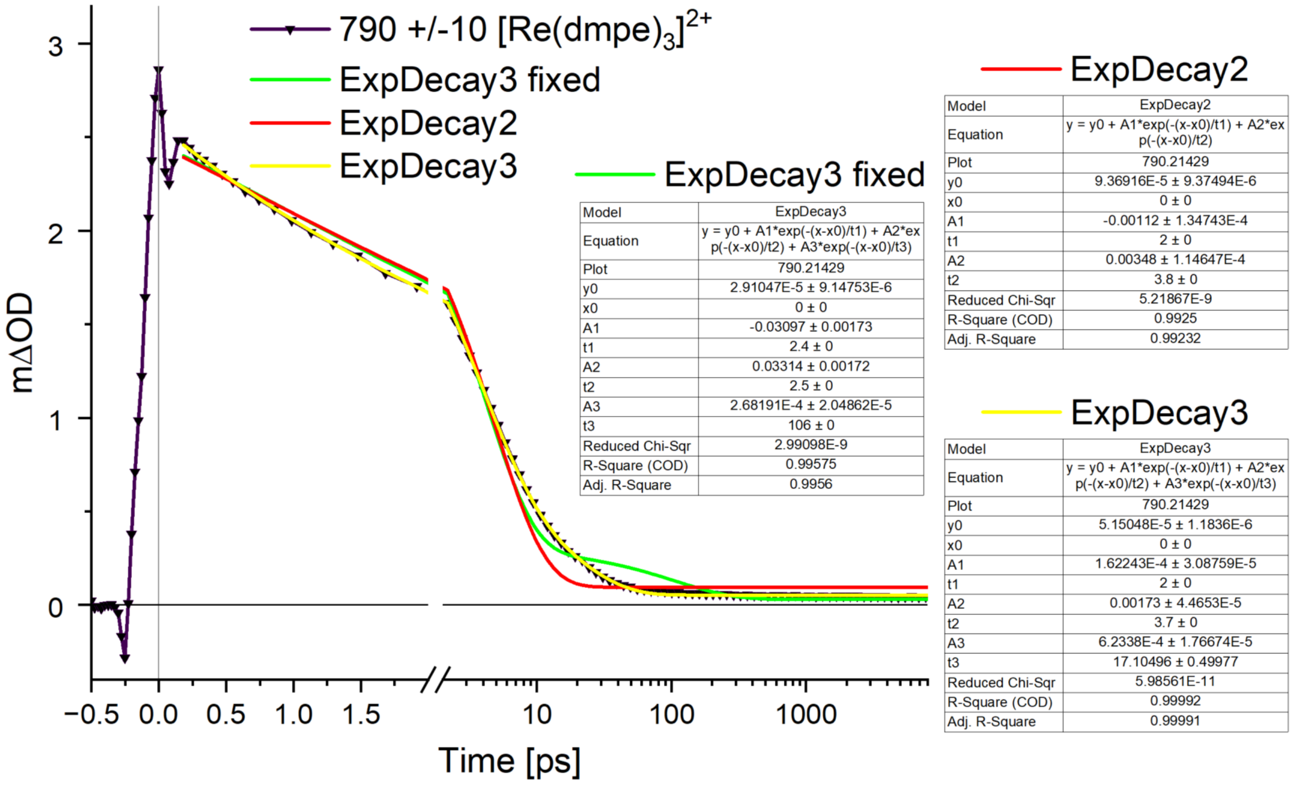

Figure S67. Measured kinetics (symbols) and fit functions (solid) at 800 nm and of deaerated solutions of $[Re(dmpe)_3]^{2+}$ in acetonitrile with 70 vol% *m*-xylene at 20 °C. Solvent response (gray) is included to judge at what time point the dynamics are free of coherent artefacts at the ultrafast timescales. Excitation occurred at 540 nm.

<u>Mesitylene</u>

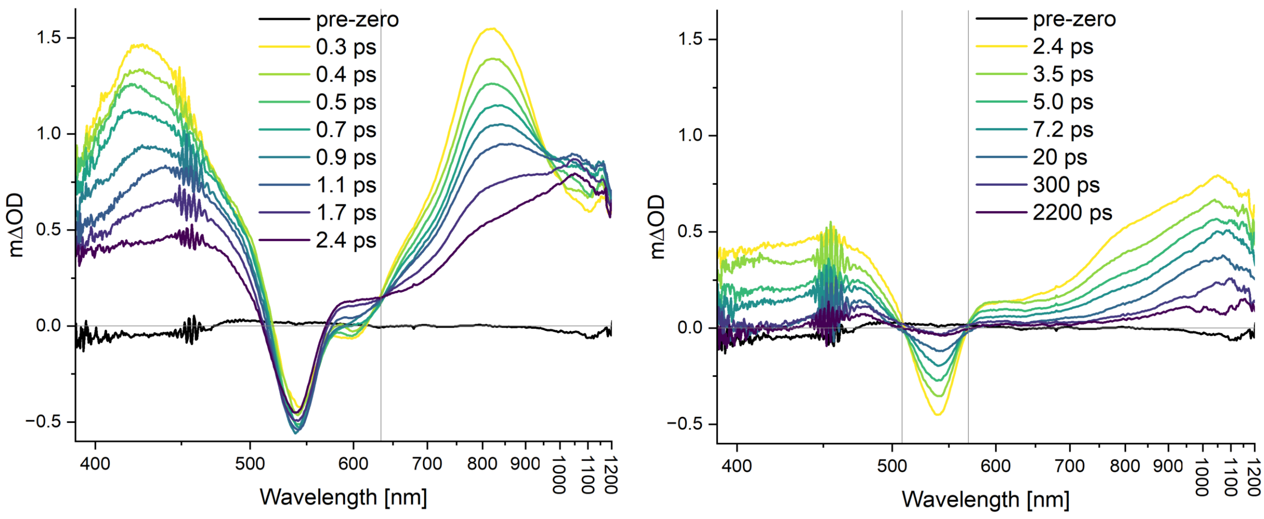

(a) (b)

Figure S68. TA spectra of $[Re(dmpe)_3]^{2+}$ in deaerated solutions of acetonitrile with 5.7 M mesitylene at 20 °C at (a) early and (b) later time scales. Delay times between pump and probe and their corresponding color coding are noted in ps in the inserts. Excitation occurred at 540 nm. Gray vertical lines indicate isosbestic points.

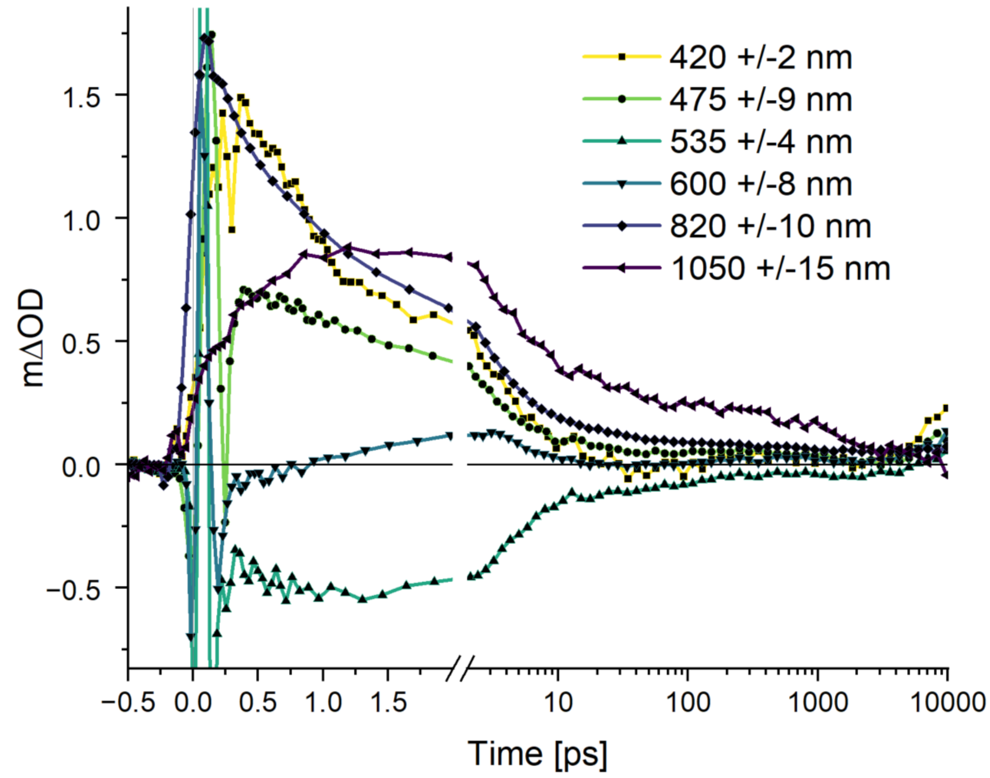

Figure S69. Measured kinetics for $[Re(dmpe)_3]^{2+}$ in deaerated solutions of 5.7 M mesitylene in acetonitrile at 20 °C at selected wavelengths (see color coding in insert). Excitation occurred at 540 nm. The kinetic trace at 1050 nm indicates the formation of mesitylene radical cation dimers.[5] CE and the influence of this dimerization process are currently under further investigation. Comparing the GSB amplitude at sub-picosecond timescale with that at sub-nanosecond timescale suggests that CE accounts for 5-10%.

Determination of τ_{CS} and τ_{CR}

The isosbestic point at 630 nm observed in the TA spectra for $[Re(dmpe)_3]^{2+}$ in deaerated solutions of acetonitrile with 80 vol% mesitylene (Figure S68a) suggests that the rate of CR is slower than the rate of CS in the photocycle. Using the same arguments as for photocycles containing 70 vol% *o*-xylene (Figures S62 and S63), the time components of CS and CR were estimated from fitting the kinetic trace at 600 nm with a biexponential fit function yielding 1.1 ps and 3.9 ps, respectively. Using these time components as input for fitting the kinetic trace at 820 nm made it clear that a long-lived component is additionally required to obtain good agreement between the data at 600 nm and the fit. Including a long-lived time component at 600 nm did not alter the fit significantly.

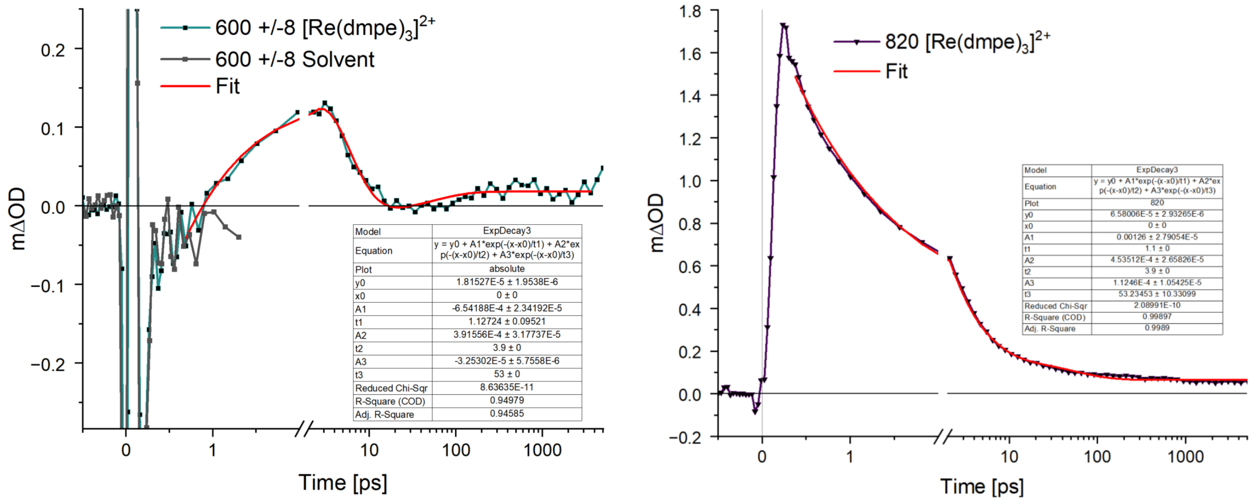

(a) (b)

Figure S70. Measured kinetics (symbols) and fit functions (red solid) at (a) 605 nm and (b) 750 nm of deaerated solutions of $[Re(dmpe)_3]^{2+}$ in acetonitrile with 80 vol% mesitylene at 20 °C. Solvent response at 600 nm (gray) is included to judge at what time point the dynamics are free of coherent artefacts at the ultrafast timescales. Excitation occurred at 540 nm.

Comparison of CS decays induced by different quenchers

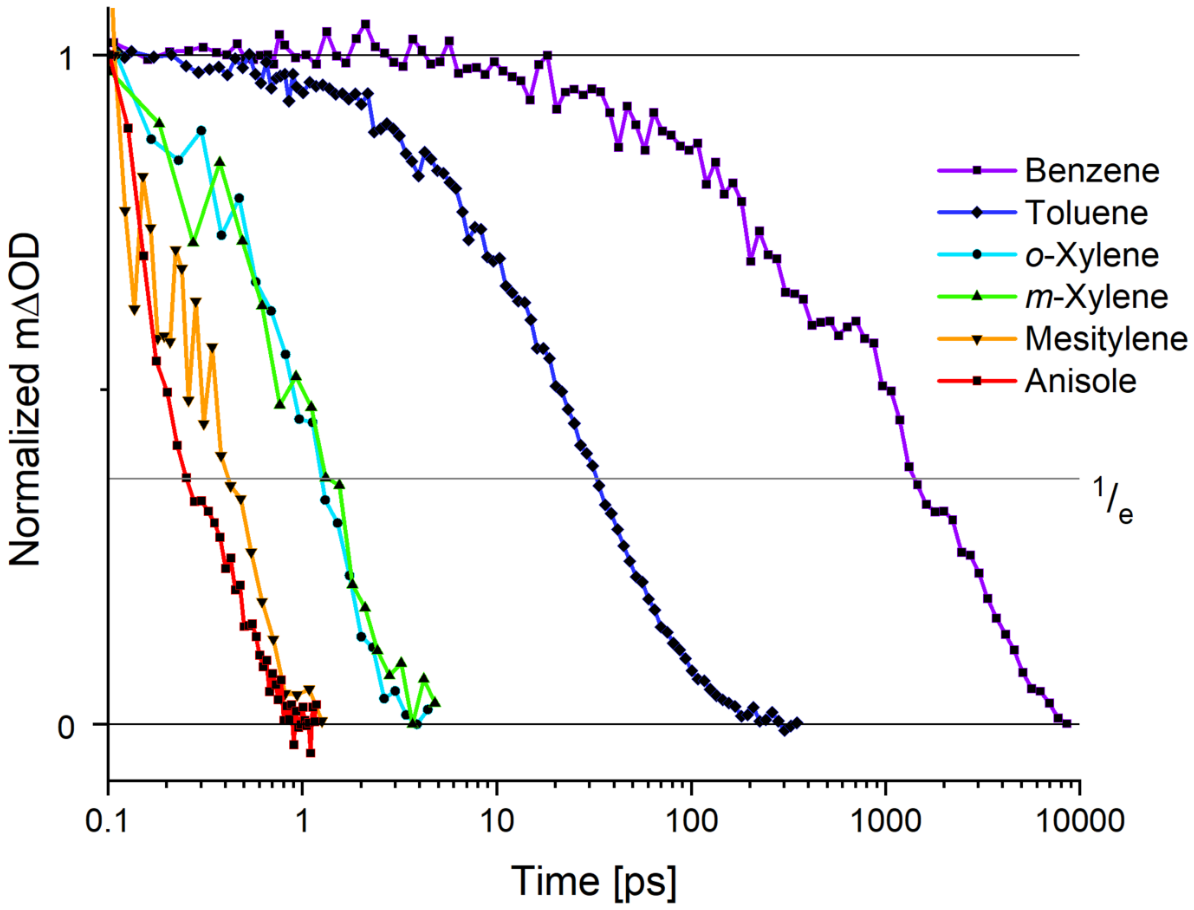

Figure S71. Kinetic trace associated with CS in the photocycle of $[Re(dmpe)_3]^{2+}$. The quencher and the corresponding color coding is seen in the insert.

9. Analysis using Marcus-Jortner-Levich theory

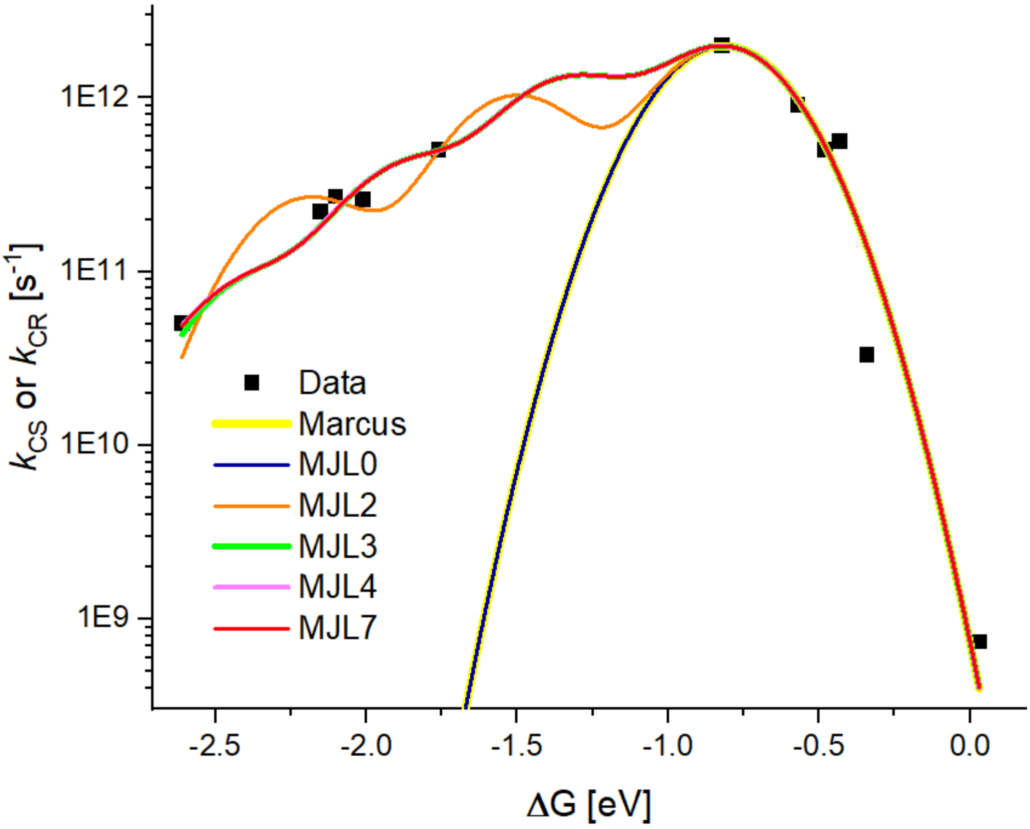

Figure S72. Marcus-Jortner-Levich (MJL) fits of the rates of CS and CR (squares) in the photocycle of $[Re(dmpe)_3]^{2+}$, where the number of higher-lying vibrational modes is varied from zero (classic Marcus, eq. 2) to seven (MJL7) in the model (eq. 3)

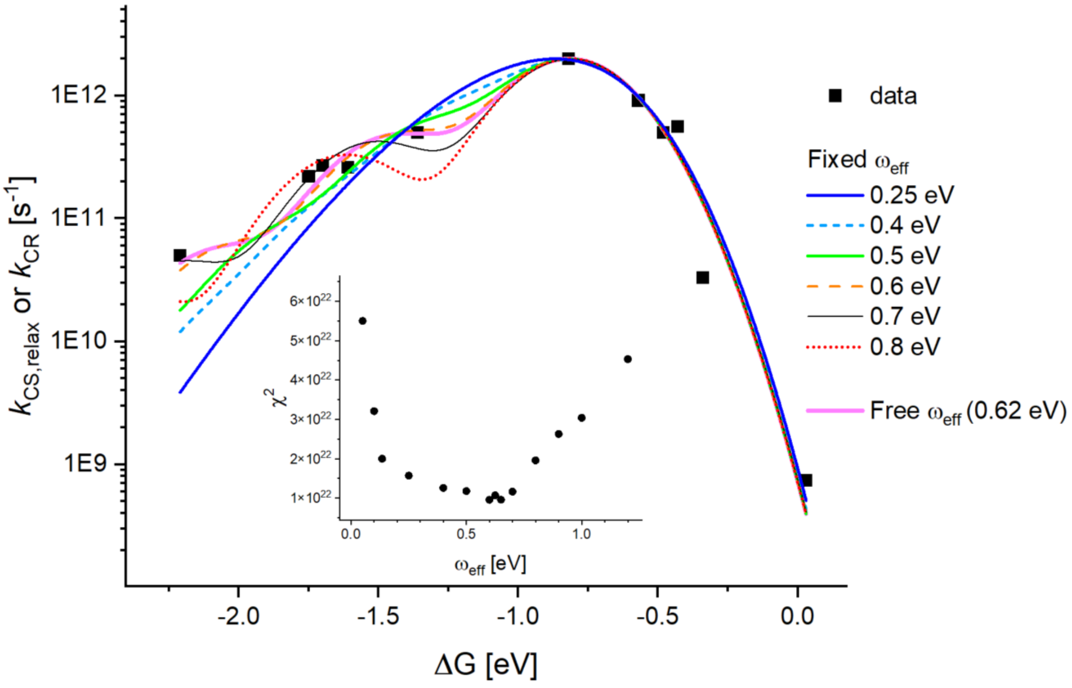

Figure S73. Marcus-Jortner-Levich fits (eq. 3) of the rates of CS and CR (squares) in the photocycle of $[Re(dmpe)_3]^{2+}$, where the vibrational mode (ω_{eff}) is optimized including seven higher-lying vibrational modes. Single effective vibrational modes lower than 0.25 eV poorly describes our data. This also means that the commonly used value of 1500 cm^{-1} (0.187 eV) is not applicable to our data.

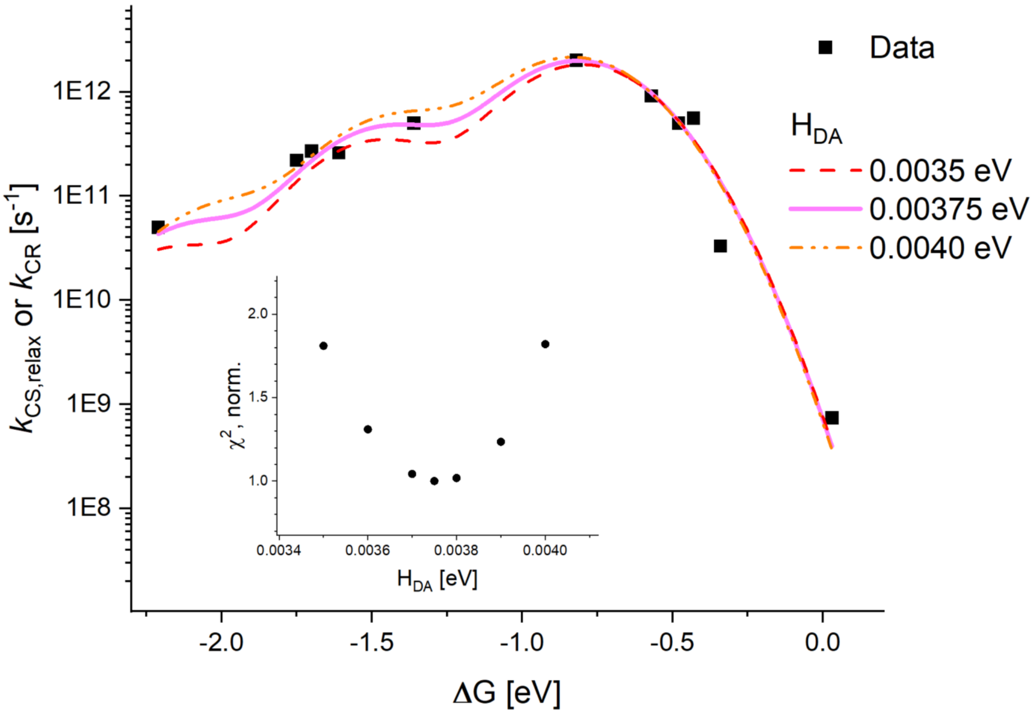

Figure S74. Marcus-Jortner-Levich fits (eq. 3) of the rates of CS and CR (squares) in the photocycle of $[Re(dmpe)_3]^{2+}$, where the electronic coupling (H_{DA}) is optimized.

10. Photostability studies

Significant photodegradation is observed in pure acetonitrile both in our single wavelength fs TA and our broadband fs TA measurements. This issue is minimized for the data recorded on the broadband fs TA setup as this setup provides a movable sample holder. This allows the cuvette to move during data collection so that a fresh unirradiated point of the cuvette can be exposed to the pump in between each scan (we collect multiple scans to improve the signal-to-noise ratio). Our single wavelength fs TA setup does, however, not allow for this movable approach and the cuvette is fixed throughout the measurement, hence the problem of photodegradation seems more significant in these single wavelength fs TA measurements. This is also why we cannot perform meaningful studies in pure acetonitrile using the single wavelength fs TA setup.

Importantly, the photodegradation does not lead to new absorption band(s) of the photoproduct in the excitation spectral region (Figure S75), meaning that we are not exciting the photoproduct. As a result, the TA signal will not change with time and what we measure is an average signal, but no other dynamics will be seen in or contribute to the TA spectra.

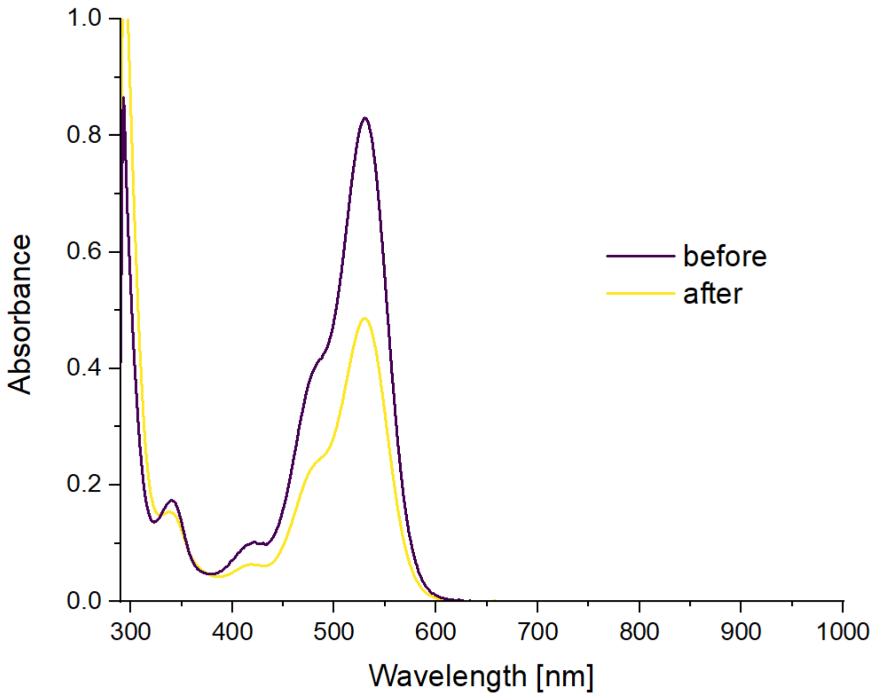

Figure S75. Absorption spectra of $[Re(dmpe)_3]^{2+}$ in deaerted 60% anisole in acetonitrile before and after the TA measurement.

In a comparable study (Figure S76), we evaluate the photostability of $[Re(dmpe)_3]^{2+}$ in the absence and presence of anisole by using a 470 nm fiber-coupled LED (ThorLabs, M470F4). While these irradiation conditions – using an LED as irradiation source – are not directly representative to those used in the TA

measurements, this nevertheless allows for a direct comparison of photostability in the presence and absence of anisole.

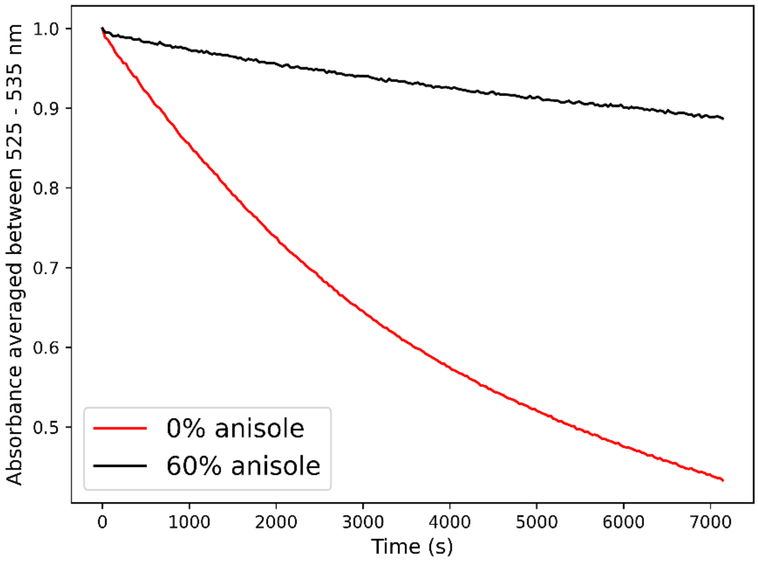

Figure S76. The photostability of $[Re(dmpe)_3]^{2+}$ in deaerated solutions of acetonitrile with 0 vol% (red) and 60 vol% (black) anisole is monitored by the intensity of the ^{2}LMCT absorption band at 530 nm as a function of time upon irradiation at 470 nm. The initial optical density was the same at 470 nm.

11. Absorption spectrum of the anisole radical cations

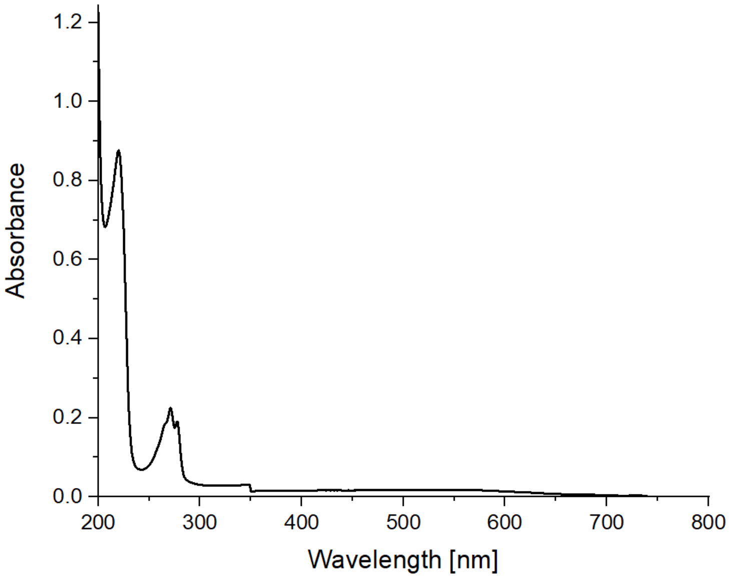

Figure S77. Absorption spectrum of anisole in acetonitrile.

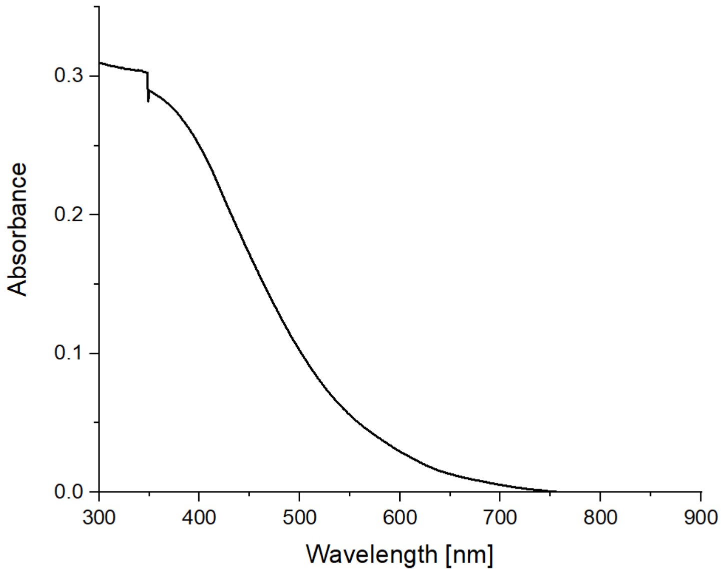

Figure S78. Spectroelectrochemically-generated absorption spectrum of the anisole radical cation obtained by applying a potential of +1.9 V vs. SCE to a solution of anisole in acetonitrile for 10 min. Structurally similar aromatic radical cations such as the benzene and toluene radical cations also absorb between 350 nm and 450 nm.[5, 6]

Applying +1.9 V vs. SCE to a solution of anisole in acetonitrile results in an orange solution with the absorption spectrum seen in Figure S78. Over time the solution becomes cloudy and for this reason we do not estimate an extinction coefficient. Aromatic radical cations are prone to form radical cation dimers[5, 6], so it seems likely that the cloudiness originates from dimerization products.

12. References

1 G. F. Ciani, G. D'Alfonso, P. F. Romiti, A. Sironi and M. Freni, *Inorg. Chim. Acta*, 1983, **72**, 29-37.

2 F. Wu, C. Deraedt, Y. Cornaton, L. Ruhlmann, L. Karmazin, C. Bailly, N. Kyritsakas, N. Le Breton, S. Choua and J.-P. Djukic, *Organometallics*, 2021, **40**, 2624-2642.

3 E. Bill, *(Max-Planck-Institute for Chemical Energy Conversion in Mülheim). Eview4wr.*, 2018.

4 J. J. Adams, N. Arulsamy, B. P. Sullivan, D. M. Roddick, A. Neuberger and R. H. Schmehl, *Inorg. Chem.*, 2015, **54**, 11136-11149.

5 B. Badger and B. Brocklehurst, *Trans. Faraday Soc.*, 1969, **65**, 2582-2587.

6 T. N. Das, *J. Phys. Chem. A*, 2009, **113**, 6489-6493.

7 H. J. Wolff, D. Burssher and U. E. Steiner, *Pure Appl. Chem.*, 1995, **67**, 167-174.

8 K. A. Hötzer, A. Klingert, T. Klumpp, E. Krissinel, D. Bürssner and U. E. Steiner, *J. Phys. Chem. A*, 2002, **106**, 2207-2217.

9 L. Fodor, A. Horváth, K. A. Hötzer, S. Walbert and U. E. Steiner, *Chem. Phys. Lett.*, 2000, **316**, 411-418.

10 P. Gilch, M. Linsenmann, W. Haas and U. E. Steiner, *Chem. Phys. Lett.*, 1996, **254**, 384-390.

11 M. Montalti, A. Credi, L. Prodi and M. T. Gandolfi, *CRC Press, Taylor & Francis Group, Boca Raton, FL 33487-2742*, 2006.
